# The Synthesis, Fungicidal Activity, and in Silico Study of Alkoxy Analogues of Natural Precocenes I, II, and III

**DOI:** 10.3390/molecules27217177

**Published:** 2022-10-24

**Authors:** Khaled M. A. Ramadan, Hossam S. El-Beltagi, Zafar Iqbal, Eslam S. A. Bendary

**Affiliations:** 1 Central Laboratories, King Faisal University, Al-Ahsa 31982, Saudi Arabia; 2 Biochemistry Department, Faculty of Agriculture, Ain Shams University, Cairo 11241, Egypt; 3 Agricultural Biotechnology Department, College of Agriculture and Food Sciences, King Faisal University, Al-Ahsa 31982, Saudi Arabia; 4 Biochemistry Department, Faculty of Agriculture, Cairo University, Giza 12613, Egypt

**Keywords:** *Aspergillus niger*, antifungal, chromene, *Rhizoctonia solani*, molecular docking, polygalactouronases, precocence

## Abstract

This study aimed to synthesize, characterize, and explore the eco-friendly and antifungal potential of precocenes and their derivatives. The organic synthesis of the mono-O-alkyl-2,2-dimethyl 2*H*-1-chromene series, including the natural product precocene I, and the di-*O*-alkyl 2,2-dimethyl-2*H*-1-chromene series, including the natural 2*H*-1-chromenes precocenes II and III, was achieved. The synthetic compounds were subjected to spectroscopic analysis, ^1^HNMR,^13^CNMR, and mass characterization. The antifungal activity of synthesized precocenes I, II, and III, as well as their synthetic intermediates, was evaluated by the poison food technique. Precocene II (EC_50_ 106.8 µg × mL^−1^ and 4.94 µg mL^−1^), and its regioisomers **7a** (EC_50_ 97.18 µg × mL^−1^ and 35.30 µg × mL^−1^) and **7d** (EC_50_ 170.58 × µg mL^−1^), exhibited strong fungitoxic activity against *Aspergillus niger* and *Rhizoctonia solani*. Some of the novel chromenes, **11a** and **11b**, which had never been evaluated before, yielded stronger fungitoxic effects. Finally, docking simulations for compounds with promising fungitoxic activity were subjected to structure–activity relationship analyses against the polygalactouronases and voltage-dependent anion channels. Conclusively, precocenes and their regioisomers demonstrated promising fungitoxic activity; such compounds can be subjected to minor structural modifications to yield promising and novel fungicides.

## 1. Introduction

The problems associated with excessive pesticide use, particularly in developing countries such as Egypt, have prompted scientists to investigate eco-friendly and novel methods of plant protection. Secondary metabolites of higher plants, insects, and animals have been investigated as potential natural fungitoxicants for plant disease management. These natural chemicals are physiologically active and are produced to aid in pathogen defense, interspecies competition, and reproductive process facilitation. The biological activity of natural products against phytophagous insects, such as pesticides and crop protection compounds, has gained significant attention over the last couple of decades. Natural products are now considered as promising alternatives to the current arsenal of synthetic compounds [1]. The heterocyclic benzopyran skeleton containing oxygen, particularly its structural derivatives such as precocenes I, II, and III, constitutes a privileged group of compounds. These compounds are found in a wide range of phytochemical classes of natural products [2,3,4,5,6]. These compounds have excellent and broad-spectrum antibacterial, antifungal, antiviral, and insecticidal activities. Additionally, they have pharmacological properties, such as anti-inflammatory, anticancer, and antioxidant activities [7,8].

Phytopathogenic fungi, *Rhizoctonia solani,* and *Aspergillus niger*, especially the former, have long been recognized as a major threat to agricultural production around the globe, causing massive yield losses and control difficulties. *R. solani* invades the stem and root of the host plants, including the maize, rice, soybean, potato, sugar beet, and wheat [9,10], causing necrotic lesions from which it obtains nutrients for development and growth. The cells of the infected plant died prior to hyphae invading them [11], implying that the fungus produces such substances that diffuse and condition the host plant into susceptibility. How this is achieved is presently unknown, though some studies have identified phenolic compounds, phenylacetic acid, and derivatives or carbohydrate-based host-specific toxins in the rice-*R. solani* AG1–1A interaction. *R. solani* employs an active mechanism to suppress host defenses and condition the host plant for susceptibility via the production and secretion of proteinaceous toxins and effectors [12,13]. *A. niger* (black mold), the most common *Aspergillus* fungal species, is a common food contaminant which causes black mold disease on certain fruits and vegetables. When inhaled or consumed in food and feed contaminated with high levels of aflatoxin, *A. niger* causes aflatoxicosis.

Organic synthesis has enabled scientists to produce the most intriguing molecules in living nature and their variants in the laboratory by developing novel strategies. Furthermore, using sophisticated catalytic reactions and appropriately designed synthetic processes, the synthesis of natural molecules, their analogues, and a plethora of other organic molecules with vast utilities has become routine. Such molecules aid biology by serving as biological tools. The privileged benzopyran structures, chromenes, chromanes, and chromanones, are phenomenal in developing novel drugs. Precocenes I and II, isolated from *Ageratum* spp. and *Tagetes* spp. (the *Asteraceae* family), have antifungal attributes; insect growth regulators; and allelopathic inhibitory effects on barnyard grass, ryegrass, and bidens clover [14,15,16]. Cumanensic acid, a novel chromene isolated from *Piper* cf. *cumanense Kunth*. (the *Piperaceae* family), was found to have potent antifungal activity against *Fusarium oxysporum* and *Botrytis cinerea* [17]. These findings suggested that the privileged benzopyran structures can be used as a promising scaffold for developing novel antifungal compounds. A unique organic synthesis approach was opted in this study to prepare the di-O-alkyl 2,2-dimethyl-2*H*-1-chromene series, including those of the natural 2*H*-1-chromenes precocenes II and III. In addition, the mono-*O*-alkyl-2,2-dimethyl 2*H*-1-chromene series was synthesized, including the natural product precocene I.

Our knowledge of the precise molecular mechanism by which precocenes inhibit fungal growth is scarce. To decipher the role of precocenes at the molecular level, structure–activity relationship analyses were performed on compounds with promising fungitoxic activity against endopolygalactouronases (PGUs) and voltage-dependent anion channels (VDACs). PGUs, produced by a wide range of organisms, including fungi, bacteria, and plants, perform various physiological and pathological functions, such as cell wall degradation and its remodeling. To penetrate and colonize plant tissues, phytopathogenic microbes deploy PGU as part of their offensive arsenal [18]. PGUs encoded by fungi, with an endo mode of action, catalyze the fragmentation and solubilization of pectin polymers by cleaving the internal bonds of homogalacturonan. Plants, on the other hand, use PGUs in a variety of processes, including growth [19], fruit softening [20,21], root formation [22], organ abscission, and pollen development [23].

The voltage-dependent anion channel (VDAC), a mitochondrial outer membrane protein (also referred to as mitochondrial porin), is responsible for ATP, NADH, and other low-molecular-weight metabolite fluxes [24]. The VDAC interacts with different proteins, such as glucokinase, and plays a role in the release of proapoptotic cytochrome c and superoxide from mitochondria into the cytosol [25].

To the best of our knowledge, 2*H*-1-chromene compounds (precocenes I, II, and III), and their regioisomers, which aimed to achieve antifungal activity, have rarely been reported [8,26]. The selective fungitoxic effect of natural chromene, precocene II (6,7-dimethoxy-2,2-dimethyl-2*H*-1-chromene), isolated from the volatile fraction of *Ageratum hostoIianum* plants against the soil-born fungi *R. solani* and *Phytophthra megasperma*, triggered the idea behind this article [16]. Following our ongoing interest in the discovery and development of novel antifungal candidates, the synthesis of the natural 2*H*-1-chromene compounds, precocenes I, II, and III, as well as the synthesis of a series of regioisomers of those natural products, was carried out to investigate their antifungal activity prospects and their mass production via organic synthesis. In this present work, the naturally occurring precocenes and their analogues were synthesized, characterized, and evaluated for their fungitoxic activity against *A. niger* and *R. solani*.

## 2. Results and Discussion

### 2.1. Synthesis of Natural 2H-1-Chromene Compounds and Their Alkoxy Regioisomer Analogues

**Scheme 1:**
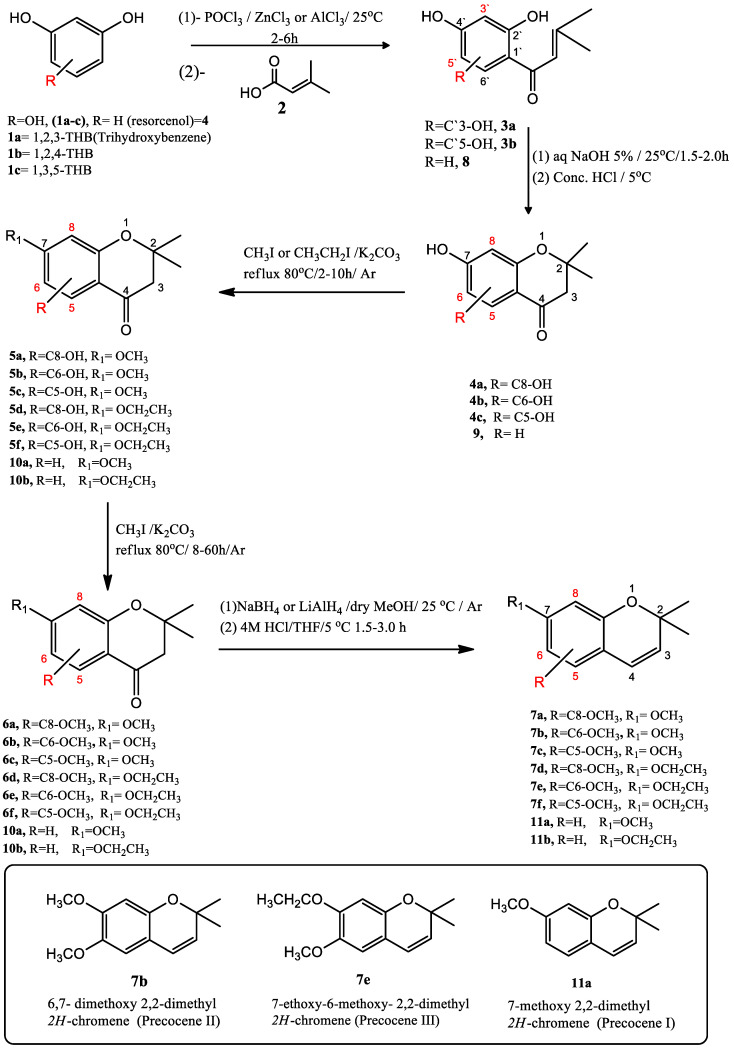
Synthetic diagram of dialkoxy 2,2-dimethyl-2*H*-benzopyrane and monoal-koxy-2,2-dimethyl-2*H*-1-benzopyran. The red marked substituent R group is positioned on carbon atoms marked by red numbers.

Natural chromene (precocenes II and III) analogues and their regioisomers were synthesized according to Timár and Jaszberenyi [27], with some modifications as illustrated in Scheme 1.

Our initial efforts were primarily focused on choosing one of the many strategies aimed at constructing a benzopyrane structure. The following synthetic strategy was opted: (i) the construction of benzopyranone compounds (synthetic precursors) and benzopyrane compounds in high yields and high purities to study their fungitoxic effects and (ii) the preparation of regioisomers of natural chromenes and their synthetic intermediated chromanones to test the effect of changing the position and type of substituent at the aromatic ring on their potential fungitoxic activity. Finally, the quality, quantity, and structure of the synthesized compounds were assessed using ^1^HNMR and ^13^CNMR spectroscopic data.

The first step in the synthesis process was the preparation of compounds **3a** (1-[2′,3′,4′-trihydroxyphenyl]-3-methyl-1-Oxo-buta-2-ene), **3b** (1-[2′,4′,5′-trihydroxyphenyl]-3-methyl-1-Oxo-but-2-ene), and **8** (1-[2′, 4′-dihydroxyphenyl]-3-methyl-1-oxo-buta-2-ene) via Friedel–Crafts acylation mechanism. The recorded spectral data of ^1^HNMR elucidate the position of three phenolic OH groups. The proton chemical shift after treatment with D_2_O showed the absorbance peaks at δ8.6, 10.5, and 13.29 ppm and 8.75, 10.39, and 12.85 ppm for the deuterated compounds **3a** and **3b**, respectively, while it was 10.57 and 13.26 for compound **8**. The spectroscopic findings agreed with the earlier reports [28,29]. In the second step, the heterocyclic benzopyranone compounds, **4a** (7,8-dihydroxy 2,2-dimethyl chroman-4-one), **4b** (6,7-dihydroxy 2,2-dimethyl chroman-4-one), **4c** (5,7-dihydroxy 2,2-dimethyl chroman-4-one), and **9** (7-hydroxy 2, 2-dimethyl chroman-4-one), were obtained via intermolecular Michael addition–cyclization. The proton chemical shift δ of compounds **4a**, **4b**, **4c**, and **9** indicated that there were six equivalent protons (two CH_3_ groups) detected at δ1.39 (singlet), and two protons of CH_2_ group were detected up-field at δ2.66 (singlet). In compound **4a**, two aryl protons split doublet (J = 6.8 Hz), H-5 and H-6, were detected at δ7.11 and δ6.45, respectively. A broad absorption peak for the OH-8 proton was detected at 8.48 (singlet), while the OH-7 proton was detected down-field at δ 9.97 (singlet). Similarly, the proton chemical shifts of the other two regioisomers, **4b** and **4c**, meant that the ^1^HNMR spectrum could position the phenolic OH groups, 9.04 (broad, 1H, OH-6), 10.14 (broad, 1H, OH-7), 10.73 (singlet [s], 1H, OH-5), and 12.11 (s, 1H, OH-7), respectively. In mono hydroxychromanone **9**, phenolic OH up-fielded at δ 5.81 (broad, 1H, OH-7). The obtained spectroscopic results for the chroman-4-one skeleton corroborated to the previous reports [30,31,32]. In the third step, the corresponding mono and dihydroxy 2,2-dimethyl chroman-4-one intermediates, **9** and **4a**–**c**, were regioselectivity alkylated to yield the 7-O-alkyl analogues. The proton chemical shift of the free OH group of **5a** and **5b** was detected at δ 5.42 (broad) and δ 5.30 (s), respectively, due to the phenolic OH-8 and OH-6. The absorbance of OH-5 in **5c** was detected much more down-field at δ 12.05 (singlet) due to the de-shielding effect of the adjacent C=O group. The proton chemical shift confirmed the transformation of hydroxyl to-OCH_2_-CH_3_ at position 7 in compounds **5d**−**f**. There was absorptance at δ 1.4–1.52 (tertiary) for protons of CH_3_, J = 7.3 HZ. Protons of the CH_2_ group in ethoxy were detected at δ 4.02–4.25 and the peak was multiplied into a quartet as a splitting effect of three protons of the neighboring CH_3_ group, J = 7.3 HZ. The proton chemical shifts of OCH_3_ and OCH_2_CH_3_ in compounds **10a** and **10b** were up-fielded at 3.84 (s, 3H, OCH_3_) and 4.05 (quartet [q], 2H, J = 6.0 Hz, OCH_2_CH_3_). The spectrum of ^1^HNMR of compounds **6a**–**f** proved the methylation of the free OH group in corresponding compounds **5a**–**f**. The absorbance down-field at δ 3.0–3.9 (s, 3H, OCH_3_) detected the singlet protons of the transformed methoxy group. In the final two steps of chromanone compounds, the ketonic group was selectively reduced to a secondary hydroxyl group and then dehydrated under mild conditions to produce reduced mono and dialkoxy–2,2–dimethyl 2*H*–1–chromene compounds **11a**–**b**. For the disubstituted compounds **7a**–**f**, the overall obtained average yield was 53.0–61.0%, and for mono-substituted chromenes **11a** and **11b**, the overall obtained yields were 62.5% and 87.3%, respectively. The obtained spectroscopic data of ^1^HNMR and ^13^CNMR proved that the natural chromenes (**7b**, **7e**, and **11a**) and their analogues were pure enough to be used in the antifungal evaluation. Proton and carbon chemical shifts verified the 2H-chromene structure and its substituents. A vinylic proton (H-3) at C-3 was detected at δ 5.4–5.5 and split into doublet with J = 9.9 Hz, while H-4 was detected at δ 6.01–6.28 (doublet[d], J = 9,9 Hz). Carbon chemical shifts of **7a**–**f** and **11a**–**b** confirmed the map of the carbon skeleton and different substitutions at the benzopyrane structure. The obtained spectroscopic data results agreed with several earlier reports of synthetic approaches to the chromenes ring structure [33,34,35,36,37,38].

### 2.2. Evaluation of Antifungal Activity

Natural chromenes and its alkoxy derivatives, obtained by chemical synthesis, were evaluated for antifungal activities against the selected toxigenic fungi. The novelty of these compounds relies on the simple and straightforward synthesis and the absence of halogenated derivatives. This latter property makes these compounds more environmentally friendly than commercial fungicides. The antifungal activity of all synthesized natural chromenes, precocenes I, II, and III, as well as their analogues and synthetic intermediates, was assessed in vitro against *R. solani* and *A. niger* using the poison feed technique. Mycelial growth inhibition was studied at the effective concentrations of 50% (EC_50_) and 90% (EC_90_) (Table 1). Almost all the compounds exhibited pronounced fungitoxic effects at varying levels. The EC_50_ and EC_90_ values of **3a** against *A. niger* were 614.56 and 833.66 µg × mL^−1^, respectively, and values of **3b** were 1023.29 and 1591.74 µg × mL^−1^. Both **3a** and **3b** compounds are regioisomers with different positions of the hydroxy phenolic groups. Nonetheless, the enhanced antifungal activity of **3a** could be attributed to the presence of the α,β-unsaturated bond in the side chain adjacent to the C=O group [39]. Moreover, the position of the phenolic OH group was presumed to play an important role in bioactivity. The chemical nature of α,β-unsaturated aldehydes, as well as some of their toxicological effects, was based on their ability to function as direct-acting alkylating agents. Carbonyl carbon is an electrophilic site that readily reacts with nucleophiles [40,41]. Nucleophilic attack on the carbonyl moiety by primary amines, thiols, and possibly alcohols results in the formation of substituted amines, known as Schiff bases and hemiacetals, under physiological conditions. Secondary amine or thiol attack on the initial adducts can cause protein–protein, DNA–protein, or DNA–DNA cross-linking [42]. So, we speculate that the hyper-antifungal activity of **3a** is due to the presence of a carbonyl moiety with a double bond.

The EC_50_ value of **4c** was lower than **4a** and **4b**. The free phenolic OH groups at positions 5 and 7 in **4c** boosted its antifungal activity, nearly doubling the activity compared to **4a** and **4b** (Table 1). It is worth noting that when the phenolic OH group in position 7 of **4c** was alkylated, the resulting compound **5c** demonstrated a higher antifungal activity. Likewise, methylating the phenolic OH group at position 6 in **4b** enhanced the antifungal activity of the resulting product **5b**. On the contrary, the antifungal activity of **5a** was lowered dramatically. It was also observed that **5c** showed much higher activity than its **5f** analogue. When a methoxy group in **5a** was substituted by an ethoxy group in **5d**, the antifungal activity increased nearly sixfold. A similar observation was noticed in **5e**. It could be inferred that changing the alkyl group in such compounds could improve their antifungal activity. Abrunhosa et al. [43] evaluated the antifungal activity of chromene dimers and found that the growth and activity of *Asprgillus* spp. in producing ochratoxin A varied with the change in chromene side chain structure, specifically when the H on benzene ring was replaced by the OCH_3_ group.

The EC_50_ values of **6a**–**f** were highly variable, with **6c** having the lowest (398.32 µg × m L^−1^) and **6b** having the highest (1191.30 µg × m L^−1^) (Table 1). Compound **6c** was found to have higher antifungal activity than its regioisomers **6a** and **6b**. On the other hand, **6d** had a strong inhibitory effect on the mycelial growth of *A. niger*, compared to **6e** and **6f.** An earlier report on the antifungal activity of the structurally related compound, 2-phenyl-chromen-4-one, showed antifungal activity at high concentrations against *Penicillium* spp. and *Colletotrichum* spp. [44]. The compound 2-(4-ethoxy-phenyl)-chromen-4-one is a potent inhibitor of energy-dependent fungicide efflux transporters in *Pyrenophora triticirepentis* [45]. Using this compound in conjunction with fungicides reversed *P. triticirepenti* fungicide resistance.

The fungitoxic effect of **7a** and **7b** was strong against *A. niger*, with EC_50_ values of 97.18 µg × mL^−1^ and 106.8 µg × mL^−1^, respectively (Table 1; Supplementary Figure S1). Meanwhile, **7c** showed no activity against *A. niger*. Additionally, the reduced antifungal activity of **7d**–**f** was observed, indicating that substituting a methoxy group with an ethoxy group resulted in the compromised antifungal activity of **7e** and **7f**, while **7d** remained unaffected. The natural product precocene II had antifungal activity with an EC_50_ value of 89.13 µg × mL^−1^ [16,46,47]. Chromene inhibited mitochondrial respiration in wild-type yeast [48] and *F. graminearum* by interacting with the VDAC, resulting in enhanced superoxide levels in mitochondria [49].

The antifungal activity of **9** was lower than **8** (Table 1 and Table 2). The compound **10a** inhibited the mycelial growth of *A. niger* with an EC_50_ value of 221.31 µg × mL^−1^ and an EC_90_ value of 799.7 × µg mL^−1^ (Table 1). The EC_50_ value of **10b**, on the other hand, was found to have no activity against the tested fungus, while its EC_90_ value could not be inferred. The compound **11a** (precocene I) was found to have nearly twice the antifungal activity of its analogue **11b**. Notably, precocene II (**7b**) inhibited the mycelial growth of *A. niger* almost three times more effectively than precocene I (**11a**) at the EC_50_. Our findings corroborated the previous reports that demonstrated a higher antifungal activity of precocene I, extracted from natural plant extracts, than precocene II [50,51,52]. Precocene II inhibited trichothecene production in *F. graminearum* by increasing superoxide levels in mitochondria after interacting with VDACs [49,53].

Based on the results obtained for the synthesized compounds against *R. solani*, two general observations were made: (i) the ethoxy group (which is more bulky, more electron-donating, and less polar) enhanced the fungitoxicity of the chroman-4-one compound more than methoxy group; and (ii) the more substituents on the benzene ring of chroman-4-one structure, the more active as a fungitoxic agent.

Among **7a**, **7b**, and **7f**, both regioisomers **7a** (EC_50_ 35.30 and EC_90_ 199.94 µg × mL^−1^) and **7b** (EC_50_ 4.94 and EC_90_ 74.69 μg × mL^−1^) demonstrated a higher fungitoxic effect against *R. solani* than **7f** (Table 2, Supplementary Figure S2). Similar findings have been demonstrated earlier by Ramadan et al. [16], who found that precocene II, extracted from *A. houstonianum* essential oil, had promising EC_50_ values, 2.0 µg × mL^−1^ and 38.07 × µg mL^−1^, against *R. solani* and *P. megasperma*, respectively. It was suggested that the natural precocene II has a very strong fungitoxic activity against two species of soil-borne disease fungi. The current findings, along with the earlier reports, have opened an array of myriads for using natural fungicides in managing root rot diseases, which cause substantial losses to the agricultural economy.

Likewise, **11a** (EC_50_ 10.39 µg × mL^−1^) was tenfold more fungitoxic than its analogue **11b** (EC_50_ 116.47 µg × mL^−1^) (Table 2). The outstanding fungal inhibition activity of **11a** and **11b** has never been reported before. The inhibition of the mycelial growth of *R. solani* observed for compound **8** was higher (EC_50_ 76.62 µg × mL^−1^) than **3a** (EC_50_ 123.77 µg × mL^−1^). Among the monoalkoxy compounds, **5c** showed strong antifungal activity (EC_50_ 46.88 µg mL^−1^ and EC_90_ 146.16 µg × mL^−1^). Meanwhile, **6f** and **6d** induced fungal inhibition at higher concentrations, with EC_50_ of 204.55 and 720.24 µg × mL^−1^, respectively, and EC_90_ values of 245.42, 406.91 µg × mL^−1^, respectively (Table 2). The observed results revealed that the presence of different moieties, including the free hydroxyl group, the methoxy group, or the ethoxy group in a different position at the benzene ring, strongly enhances the fungitoxic activity of chromenes and chromanones. In a bioassay using the yeast *Saccharomyces cerevisiae*, the chromene isolated from *Eulypa lata* either caused death or strongly inhibited the yeast growth [54]. Additionally, a respiratory assay using 2,3,5-triphenyl tetrazolium revealed that eutypinol and eulatachromene inhibited mitochondrial respiration in wild-type yeast and significantly reduced the cell growth of a mutant *S. cerevisiae*, lacking a thioredoxin peroxidase [54].

From the previous studies, the synthetic benzopyrones and their derivatives exhibited outstanding antifungal activity against different species of phytopathogenic fungi, e.g., *Trichophyton longifusus*, *T. longifusum*, *Candida albicans*, and *A. flavus* [55,56]. Another chromene derivative, 5-hydroxy-6-acetyl-2-hydroxymethyl-2-methyl chromene, had a strong fungitoxic effect against *C. albicans* and *Cryptococcus neoformans* [57]. The compounds [3-(s)-4,6-dihydro-8-methoxy-3,5-dimethyl-6-oxo-3*H*-2-benzopyrane], [1-(S),3-(S)-6-hydroxy-1,8-dimethoxy-3,5-dimethylisochroman] and [1-ethoxy-6-hydroxy 8-methoxy-3,5-dimethyl isochroman] inhibited the mycelial growth of *Lasiodiplodia theobromae* at 100 mg × mL^−1^ concentration [58]. Sariaslani et al. [59] studied the biotransformation of precocene II by microbial enzymes in *Streptomyces griseus* using ^18^O_2_ incorporation studies, concluding that precocene II was transformed into three major metabolites, including the mono-oxygenase enzyme, and could introduce possible evidence of interaction between the heterocyclic oxygen-containing compounds with the enzymatic systems and the mitochondrial respiration. Conner and Beuchat [60] and Knobloch et al. [61] proposed a mechanism for the fungitoxic effect of heterocyclic oxygen compounds, such as chromenes and chromanones, which affect the cell membrane causing increased permeability or interference with a variety of enzyme systems. Aqueous and ether extracts of Ageratum leaves, containing precocene II as a major constituent, inhibited fungal growth by halting the formation of germ tubes by spores in the presence of the tested fungi, which is crucial for the microorganism’s survival because new hyphae formation can only begin with the germ tubes [62]. Precocene II retards fungal growth or stops the release of mycotoxins, such as aflatoxins (B1, B2, G1, and G2) and trichothecenes. It can reduce mRNA synthesis of deoxynivalenol, a contaminant released by *F. graminearum* that reduces grain utilization [63,64].

### 2.3. Molecular Docking

Molecular docking analyses of different PGUs and VDACs against chromenes and chromanones are shown in Figures 5 and 6. Precocene has previously been shown to inhibit fungal growth by interacting with PGUs [16]. Therefore, in molecular docking studies, various PGUs from *A. niger* and *R. solani* were included. Meanwhile, the VDAC was targeted by precocene II to affect VDAC gating (by gate closing) and inhibit microbial growth [49].

**Figure 1:**
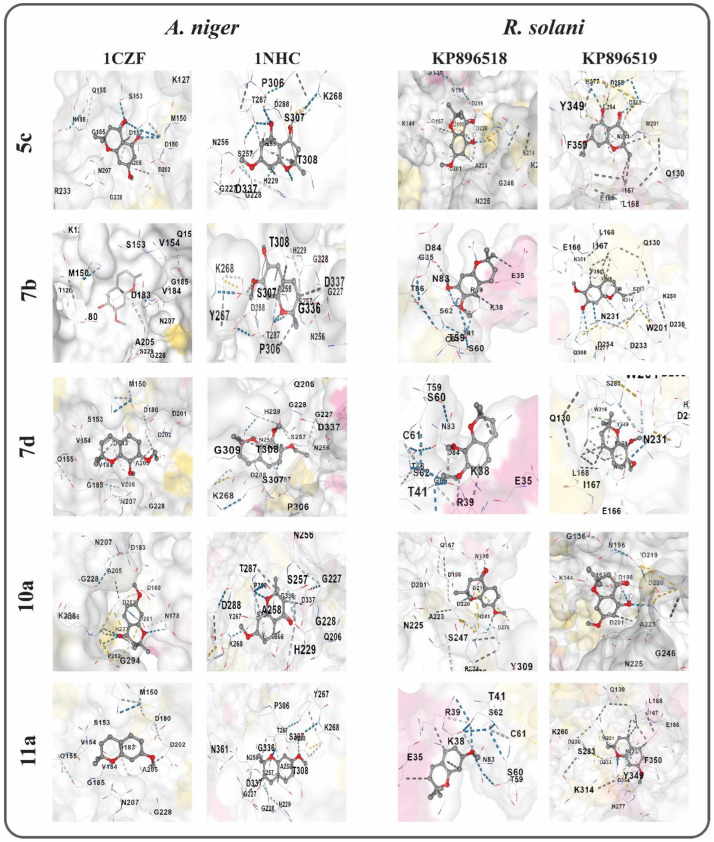
Molecular docking 3D binding models of the tested compounds against polygalactouronases encoded by *A. niger* (1CZF and 1NHC) and *R. solani* (KP896518 and KP896519).

**Figure 2:**
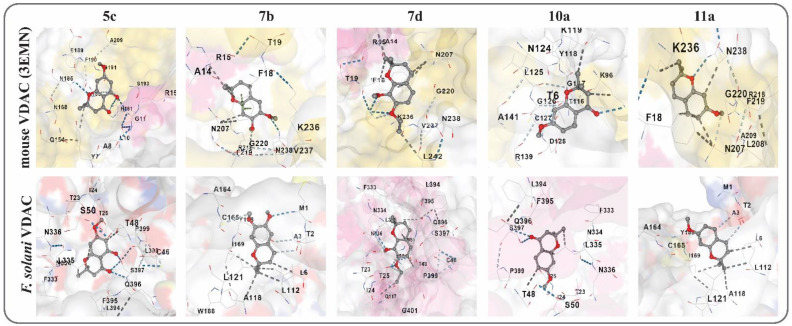
Molecular docking 3D binding models of the tested compounds against voltage-dependent anion channels (VDACs) encoded by mouse and *F. solani*.

In CB-dock2, the top five cavity sizes were identified, and Vina scores for binding were obtained for each of the cavity, but just one structure with the lowest Vina score and amino acid residues involved with natural inhibitor was presented. Generally, the three chromenes showed less binding affinity/docking scores as compared to the chromanones. More amino acid (AA) residues of *R. solani* were found interacting with the used ligands than *A. niger* (Figure 1 and Figure 2; Supplementary Table S1).

Chromenes docked to *A. niger*-encoded PGUs, 1CZF and 1NHC, had lower docking scores. Two chromenes, **11a** (precocene I) and **7b** (precocene II), docked against 1CZF with a cavity volume of 246 and Vina scores of −5.4 and −5.8, respectively. Meanwhile, both docked to 1NHC at a cavity volume of 126 and a Vina score of −6.4. The third chromene, 7d, docked to 1CZF at a cavity volume of 483 with a Vina score of −5.8, and docked to 1NHC at a cavity volume of 1901 with a Vina score of −6.7 (Supplementary Table S1). Likewise, the two chromanones, **5c** and **10a**, docked to 1CZF at a cavity volume of 764 and Vina scores of −6.0 and −5.6, respectively. Both these chromanones docked to 1NHC at a cavity volume of 1901 with Vina scores of −7.2 and −7.1, respectively (Supplementary Table S1).

Chromenes showed higher binding affinities to *R. solani*-encoded PGUs, PGU1, and PGU2. Three chromenes, **11a**, **7b**, and **7d**, docked to PGU1 with a cavity volume of 359 with Vina scores ranging from −5.6 to −6.2. Likewise, the two chromanones, **5c** and **10a**, docked to PGU1 at a cavity volume of 359 with Vina scores of −6.4 and −7.0, respectively. Both these chromanones docked to PGU2 at a cavity volume of 1417 with Vina scores of −6.7 and −6.8, respectively (Supplementary Table S1).

The molecular docking analysis demonstrated that **11a** and **7b** docked to *F. solani*-encoded VDACs and mouse-encoded VDACs at different cavities; the former docked at a cavity volume of 3839 and the later docked to two different cavity volumes (139 and 933). *F. solani*-encoded VDACs had smaller Vina scores (−6.3) than two chromanones and a chromene with Vina scores of −6.5 and −7.0, respectively (Supplementary Table S1). Although both VDACs are structurally similar, nonetheless, both docked in different places with synthetic compounds. Generally, **5c**, **7b**, **7d**, and **11a** docked to PGUs at similar positions in both *A. niger* and *R. solani* by interacting with the same AA residues (Figure 1). Meanwhile, in the case of VDACs, a different trend was observed; **5c** and **10a** docked to mouse VDACs at the same position, interacting with the same AA residues, while **7b**, **7d**, and **11a** docked to another position. However, in the case of *F. solani* VDACs, 5c, 7d, and 10a docked at the same position, while **7b** and **11a** docked at different positions (Figure 2).

To decipher the precise role of chromenes and their derivates as promising alternative fungicides at the molecular level, it is a prerequisite to identify their exact bioreceptor. Nevertheless, the preceding findings could be validated in vivo using PGUs, VDACs, or other fungal enzymes, and could be of interest for futuristic studies.

Abbreviations used in the figure are 7-methoxy, 5-hydroxy 2,2-dimethyl chroman-4-one (**5c**), 6,7- dimethoxy 2,2-dimethyl 2*H*-chromene (**7b**), 7-ethoxy-8-methoxy 2,2-dimethyl 2*H*-chromene (**7d**), 7-methoxy 2,2-dimethyl chroman-4-one (**10a**), and 7-methoxy 2,2-dimethyl 2*H*-1-chromene (**11a**). Meanwhile, the amino acids are represented by their standard letters.

Abbreviations used in the figure are 7-methoxy, 5-hydroxy 2,2-dimethyl chroman-4-one (**5c**), 6,7-dimethoxy 2,2-dimethyl 2*H*-chromene (**7b**), 7-ethoxy-8-methoxy 2,2-dimethyl 2*H*-chromene (**7d**), 7-methoxy 2,2-dimethyl chroman-4-one (**10a**), and 7-methoxy 2,2-dimethyl 2*H*-1-chromene (**11a**). Meanwhile, the amino acids are represented by their standard letters.

## 3. Materials and Methods

### 3.1. Chemicals and Spectroscopy

Trihydroxybenzenes (1,2,3-, 1,2,4-, and 1,3,5-), 1,3-dihydroxybenzene, 3-methyl-but-2-eneoic acid, phosphorus oxichloride, zinc chloride, aluminum chloride, 99% methyl iodide, 99% ethyl iodide, sodium borohydride (NaBH_4_), and lithium aluminum hydride (LiAlH_4_) were obtained from Sigma-Aldrich, Canada, and were of analytical grade. Solvents used in organic syntheses were of HPLC grade and subjected to distillation and purification prior to use. Plastic-backed F-254 (thin-layer chromatography (TLC, Fischer, Waltham, MA, USA), 200 µm of silica gel, and silica gel (32–63 µm/60 Å) were used in column chromatography. All the synthetic compounds were characterized with GC-MS (Agilent HP5970, Santa Clara, CA, USA), LC-MS (Agilent, InfinityI, 1100, Santa Clara, CA, USA), NMR (Bruker Avance 500 MHz, Billerica, MA, USA), and FTIR (Bruker Tensor 27 infrared spectrometer, Billerica, MA, USA), and the melting points were measured by a melting point apparatus (Fisher–Johns melting point, Santa Clara, CA, USA).

### 3.2. General Procedure of the Synthesis of Chromene Analogues

#### 3.2.1. Synthesis of 1-[Trihydroxyphenyl]-3-methyl-1-oxo-buta-2-ene (**3a**–**b**) and 1-(2′,4′-dihydroxyphenyl)-3-methyl-1-oxo-buta-2-ene (8)

Next, 100.2 g (1 mole) of 3-methyl-but-2-eneoic acid and 1319.7 g (780.1 mL, 8.6 moles) of phosphorus oxychloride were stirred for 10 min. When the reaction mixture cooled down to the 25 ± 2 °C, the flask was charged with 201.5 g (1.4 moles) of zinc chloride. To this mixture, 126.14 g (1.0 mole) of 1,2,3-trihydroxybenzenes **1a** and 1,2,4-trihydroxybenzenes **1b** was added. Then, the reaction mixture was stirred at 25 °C for 6 h to yield **3a** (1-[2′,3′,4′-trihydroxyphenyl]-3-methyl-1-oxo-buta-2-ene) and stirred for 2 h to yield **3b** (1-[2′,4′,5′-trihydroxyphenyl]-3-methyl-1-Oxo-but-2-ene). Compound **8** (1-(2′,4′-dihydroxyphenyl)-3-methyl-1-oxo-buta-2-ene) was synthesized by mixing 55.06 g (0.5 moles) of resorcinol (1,3-dihydroxybenzene), 50.10 g (0.5 moles) of 3-methyl-but-2-enoic acid, and 613.8 g (363.2 mL, 4 moles) of POCl_3_ at 25 °C under inert gas (argon) conditions, and then 80 g (0.6 moles) of dry AlCl_3_ was added to it to yield **8** [65]. The reaction was monitored by TLC (n-hexane: ethyl acetate 1:1); upon completion, the mixture was poured onto crushed ice and then filtered. The crude solid dried under reduced pressure and the product was obtained by re-crystallization from the ethanol–water (95:5) system to yield **3a**, **3b**, and **8**, respectively. The reaction yield was calculated as a percentage of the actual weight of purified **3a** (68.62%), **3b** (50.85%), and **8** (97.4%) to their theoretical weight. The measured melting point (MP) was 137–138 °C for **3a** and 165 °C for **3b** (the reported MP was 162–164 °C [33]), the while MP of **8** was 74 °C. Purified compounds were subjected to structure elucidation; the GC-MS spectral data for compound **3a**, R_t_ = 20.271 min, MS (EI, 70 eV): m/z (%) = 208 (54) [M^+^], 193 (100) [M-Me], 152 (81), 137 (15), 123 (21), 113 (19), 106 (21), 95 (26), 83 (26), 77 (30), 68 (30), 51 (54).

The ^1^HNMR (DMSO-d6) spectral data for compound **3a**: 2.0 [s, 3H, CH_3_), 2.15 (s, 3H, CH_3_), 6.36 [duplet (d)], 1H, J = 9.4 Hz, 5′-H), 6.90 (s, 1H, 2-H), 7.37 (d, 1H, J = 9.4 Hz, 6′-H), 8.6 (s, 1H, 3′OH), 10.5 (s, 1H, 4′OH), 13.29 (s, 1H, 2′OH).

The ^1^HNMR (DMSO-d6) spectral data for compound **3b:** 2.0 (s, 3H, CH_3_), 2.11 (s, 3H, CH_3_), 6.28 (s, 1H, 3′H), 6.76 (s, 1H, 2H), 7.23 (s, 1H, 6′H), 8.75 (broad, 1H, 4′/5′OH), 10.39 (broad, 1H, 4′/5′OH), 12.85 (s, 1H, 2′OH).

The GC-MS spectral data for compound **8:** R_t_ = 17.370 min, MS (EI, 70 eV): m/z (%) = 192 (5) [M+], 177 (100) [M^+^-Me], 137 (31), 108 (7), 81 (11), 69 (11), 51 (16).

The ^1^HNMR (DMSO-d6) spectral data for compound **8**: 2.01 (s, 3H, CH_3_), 2.15 (s, 3H, CH_3_), 6.25 (s, 1H, H-3′), 6.35 (d, 1H, J = 9 Hz, H-5′), 6.91 (s, 1H, H-2), 7.84 (d, 1H, J = 9 Hz, H-6′), 10.57 (s, 1H, OH-4′), 13.26 (s, 1H, OH-2′).

The ^1^HNMR, ^13^CNMR, and mass spectra of all prepared compounds in the experimental section are available in the Supplementary Figures S3–S65.

#### 3.2.2. Synthesis of dihydroxy 2,2-dimethylchroman-4-one (**4a**–**c**) and 7-hydroxy 2, 2-dimethyl chroman-4-one (9)

Afterwards, 100 g (0.48 moles) of **3a** or **3b** compounds or 76.8 g (0.4 moles) of compound **8** was dissolved in 1 L of 5% aqueous NaOH solution (1.25M) and stirred for 1.5–2.0 h at room temperature. After the reaction was completed, the solution was diluted with 500 mL of cold distilled water. Then, the solution was acidified by dropping 36% HCl until pH 1 was obtained. The **4c** (5,7-dihydroxy-2,2-dimethyl-4-chroman-4-one) was prepared directly from 1,3,5-trihydroxybenzene (**1c**), and 162.14g (1.0 mole) of 3-methyl-but-2-eneoic acid and POCl_3_/ZnCl_2_ were mixed. The reaction was set overnight at 25 °C, as described in Section 2.1. The purification of crude products was achieved by column chromatography to obtain **4a** and re-crystallization from ethanol–water (9:1) to obtain **4b**, **4c**, and **8**. The reaction yields 95.1%, 87.6%, 51.8%, and 74.2% and MPs were 197–198, 207, 142, and 173 °C for **4a**, **4b**, **4c**, and **8**, respectively (reported MPs were 199, 208, and 142 °C for **4a**–**c**, respectively, [66]). The GC-MS spectral data of relevant compound **4a** (7,8-dihydroxy 2,2-dimethyl chroman-4-one), R_t_ = 19.763 min, MS (EI, 70 eV): m/z (%) = 208 (39) [M^+^], 193 (40.5), 152 (100) [M^+^-CH_2_=C(CH_3_)_2_], 124 (13.4), 106 (14.1), 113 (19), 106 (14), 95 (8), 68 (11), 53 (13).

The ^1^HNMR (DMSO-d6) spectral data for compound **4a**: 1.39 (s, 6H, 2CH_3_), 2.66 (s, 2H, CH_2_), 6.45 (d, 1H, J = 6.8 Hz, H-6), 7.11 (d, 1H, J = 6.8 Hz, H-5), 8.48 (broad, 1H, OH-8), 9.97 (s, 1H, OH-7).

The GC-MS spectral data for compound **4b** (6,7-dihydroxy2,2-dimethylchroman-4-one), R_t_ = 21.313 min, MS (EI, 70 eV): m/z (%) = 208 (48) [M^+^], 193 (100) [M^+^-Me], 153 (27), 124 (41), 106 (14.1), 96 (20), 78 (11), 51 (13).

The ^1^HNMR (DMSO-d6) spectral data for compound **4b**: 1.34 (s, 6H, 2CH_3_), 2.59 (s, 2H, CH_2_), 6.27 (s, 1H, H-8), 7.04 (s, 1H, H-5), 9.04 (broad, 1H, OH-6), 10.14 (broad, 1H, OH-7).

The GC-MS spectral data for compound **4c** (5,7-dihydroxy-2,2-dimethyl chroman-4-one), R_t_ = 19.790 min, MS (EI, 70 eV): m/z (%) = 207 (100) [M+], 167 (78), 138 (39), 123 (15), 110 (17), 69 (38), 51 (15).

The ^1^HNMR (DMSO-d6) spectral data for compound **4c**: 1.38 (s, 6H, 2CH_3_), 2.77 (s, 2H, CH_2_), 5.82 (multiplet [m], 2H, ArH 6/8), 10.73 (s, 1H, OH-5), 12.11 (s, 1H, OH-7).

The GC-MS spectral data for compound **9**: 7-hydroxy 2, 2-dimethyl chroman-4-one, R_t_ = 16.521 min, MS (EI, 70 eV): m/z (%) = 192 (55) [M+], 177 (100) [M^+^-Me], 137 (61), 108 (56), 95 (5), 80 (20), 69 (18), 51 (21).

The ^1^HNMR (CDCl_3_) of compound **8** had the following attributes; 1.46 (s, 6H, 2CH3), 2.68 (s, 2H, CH_2_), 5.81 (broad, 1H, OH-7), 6.35 (s, 1H, ArH-8), 6.48 (d, 1H, J = 9 Hz, ArH-6), 7.80 (d, 1H, J = 9 Hz, ArH-5).

#### 3.2.3. Synthesis of Monoalkoxy, Monohydroxy-2,2-dimethyl chroman-4-ones (**5a**–**f**) and 7-O-alkyl-2, 2-dimethyl chroman-4-one (**10a**–**b**)

A regioselective alkylation was used to prepare the monoalkoxy and monohydroxy 2,2-dimethylchroman-4-one series **(5a**–**f)**. Under inert gas (argon) conditions, 40 g (0.19 moles) of **4a**–**c** or 19.2 g (0.1 moles) of compound **9** was charged with 100 mL of dry N,N-dimethylformamide for **4a**–**c** or 100 of dry acetone for compound **9**, and then 26.6 g (0.19 moles) or 14.0 g (0.1 moles) of anhydrous K_2_CO_3_ was added to the solutions and stirred at 25 °C for 1h. The resultant solution was refluxed at 80 °C. Then, 29.81 g (19.47 mL, 0.21 moles) of 99% methyl iodide used to yield **5a**–**c** and 15.61 g (10.20 mL, 0.11 moles) used to prepare 10a, or 32.76 g (16.8 mL, 0.21 moles) of 99% ethyl iodide used to yield **5d**–**f** and 17.16 g (8.80 mL, 0.11 moles) used to yield **10b**, was dropped by a programable syringe pump (LAMBDA-FIT, Baar, Switzerland) for over 1.5–6.0 h. The progress of the reaction was monitored by TLC (9:1 hexane: ethylacetate). The reaction times for individual compounds are listed in Table 3.

After completing the reaction, the mixture was cooled to 22 °C ± 2 and the solvent was distilled off. Then, the residue was dissolved in a 5% aqueous NaOH solution and extracted with CH_2_Cl_2_. The organic phase was discarded, and the alkaline aqueous phase was then acidified with conc. HCl (pH 1–2). The filtered-out solid was dried overnight under reduced pressure. The product was subjected to re-crystallization using an ethanol–water (5% water) extraction. The recorded MPs were 78, 108, 54, 141–143, 106–107, and 74 °C for **5a**–**f**, respectively, while the reported MPs were 70, 113–114, 68, 141, 109, and 78 °C, respectively [66,67,68]. The recorded MPs for **10a** and **10b** were 79–81 and 85 °C, respectively.

The GC-MS spectral data for compound **5a** (7-methoxy, 8-hydroxy 2,2-dimethyl chroman-4-one): R_t_ = 21.123 min, MS (EI, 70 eV): m/z (%) = 222 (80) [M+], 167 (100) [M^+^-CH=C(CH_3_)_2_], 207 (90), 192 (8.9), 152 (7.1), 148 (75), 137 (28), 120 (91), 95 (51), 67 (22), 53 (31).

The ^1^HNMR (CDCl_3_) spectral data for compound **5a**: 1.51 (s, 6H, 2CH_3_), 2.72 (s, 2H, CH_2_), 3.96 (s,3H, OCH_3_-7), 5.42 (broad, 1H, OH-8), 6.6 (d, 1H, J = 9.8 Hz, ArH-6), 7.5 (d, 1H, J = 9.8 Hz, ArH-5).

The GC-MS spectral data for compound **5b** (7-methoxy, 6-hydroxy 2,2-dimethyl chroman-4-one): R_t_ = 22.062 min, MS (EI, 70 eV): m/z (%) = 222 (50) [M+], 167 (100) [M^+^-CH=C(CH_3_)_2_], 207 (99), 192 (10), 138 (8), 123 (71), 111 (9), 95 (16), 79 (9), 53 (51).

The ^1^HNMR (CDCl_3_) spectral data for compound **5b**: 1.45 (s, 6H, 2CH_3_), 2.67 (s, 2H, CH_2_), 3.93 (s, 3H, OCH_3_-7), 5.30 (s, 1H, OH-6), 6.41 (s, 1H, ArH-8), 7.36 (s,1H, ArH-5).

The GC-MS spectral data for compound **5c** (7-methoxy, 5-hydroxy 2,2-dimethyl chroman-4-one): R_t_ = 19.920 min, MS (EI, 70 eV): m/z (%) = 222 (46) [M+], 207 (100) [M^+^-Me], 166 (24), 138 (24), 123 (6), 110 (13), 95 (32), 69 (25), 53 (14).

The ^1^HNMR (CDCl_3_) spectral data for compound **5c**: 1.49 (s, 6H, 2CH_3_), 2.72 (s, 2H, CH_2_), 3.84 (s, 3H, OCH_3_-7), 5.9 (d, 1H, J = 2.4 Hz, H-6/8), 6.3 (d, 1H, J = 2.4 Hz, H-6/8), 12.05 (s, 1H, OH-5).

The GC-MS spectral data for compound **5d** (7-ethoxy, 8-hydroxy 2,2-dimethyl chroman-4-one): R_t_ = 21.871 min, MS (EI, 70 eV): m/z (%) = 236 (81) [M+], 152 (100) [M^+^-C_4_H_10_O], 221 (81), 193 (26), 181 (76), 162 (52), 134 (44), 123 (42), 106 (26), 95 (42), 79 (25), 68 (27), 53 (25). The ^1^HNMR (CDCl_3_) spectral data for compound **5d**: 1.52 (triplet (t), 3H, J = 7.3 Hz, OCH2CH_3_), 1.57 (s, 6H, 2CH_3_), 2.77 (s, 2H, CH_2_), 4.25 (quartet (q), 2H, J = 7.3 Hz, OCH_2_CH_3_), 5.49 (broad, 1H, OH-8), 6.63 (d, 1H, J = 9.8 Hz, ArH-6), 7.5 (d, 1H, J = 9.8 Hz, H-5).

The GC-MS spectral data for compound **5e** (7-ethoxy, 6-hydroxy 2,2-dimethyl chroman-4-one): R_t_ = 23.031 min, MS (EI, 70 eV): m/z (%) = 236 (57) [M+], 221 (100) [M^+^-Me], 193 (47), 180 (56), 153 (55), 124 (14), 107 (10), 95 (12), 82 (35), 53 (48).

The ^1^HNMR (CDCl_3_) spectral data for compound 5e: 1.45 (s, 6H, 2CH_3_), 1.49 (triplet [t], 3H, J = 7.3 Hz, OCH_2_CH_3_), 2.66 (s, 2H, CH_2_), 4.13 (q, 2H, J = 7.3 Hz, OCH_2_CH_3_), 5.3 (s, 1H, OH-6), 6.38 (s, 1H, ArH-8), 7.36 (s, 1H, ArH-5).

The GC-MS spectral data for compound **5f** (7-ethoxy, 5-hydroxy 2,2-dimethyl chroman-4-one): R_t_ = 20.623 min, MS (EI, 70 eV): m/z (%) = 236 (58) [M+], 221 (100) [M^+^-Me], 193 (49), 181 (50), 153 (45), 124 (49), 96 (19), 69 (46), 55 (17).

The ^1^HNMR (CDCl_3_) spectral data for compound **5f**: 1.4 (t, 3H, J = 7.3 Hz, OCH_2_CH_3_), 1.5 (s, 6H, 2CH_3_), 2.68 (s, 2H, CH_2_), 4.02 (q, 2H, J = 7.3 Hz, OCH_2_CH_3_), 5.93 (d, 1H, J = 2.4 Hz, ArH 6/8), 5.99 (d,1H, J = 2.4 Hz, ArH 6/8), 12.01 (s,1H, OH-5).

The GC-MS spectral data for compound **10a** (7-O-methyl-2, 2-dimethyl chroman-4-one) R_t_ = 15.119 min, MS (EI, 70 eV): m/z (%) = 206 (31) [M+], 191 (100) [M^+^-Me], 151 (80), 122 (50), 107 (30), 95 (11), 79 (28), 63 (25), 51 (29).

The ^1^HNMR (CDCl_3_) spectral data for compound **10a**: 1.46 (s, 6H, 2CH_3_), 2.68 (s, 2H, CH_2_), 3.84 (s, 3H, OCH_3_), 6.38 (m, 1H, ArH-8), 6.54 (d, 1H, J = 9 Hz, ArH-6), 7.8 (d, 1H, J = 9 Hz, ArH-5).

The GC-MS spectral data for compound **10b** (7-O-ethyl-2, 2-dimethyl chroman-4-one): R_t_ = 15.991 min, MS (EI, 70 eV): m/z (%) = 220 (67) [M+], 205 (100) [M^+^-Me], 165 (49), 136 (69), 108 (49), 80 (17), 69 (18), 51 (20).

The ^1^HNMR (CDCl_3_) spectral data for compound **10b**: 1.42 (t, 3H, J = 6.0 Hz, OCH_2_CH_3_), 1.45 (s, 6H, 2CH_3_), 2.66 (s, 2H, CH_2_), 4.05 (q, 2H, J = 6.0 Hz, OCH_2_CH_3_), 6.35 (m, 1H, ArH-8), 6.52 (d, 1H, J = 9 Hz, ArH-6), 7.78 (d, 1H, J = 9 Hz, ArH-5).

#### 3.2.4. Synthesis of dialkoxy 2,2-dimethyl chroman-4-one (**6a**–**f**)

To obtain **6a**–**f**, 0.1 moles of respective **5a**–**f** in 100 mL of N,N-dimethylformamide was treated with 15.4g (0.11 moles) of anhydrous K_2_CO_3_, and 19.87 g (12.98 mL, 0.14 moles) of CH_3_I (99%) was added using a programmable syringe pump (LAMBDA-FIT, Switzerland), as described in Section 2.2. The reaction time and reaction yield are presented in Table 3. The products were refined after cooling to 20 °C, and then the mix was poured onto crushed ice and extracted twice with CH_2_Cl_2._ The organic layer was washed two times with NaOH 2% and water, and dried over anhydrous sodium sulfate. After removing the solvent, the product was re-crystallized from ethanol. The GC-MS spectral data for compound **6a** (7,8-dimethoxy-2,2-dimethylchroman-4-one): R_t_ = 20.227 min, MS (EI, 70 eV): m/z (%) = 236 (85) [M+], 221 (100) [M^+^-Me], 181 (93), 152 (89), 137 (51), 120 (33), 109 (34), 94 (34), 78 (17), 66 (31), 53 (34), 51 (12).

The ^1^HNMR (CDCl_3_) spectral data for compound **6a**: 1.52 (s, 6H, 2CH_3_), 2.71 (s, 2H, CH_2_), 3.8 (s, 3H, OCH_3_), 3.9 (s, 3H, OCH_3_), 6.6 (d, 1H, J = 10.6 Hz, ArH-6), 7.66 (d, 1H, J = 10.6 Hz, ArH-5).

The GC-MS spectral data for compound **6b** (6,7-dimethoxy-2,2-dimethylchroman-4-one): R_t_ = 17.603 min, MS (EI, 70 eV): m/z (%) = 236 (49) [M+], 221 (100) [M^+^-Me], 205 (5), 181 (79), 165 (32), 137 (33), 109 (10), 109 (10), 53 (37).

The ^1^HNMR (CDCl_3_) spectral data for compound **6b**: 1.47 (s, 6H, 2CH_3_), 2.68 (s, 2H, CH_2_), 3.89 (s, 3H, OCH_3_), 3.89 (s, 3H, OCH_3_), 6.41 (s, 1H, ArH-8), 7.27 (s, 1H, ArH-5).

The GC-MS spectral data for compound **6c** (5,7-dimethoxy-2,2-dimethylchroman-4-one): R_t_ = 18.097 min, MS (EI, 70 eV): m/z (%) = 236 (54) [M+], 180 (100) [M^+^-CH_2_=C(CH_3_)_2_], 221 (25), 152 (59), 137 (62), 109 (20), 95 (18), 79 (16), 53 (25).

The ^1^HNMR (CDCl_3_) spectral data for compound **6c**: 1.44 (s, 6H, 2CH_3_), 2.64 (s, 2H, CH_2_), 3.82 (s, 3H, OCH_3_), 3.88 (s, 3H, OCH_3_), 6.03 (s, 2H, ArH-6/8).

The GC-MS spectral data for compound **6d** (7-ethoxy- 8-methoxy-2,2-dimethyl chroman-4-one): R_t_ = 19.256 min, MS (EI, 70 eV): m/z (%) = 250 (2.6) [M+], 219 (100) [M^+^-OCH_3_], 234 (12), 191 (77), 176 (12), 147 (6), 69 (17), 53 (5).

The ^1^HNMR (CDCl_3_) spectral data for compound **6d**: 1.44 (t, 3H, J = 7.8 Hz, OCH_2_CH_3_), 1.49 (s, 6H, 2CH_3_), 2.68 (s, 2H, CH_2_), 3.84 (s, 3H, OCH_3_-8), 4.1 (q, 2H, J = 7.8 Hz, OCH_2_CH_3_), 6.56 (d, 1H, J = 10.6 Hz, ArH-6), 7.61 (d, 1H, J = 10.6 Hz, ArH-5).

The GC-MS spectral data for compound **6e** (7-ethoxy- 6-methoxy-2,2-dimethyl chroman-4-one): R_t_ = 22.209 min, MS (EI, 70 eV): m/z (%) = 250 (2.6) [M+], 235 (100) [M^+^-Me], 195 (76), 179 (8), 167 (72), 137 (20), 123 (20), 111 (6), 95 (16), 69 (78), 53 (5).

The ^1^HNMR (CDCl_3_) spectral data for compound **6e**: 1.46 (s, 6H, 2CH_3_), 1.55 (t, 3H, J 7.4 Hz, OCH_2_CH_3_), 2.66 (s, 2H, CH_2_), 3.87 (s, 3H, OCH_3_-6), 4.13 (q, 2H, J = 7.4 Hz, OCH_2_CH_3_), 6.39 (s, 2H, ArH-5/8).

The GC-MS spectral data for compound **6f** (7-ethoxy- 5-methoxy-2,2-dimethyl chroman-4-one): R_t_ = 22.962 min, MS (EI, 70 eV): m/z (%) = 250 (51) [M+], 166 (100) [M^+^-C_5_H_8_O], 150 (24), 138 (27), 123 (24), 69 (36).

The ^1^HNMR (CDCl_3_) spectral data for compound **6f:** 1.39 (t, 3H, J = 7.8 Hz, OCH_2_CH_3_), 1.44 (s, 6H, 2CH_3_), 2.64 (s, 2H, CH_2_), 3.87 (s, 3H, OCH_3_-5), 4.03 (q, 2H, J = 7.8 Hz, OCH_2_CH_3_), 6.01 (m, 2H, ArH-6/8).

#### 3.2.5. Synthesis of dialkoxy 2,2-dimethyl *2H*-1-chromene (7**a**–**f**) and monoalkoxy 2,2-dimethyl *2H*-1-chromene (**11a**–**b**)

The 2*H*-1-chromene compounds (**7a**–**f**) were prepared following the reduction and dehydration of the corresponding **6a**–**f** compounds. In total, 0.05 moles of compounds **6a**–**f** was dissolved in 100 mL of dry methanol (absolute) and then treated with 5 g (0.13 moles) of NaBH_4_ dissolved in 50 mL of dry methanol (absolute) using a dropping funnel over one hour under a stream of argon gas condition. Similarly, 7-O-methyl-2,2-dimethylchromene (**11a**; precocenes I) and 7-O-ethyl-2,2-dimethylchromene (**11b**) were synthesized by reducing 0.07 moles of compounds **10a**–**b**, respectively, with 5.31 g (0.13 moles) of LiAlH_4_ in 50 mL of dry tetrahydrofuran (THF). After the reduction process, the reaction was stopped by adding 100 mL of water, and the product was extracted from the mixture with CH_2_Cl_2_. The reaction was monitored by TLC with hexane–ethyl acetate in a ratio of 1:1. Subsequently, the solvent was removed, and the residue was subjected to dehydration with 100 mL (4 mol × L^−1^) of HCl in dry THF at 5 °C using a dropping funnel over 1h. The time required to complete the reduction and dehydration is presented in Table 4. The crude products were extracted by diethyl ether several times, and the combined organic layer was extracted with 5% NaOH solution and then dried over anhydrous Na_2_SO_4_. The product was obtained by column chromatography (9:1 hexane: Et_2_O).

The GC-MS spectral data for compound **7a** (7,8-dimethoxy 2,2-dimethyl *2H*-chromene): R_t_ = 17.348 min, MS (EI, 70 eV): m/z (%) = 220 (16) [M+], 205 (100) [M^+^-Me], 190 (14), 161 (14), 144 (7), 91 (7), 51 (6).

The ^1^HNMR (CDCl_3_) spectral data for compound **7a**: 1.47 (s,6H,2CH_3_), 3.84 (s, 3H, OCH_3_), 3.87 (s, 3H, OCH_3_), 5.5 (d, 1H, J = 9.9 Hz, H-3), 6.26 (d, 1H, J = 9.9 Hz, H-4), 6.41 (d, 1H, J = 7.8 Hz, ArH-6), 6.68 (d, 1H, J = 7.8 Hz, ArH-5).

The ^13^CNMR (CDCl_3_) spectral data for compound 7**a**: 28.1 (2CH_3_), 60.9 (OCH_3_-7), 64.6 (OCH_3_-8), 76.5 (C-2), 105.5 (C-6), 116.3 (C-4a), 120.8 (C-5), 122.3 (C-4), 128.6 (C-3), 138.1 (C-8), 146.7 (C-8a), 152.98 (C-6).

The GC-MS spectral data for compound **7b** (6,7- dimethoxy 2,2-dimethyl 2H-chromene): R_t_ = 17.781 min, MS (EI, 70 eV): m/z (%) = 220 (16) [M+], 205 (100) [M^+^-Me], 189 (13), 161 (15), 91 (10), 77 (10), 69 (11), 51 (6).

The ^1^HNMR (CDCl_3_) spectral data for compound **7b:** 1.41 (s, 6H, 2CH_3_), 3.84 (s, 3H, OCH_3_), 3.85 (s, 3H, OCH_3_), 5.46 (d, 1H, J = 9.9 Hz, H-3), 6.24 (d, 1H, J = 9.9 Hz, H-4), 6.43 (s, 1H, ArH-8), 6.254 (s, 1H, ArH-5).

The ^13^CNMR (CDCl_3_) spectral data for compound **7b**: 27.9 (2CH3), 56.1 (OCH_3_-6/7), 56.7 (OCH_3_-6/7), 76.8 (C-2), 101.2 (C-8), 109.9 (C-5), 113.2 (C-4a), 122.1 (C-4), 128.4 (C-3), 143.2 (C-8a), 147.3 (C-7), 149.8 (C-6).

The GC-MS spectral data for compound **7c** (5,7-dimethoxy 2,2-dimethyl *2H*-chromene): R_t_ = 18.287 min, MS (EI, 70 eV): m/z (%) = 220 (14) [M+], 205 (100) [M^+^-Me], 190 (18), 161 (11), 147 (8), 77 (8), 69 (8), 51 (4).

The ^1^HNMR (CDCl_3_) spectral data for compound **7c**: 1.41 (s, 6H, 2CH_3_), 3.76 (s, 3H, OCH_3_), 3.78 (s, 3H, OCH_3_), 5.40 (d, 1H, J = 10.3 Hz, H-3), 6.01 (m, 2H, ArH-6/8), 6.57 (d, 1H, J = 10.3 Hz, H-4).

The ^13^CNMR (CDCl_3_) spectral data for compound **7c**: 27.7 (2CH_3_), 55.2 (OCH_3_-5/7), 55.4 (OCH_3_-5/7), 76.1 (C-2), 91.4 (C-6), 94.1 (C-8), 104.2 (C-4a), 116.7 (C-4), 125.7 (C-3), 154.7 (C-8a), 156.1 (C-7), 161.01 (C-5).

The GC-MS spectral data for compound **7d** (7-ethoxy-8-methoxy 2,2-dimethyl *2H*-chromene): R_t_ = 18.041 min, MS (EI, 70 eV): m/z (%) = 236 (25) [M+], 219 (100) [M^+^-Me], 191 (51), 176 (37), 115 (10), 91 (20), 77 (12), 51 (10).

The ^1^HNMR (CDCl_3_) spectral data for compound **7d**: 1.41 (t, 3H, J = 6.9 Hz, OCH_2_CH_3_), 1.74 (s, 6H, 2CH_3_), 3.86 (s, 3H, OCH_3_), 4.06 (q, 2H, J = 6.9 Hz, OCH_2_CH_3_), 5.51 (d, 1H, J = 9.5 Hz, H-3), 6.28 (d, 1H, J = 9.5 Hz, H-4), 6.41 (d, 1H, J = 8.6 Hz, ArH-6), 6.65 (d, 1H, J = 8.6 Hz, ArH-5).

The ^13^CNMR (CDCl_3_) spectral data for compound **7d**: 15.4 (OCH_2_CH_3_), 28.41 (2CH3), 61.23 (OCH_3_), 64.94 (OCH_2_CH_3_), 76.77 (C-2), 105.85 (C-6), 116.65 (C-4a), 121.14 (C-5), 122.56 (C-4), 128.93 (C-3), 138.93 (C-8), 147.3 (C-8a), 153.29 (C-7).

The GC-MS spectral data for compound **7e** (7-ethoxy-6-methoxy 2,2-dimethyl *2H*-chromene): R_t_ = 18.826 min, MS (EI, 70 eV): m/z (%) = 236 (25) [M+], 219 (100) [M^+^-Me], 191 (91), 176 (24), 91 (22), 77 (20), 69 (23), 51 (12).

The ^1^HNMR (CDCl_3_) spectral data for compound **7e**: 1.42 (s, 6H, 2CH_3_), 1.48 (t, 3H, J = 6.9 Hz, OCH_2_CH_3_), 3.8 (s, 3H, OCH_3_), 4.06 (q, 2H, J = 6.9 Hz, OCH_2_CH_3_), 5.45 (d, 1H, J = 10 Hz, H-3), 6.24 (d, 1H, J = 10 Hz, H-4), 6.42 (s, 1H, ArH-5/8), 6.54 (s, 1H, ArH-5/8).

The ^13^CNMR (CDCl_3_) spectral data for compound **7e**: 14.9 (OCH_2_CH_3_), 27.8 (2CH_3_), 56.8 (OCH_3_), 64.4 (OCH_2_CH_3_), 76.8 (C-2), 102.3 (C-8), 110.3 (C-4a), 113.3 (C-5), 122.2 (C-4), 128.4 (C-3), 143.1 (C-8a), 147.4 (C-7), 149.5 (C-6).

The GC-MS spectral data for compound **7f** (7-ethoxy-5-methoxy 2,2-dimethyl *2H*-chromene): R_t_ = 16.081 min, MS (EI, 70 eV): m/z (%) = 236 (2) [M+], 219 (100) [M^+^-Me], 234 (20), 191 (66), 176 (24), 147 (19), 91 (17), 69 (22), 51 (10).

The ^1^HNMR (CDCl_3_) spectral data for compound **7f**: 1.38 (t, 3H, J = 6.8 Hz, OCH_2_CH_3_), 1.41 (s, 6H,2CH_3_), 3.79 (s,3H, OCH_3_), 4.0 (q, 2H, J = 6.8 Hz, OCH_2_CH_3_), 5.41 (d, 1H, J = 9.8 Hz, H-3), 6.02 (m, 2H, ArH-6/8), 6.58 (d, 1H, J = 9.8 Hz, H-4).

The ^13^CNMR (CDCl_3_) spectral data for compound **7f**: 14.8 (OCH_2_CH_3_), 28.1 (2CH_3_), 55.4 (OCH_3_), 63.0 (OCH_2_CH_3_), 76.59 (C-2), 92.11 (C-6), 94.82 (C-8), 104.53 (C-4a), 116.79 (C-4), 126.17 (C-3), 155.01 (C-8a), 158.65 (C-7), 160.76 (C-5).

The GC-MS spectral data for compound **11a** (7-O-methyl-2,2-dimethylchromene): R_t_ = 12.582 min, MS (EI, 70 eV): m/z (%) = 190 (10) [M+], 175 (100) [M^+^-Me], 160 (10), 132 (14), 115 (4), 103 (4), 91 (4), 77 (8), 63 (6), 51 (8).

The ^1^HNMR (CDCl_3_) spectral data for compound **11a**: 1.41 (s, 6H, 2CH_3_), 3.75 (s, 3H, OCH_3_), 5.45 (d, 1H, J = 10 Hz, H-3), 6.26 (d, 1H, J = 10 Hz, H-4), 6.36 (m, 1H, ArH-8), 6.4 (d, 1H, J = 8.0 Hz, ArH-6), 6.86 (d, 1H, J = 8.0 Hz, ArH-5).

The ^13^CNMR (CDCl_3_) spectral data for compound **11a**: 28.02 (2CH_3_), 55.1 (OCH_3_), 76.7 (C-2), 102.09 (C-8), 106.8 (C-4a), 115.03 (C-5), 122.02 (C-4), 127.3 (C-3), 128.5 (C-6), 154.5 (C-8a), 161.06 (C-7).

The GC-MS spectral data for compound **11b** (7-O-ethyl-2,2-dimethylchromene): R_t_ = 13.432 min, MS (EI, 70 eV): m/z (%) = 204 (20) [M+], 161 (100) [M^+^-C_2_H_4_O], 189 (92), 132 (11), 115 (9), 105 (7), 91 (7), 77 (21), 63 (7), 51 (8).

The ^1^HNMR (CDCl_3_) spectral data for compound **11b**: 1.37 (t, 3H, J = 6.0 Hz, OCH_2_CH_3_), 1.42 (s, 6H, 2CH_3_), 4.01 (q, 2H, J = 6.0 Hz, OCH_2_CH_3_), 5.45 (d, 1H, J = 10 Hz, H-3), 6.26 (d, 1H, J = 10 Hz, H-4), 6.36 (m, 1H, ArH-8), 6.4 (d, 1H, J = 8.0 Hz, ArH-6), 6.86 (d, 1H, J = 8.0 Hz, ArH-5).

The ^13^CNMR (CDCl_3_) spectral data for compound **11b**: 15.2 (OCH_2_CH_3_), 28.4 (2CH_3_), 63.8 (OCH_2_CH_3_), 76.7 (C-2), 102.9 (C-8), 107.6 (C-4a), 114.9 (C-5), 122.3 (C-4), 127.3 (C-3), 128.1 (C-6), 154.5 (C-8a), 160.4 (C-7).

#### 3.2.6. Fungitoxic Evaluation of Synthetic Chromene and Chromanone Compounds

The antifungal activity of all synthesized compounds and the standard antifungal drug amphotericin-B was evaluated in vitro against two phytopathogenic fungi, *R. solani* and *A. niger*, using the poisoned food technique [69]. Pure fungi cultures were obtained from the Department of Plant Pathology, Faculty of Agriculture, Ain-Shams University, Egypt, and the Department of Biology, Faculty of Science, Memorial University of Newfoundland, St. John’s, NL. Canada. Two cultural media, Czapek–Dox agar (CDA) and potato dextrose agar (PDA), were used in this study. Cultural media were obtained from Merck Chem. Co (Canada). CDA media were used as specific growth media for *A. niger*, while PDA media were used as growth media for *R. solani*. The fungitoxic activity of 29 compounds (chromanones and chromenes) was tested against *A. niger* and *R. solani* in vitro at various concentrations, ranging from 100 to 800 μg × mL^−1^, while amphotericin-B (X-GEN, NY, USA) was used at 5–25 μg × mL^−1^. An in vitro assay was performed on PDA and CDA growth media treated with gradient concentrations of all the synthesized compounds (100–800 µg × mL^−1^ in sterilized DMSO (5%) + Tween-20 as a dispersing agent). Then, 1 mL of a solution containing different compounds was poured into sterilized melted media, homogenized, and plated into Petri dishes (90 × 15 mm). The media-containing compounds were incubated for 48 h at 25 °C. After incubation, all plates were inoculated with agar plugs containing fungi and incubated again at 22 °C for 8 days. To assess mycelial inhibition, fungal growth diameters (mm) were measured daily, and radial inhibition was calculated when negative control plates were fully covered with the fungal mycelial. For all treatments, four replicates were used, and the percentage of mycelial growth inhibition was calculated according to the equation suggested by Pandey et al. [70].

% mycelial inhibition = (dc − dt)/dc × 100(1)

where:

- dc = the diameter of the fungal colony in the negative control;
- dt = the diameter of the fungal colony in treatment.

The positive control (inoculated media with fungus and DMSO + Tween-20) was used to evaluate the toxicity of the solvent and the dispersing agent. In addition, the synthetic antibiotic drug, amphotericin-B (X-GEN, NY, USA), was also used as a standard antifungal drug at concentrations of 5–25 µg × mL^−1^. The EC_50_ and EC_90_ for all treatments were calculated using a regression equation between the log concentrations and the probit of the percentage growth inhibition of fungi, according to Abd El-Naeem et al. [71].

### 3.3. Molecular Docking

To determine the interaction mode of *A. niger*- and *R. solani*-encoded PGUs with different synthetic chromenes and chromanones, two available structures of *A. niger*-encoded PGUs (PDB ID 1CZF and 1NHC) were retrieved from the Protein Data Bank (PDB; https://www.rcsb.org/structure/) (accessed on 8 June 2022). Two additional PGU sequences (accession numbers KP896518 and KP896519) were retrieved from the NCBI Data Bank (https://pubmed.ncbi.nlm.nih.gov/) (downloaded on 7 June 2022) and their 3D structures were inferred using iTASSER (https://zhanggroup.org/I-TASSER/) (accessed on 10–15 June 2022) and Alpha fold2 (https://alphafold.ebi.ac.uk/) (accessed on 10–16 June 2022) tools. *Fusarium solani*-encoded VDAC mRNA sequences (accession # XM0462760) were retrieved from the NCBI Data Bank (https://pubmed.ncbi.nlm.nih.gov/) (accessed on 16 June 2022) and its 3D structure was inferred using the iTASSER (https://zhanggroup.org/I-TASSER/) (accessed on 16 June 2022) webserver. Additionally, a mouse-encoded VDAC (PDB id 3EMN) was retrieved to compare and validate the interactions.

To prepare protein input files, all water molecules, ligands, and ions were removed, and polar hydrogens were added from the PDB file using AutoDock Vina (version 1.10) [72]. Finally, the files were saved in the pdb format for docking processes.

Three-dimensional structures of different chromenes and chromanones were either downloaded from the Webchem webpage (https://pubchem.ncbi.nlm.nih.gov/compound) (accessed on 16 June 2022) or drawn using ChemBiochem Drew Ultra (version 12). All the ligands were used in the structure data file (sdf) format.

Blind molecular docking was performed to investigate the putative binding sites of chromenes and chromanones to PGUs and VDACs. The CB-dock2 server was used for blind docking with its default settings. Docking validation was accomplished by re-docking the original ligand into the receptor’s active site and compared the binding sites. For each used ligand, the CB-dock2 was set to generate ten cavities for docking [73]. Following docking, the ligand with the lowest Vina score was considered credible and photographed.

## 4. Conclusions

In conclusion, the chemical synthesis of naturally occurring precocenes I, II, and III in addition to their analogous, was performed by the reaction of corresponding di and trihydroxybenzenes with α,β-unsaturated carboxylic acid under mild conditions from POCl_3_ and ZnCl_2_ or AlCl_3_. The benzopyrane ring closure produced the key intermediate dihydroxy-2,2-dimethyl-chroman-4-ones **4a**–**c** or monohydroxy-2,2-dimethyl-chroman-4-ones (compound **9**). The selective O-alkylation on the benzene ring produced stepwise mono and di-*O*-alkoxy derivatives of chromanones **5a**–**f**, **6a**–**f**, and **10a**–**b**. The following reduction step by NaBH_4_ and in situ dehydration produced the corresponding 2*H*-1-chromene compounds **7a**–**f** and **11a**–**b**. The poison feed protocol was used to evaluate the antifungal activity of two categories of compounds (i) chroman-4-one with mono and di-*O*-alkoxy analogues and (ii) 2*H*-1-chromenes against *A. niger* and *R. solani*. The tested compounds showed different antifungal attribution to changes in the type and position of the substituent in both categories of compounds. These results were supported by molecular docking. The synthesized chromene and chromanone compounds are eco-friendly compounds because they are not organo-metalic, organophosphoures, or halogenated compounds which could be harmful to the environment. More studies are required to evaluate the effect of chromenes and chromanones in both field experiments with economically important plant pathogenic fungi on the molecular level.

## Figures and Tables

**Table 1:** Antifungal activity of different concentrations of synthesized compounds (**3a**–**b**, **4a**–**c**, **5a**–**f**, **6a**–**f**, **7a**–**f**, **8**, **10a**–**b**, and **11a**–**b**) against *Aspergillus niger* on Czapek Dox agar (CDA) media, EC_50_, and EC_90_ after probit analyses.

Compound	EC_50_ (µg × mL^−1^)	EC_90_ (µg × mL^−1^)	Reg. Equation	(R^2^)
Amphotericin-B	5.519	16.99	Log y = 3.4857x + 30.762	0.9971
**3a**	614.56	833.66	Log y = 9.6659x − 21.954	0.9575
**3b**	1023.29	1591.74	Log y = 6.6711x − 15.08	0.9454
**4a**	1664.00	5620.50	Log y = 2.4214x − 2.7997	0.9464
**4b**	1475.63	6939.33	Log y = 1.9038x − 1.0331	0.9377
**4c**	603.55	19,561.23	Log y = 0.8473x + 2.6439	0.9771
**5a**	3033.37	45,951.72	Log y = 1.0844x + 1.2242	0.9196
**5b**	630.80	6361.74	Log y = 1.2753x + 1.4293	0.9646
**5c**	125.70	5064.54	Log y = 0.7974x + 3.326	0.8440
**5d**	624.90	1628.46	Log y = 3.0345x − 3.4838	0.9931
**5e**	318.10	1274.00	Log y = 2.1241x − 0.3157	0.9992
**5f**	681.08	7654.24	Log y = 1.2188x + 1.5463	0.8207
**6a**	527.48	1434.27	Log y = 2.9464x − 3.0207	0.9395
**6b**	1191.3	10,743.32	Log y = 1.2878x + 1.0387	0.7784
**6c**	398.32	1380.38	Log y = 2.3377x − 1.0733	0.9229
**6d**	410.60	1269.20	Log y = 2.6116x − 1.8252	0.9499
**6e**	582.13	3269.80	Log y = 1.7078x + 0.2779	0.9578
**6f**	617.90	2392.70	Log y = 2.1769x − 1.0755	0.9350
**7a**	97.18	688.60	Log y = 1.5051x + 2.0085	0.9859
**7b** (Precocene II)	106.80	809.01	Log y = 1.4558x + 2.0466	0.7307
**7c**	8398.20	*	Log y = 0.2704x + 3.9389	0.9999
**7d**	170.58	197,513.13	Log y = 0.4178x + 4.0675	0.9995
**7e** (Precocene III)	3963.08	**	Log y = 0.4881x + 3.2438	0.9842
**7f**	4079.89	9371.57	Log y = 3.5441x − 7.7965	0.2556
**8**	356.98	969.71	Log y = 2.9493x − 2.5285	0.9175
**9**	1375.35	6760.83	Log y = 1.8546x − 0.8205	0.9955
**10a**	221.31	799.7	Log y = 2.2942x − 0.3799	0.9628
**10b**	2.91×10^8^	***	Log y = −0.0683x + 5.5781	0.0079
**11a** (Precocene I)	584.58	2060.81	Log y = 2.3392x − 1.4722	0.9945
**11b**	1235.61	5613.94	Log y = 1.9471x − 1.0202	0.8709

All data were corrected to the positive control. * The compounds with structure resemblance were grouped for comparison. * From Reg. Equation, Log C at 90% inhibition was 8.66 and EC_90_ = 4.5 × 108 µg × mL^−1^. ** From Reg. Equation, Log C at 90% inhibition was 6.2 and EC_90_ = 1.66 × 106 µg × mL^−1^. *** The EC_90_ of compound **10b** could not be calculated from the given Reg. Equation.

**Table 2:** Antifungal activity of the selected compounds against *Rhizoctonia solani* on PDA, EC_50_, and EC_90_ after probit analysis.

	EC_50_ (µg × mL^−1^)	EC_90_ (µg × mL^−1^)	Reg. Equation	(R2)
Amphotericin-B	2.70	10.86	Log y = 4.9381x + 36.331	0.9920
**3a**	123.77	544.38	Log y = 1.9898x + 0.8361	0.8622
**8**	76.62	282.90	Log y = 2.2562x + 0.7485	0.9878
**5b**	616.60	1059.53	Log y = 5.5307x − 10.451	0.9566
**5c**	46.88	146.16	Log y = 2.5918x + 0.6692	0.9432
**5d**	558.10	1424.47	Log y = 3.1454x − 3.6395	0.9809
**5e**	318.19	1244.68	Log y = 2.1608x − 0.4078	0.8465
**5f**	334.29	869.98	Log y = 3.0815x − 2.7781	0.9826
**6a**	593.10	928.85	Log y = 6.5702x − 13.22	0.9841
**6c**	381.13	765.89	Log y = 4.2232x − 5.9004	0.9857
**6d**	245.42	406.91	Log y = 5.8289x − 8.9305	0.9802
**6e**	645.06	1654.44	Log y = 3.1292x − 3.7918	0.9052
**6f**	204.55	720.24	Log y = 2.3414x − 0.4105	0.9951
**10a**	100.00	191.90	Log y = 4.5264x − 4.0541	0.989
**10b**	59.85	230.81	Log y = 2.1836x + 1.1196	0.991
**7a**	35.30	199.94	Log y = 1.6996x + 2.3694	0.9644
**7b**	4.94	74.69	Log y = 1.0857x + 4.2462	0.8489
**7f**	517.90	21,160.64	Log y = 0.7944x + 2.8438	0.991
**11a**	10.39	2057.29	Log y = 0.5573x + 4.4335	0.873
**11b**	116.47	3530.73	Log y = 0.8639x + 3.215	0.9432

All data were corrected to the positive control.

**Table 3:** The reaction time and % of reaction yield of the synthesis of monoalkoxy, monohydroxy 2,2-dimethylchroman-4-ones **(5a**–**f)**, dialkoxy, 2,2-dimethylchroman-4-ones (**6a**–**f**), and monoalkoxy, 2,2-dimethylchroman-4-ones **(10a**–**b)**.

Compound	5a	5b	5c	5d	5e	5f	6a	6b	6c	6d	6e	6f	10a	10b
Reaction Time (h)	6	3	8	6	2	5	10	15	40	8	8	60	6	10
* % of Reaction Yield	55.3	64.0	50.5	43.4	63.2	70.0	56.5	77.9	50.5	76.9	56.5	73.5	83.3	85.0

* Reaction yield= [actual yield (of purified compound)/theoretical yield] × 100.

**Table 4:** Reaction times required for reduction and dehydration of corresponding compounds (**6a**–**f**) and (**11a**–**b**), as well as the % reaction yields.

Compound	Reduction Time (h)	Dehydration Time	% Reaction Yield *
**7a**	2.0	2.0	58.4
**7b** (Precocene II)	1.5	1.5	87.3
**7c**	2.5	2.5	72.0
**7d**	1.5	3.0	80.0
**7e** (Precocene III)	2.0	2.0	62.5
**7f**	2.0	3.0	83.9
**11a** (Precocene I)	3.0	3.0	53.0
**11b**	4.0	3.0	61.6

* The reaction yield was calculated over the actual yield of the final step.

## Data Availability

All the data related to this study are mentioned in the manuscript and in the Supplementary Data.

## Supplementary Materials

The following supporting information can be downloaded at: https://www.mdpi.com/article/10.3390/molecules27217177/s1, Supporting Information file: NMR and MS Spectra a molecular docking table of the compounds. Table S1: Precocene and its derivatives interaction with polygalactouronases (PGU) encoded by A. niger and R. solani; and interaction with Volatge dependent anion channel (VDAC) encoded by mouse and F. solani; Figure S1: Mycelial growth inhibition of fungus A. niger by using different concentrations of **7a** and **7b**; Figure S2: Mycelial growth inhibition of fungus R. solani by using different concentrations of compounds **7b**, **10a**, **10b**, and **11a**; Figure S3: Mass spectrum of 1-(2′,3′,4′-trihydroxyphenyl)-3-methyl-1- oxo, buta-2-ene (**3a**); Figure S4: Proton chemical shift spectrum of 1-(2′,3′,4′-trihydroxyphenyl)-3-methyl-1- oxo, buta-2-ene (**3a**). (DMSO-d6, 500 MHz); Figure S5: Proton chemical shift spectrum of 1-(2′,4′,5′-trihydroxyphenyl)-3-methyl-1- oxo, buta-2-ene (**3b**). (DMSO-d6, 500 MHz); Figure S6: Mass spectrum of 7,8-dihydroxy 2,2-dimethyl chroman-4-one (**4a**); Figure S7 Proton chemical shift spectrum of 7,8-dihydroxy 2,2-dimethyl chroman-4-one (**4a**). (DMSO-d6, 500 MHz); Figure S8: Proton chemical shift spectrum 6,7-dihydroxy 2,2-dimethyl chroman-4-one (**4b**) (DMSO-d6, 500 MHz); Figure S9: Mass spectrum of 5,7-dihydroxy 2,2-dimethyl chroman-4-one (**4c**); Figure S10: Proton chemical shift spectrum of 5,7-dihydroxy 2,2-dimethyl chroman-4-one (**4c**). (DMSO-d6, 500 MHz); Figure S11: Mass spectrum of 7-methoxy, 8-hydroxy 2,2-dimethyl chroman-4-one (**5a**); Figure S12: Proton chemical shift spectrum of 7-methoxy, 8-hydroxy 2,2-dimethyl chroman-4-one (**5a**). (CDCl3, 500 MHz); Figure S13: Mass spectrum of 7-methoxy, 6-hydroxy 2,2-dimethyl chroman-4-one (**5b**); Figure S14: Proton chemical shift spectrum of 7-methoxy, 6-hydroxy 2,2-dimethyl chroman-4-one (**5b**). (CDCl3, 500 MHz); Figure S15: Mass spectrum of 7-methoxy, 5-hydroxy 2,2-dimethyl chroman-4-one (**5c**); Figure S16: Proton chemical shift spectrum of 7-methoxy, 5-hydroxy 2,2-dimethyl chroman-4-one (**5c**). (CDCl3, 500 MHz); Figure S17: Mass spectrum of 7-ethoxy, 8-hydroxy 2,2-dimethyl chroman-4-one (5d); Figure S18: Proton chemical shift spectrum of 7-ethoxy, 8-hydroxy 2,2-dimethyl chroman-4-one (**5d**). (CDCl3, 500 MHz); Figure S19: Mass spectrum of 7-ethoxy, 6-hydroxy 2,2-dimethyl chroman-4-one (**5e**); Figure S20: Proton chemical shift spectrum of 7-ethoxy, 6-hydroxy 2,2-dimethyl chroman-4-one (**5e**). (CDCl3, 500 MHz); Figure S21: Mass spectrum of 7-ethoxy, 5-hydroxy 2,2-dimethyl chroman-4-one (**5f**); Figure S22: Proton chemical shift spectrum of 7-ethoxy, 5-hydroxy 2,2-dimethyl chroman-4-one (**5f**). (CDCl3, 500 MHz); Figure S23: Mass spectrum of 7,8-dimethoxy, 2,2-dimethyl chroman-4-one (**6a**); Figure S24: Proton chemical shift spectrum of 7,8-dimethoxy, 2,2-dimethyl chroman-4-one (**6a**). (CDCl3, 500 MHz); Figure S25: Mass spectrum of 6,7-dimethoxy, 2,2-dimethyl chroman-4-one (6b); Figure S26: Proton chemical shift spectrum of 6,7-dimethoxy, 2,2-dimethyl chroman-4-one (**6b**). (CDCl3, 500 MHz); Figure S27: Mass spectrum of 5,7-dimethoxy 2, 2-dimethyl chroman-4-one (**6c**). (CDCl3, 500 MHz); Figure S28: Proton chemical shift spectrum of 5,7-dimethoxy 2,2-dimethyl chroman-4-one (**6c**). (CDCl3, 500 MHz); Figure S29: Mass spectrum of 7-ethoxy,8-mehoxy 2, 2-dimethyl chroman-4-one (**6d**); Figure S30: Proton chemical shift spectrum of 7-ethoxy,8-mehoxy 2, 2-dimethyl chroman-4-one (**6d**). (CDCl3, 500 MHz); Figure S31: Mass spectrum of 7-ethoxy,6-mehoxy 2, 2-dimethyl chroman-4-one (**6e**); Figure S32: Proton chemical shift spectrum of 7-ethoxy,6-mehoxy 2, 2-dimethyl chroman-4-one (**6e**). (CDCl3, 500 MHz); Figure S33: Mass spectrum of 7-ethoxy,5-mehoxy 2, 2-dimethyl chroman-4-one (**6f**); Figure S34: Proton chemical shift spectrum of 7-ethoxy,5-mehoxy 2, 2-dimethyl chroman-4-one (**6f**). (CDCl3, 500 MHz); Figure S35: Mass spectrum of 7,8-dimethoxy, 2,2-dimethyl 2H-1-chromene (**7a**); Figure S36: Proton chemical shift spectrum of 7,8-dimethoxy 2,2-dimethyl 2H-1-chromene (**7a**). (CDCl3, 500 MHz); Figure S37: Carbon chemical shift spectrum of 7,8-dimethoxy 2,2-dimethyl 2H-1-chromene (**7a**). (CDCl3, 500 MHz); Figure S38: Mass spectrum of 6,7-dimethoxy, 2,2-dimethyl 2H-1-chromene (**7b**) (Precocene II); Figure S39: Proton chemical shift spectrum of 6,7-dimethoxy 2,2-dimethyl 2H-1-chromene (**7b**) (Precocene II). (CDCl3, 500 MHz); Figure S40: Carbon chemical shift spectrum of 6,7-dimethoxy 2,2-dimethyl 2H-1-chromene (**7b**) (Precocene II). (CDCl3, 500 MHz); Figure S41: Mass spectrum of 5,7-dimethoxy, 2,2-dimethyl 2H-1-chromene (7c); Figure S42: Proton chemical shift spectrum of 5,7-dimethoxy 2,2-dimethyl 2H-1-chromene (**7c**) (CDCl3, 500 MHz); Figure S43: Mass spectrum of 7-ethoxy, 8-methoxy, 2,2-dimethyl 2H-1-chromene (**7d**); Figure S44: Proton chemical shift spectrum of 7-ethoxy, 8-methoxy 2,2-dimethyl 2H-1-chromene (**7d**). (CDCl3, 500 MHz); Figure S45: Carbon chemical shift spectrum of 7-ethoxy, 8-methoxy 2,2-dimethyl 2H-1-chromene (**7d**) (CDCl3, 500 MHz); Figure S46: Mass spectrum of 7-ethoxy, 6-methoxy, 2,2-dimethyl 2H-1-chromene (**7e**) (Precocene III); Figure S47: Proton chemical shift spectrum of 7-ethoxy, 6-methoxy, 2,2-dimethyl 2H-1-chromene (**7e**) (Precocene III) (CDCl3, 500 MHz); Figure S48: Carbon chemical shift spectrum of 7-ethoxy, 6-methoxy, 2,2-dimethyl 2H-1-chromene (**7e**) (Precocene III) (CDCl3, 500 MHz); Figure S49: Mass spectrum of 7-ethoxy, 5-methoxy, 2,2-dimethyl 2H-1-chromene (**7f**); Figure S50: Proton chemical shift spectrum of 7-ethoxy,5-methoxy, 2,2-dimethyl 2H-1-chromene (**7f**) (CDCl3, 500 MHz); Figure S51: Carbon chemical shift spectrum of 7-ethoxy,5-methoxy, 2,2-dimethyl 2H-1-chromene (**7f**) (CDCl3, 500 MHz); Figure S52: Mass spectrum of 1-(2′,4′-dihydroxyphenyl)-3-methyl-1- oxo-buta-2-ene (**8**); Figure S53: Proton chemical shift spectrum of 1-(2′,4′-dihydroxyphenyl)-3-methyl-1- oxo-buta-2-ene (**8**) (DMSO-d6, 500 MHz); Figure S54: Mass spectrum of 7-hydroxy 2,2-dimethyl chroman-4-one (**9**); Figure S55: Proton chemical shift spectrum of 7-hydroxy 2,2-dimethyl chroman- 4-one (**9**) (CDCl3, 500 MHz); Figure S56: Mass spectrum of 7-methoxy 2,2-dimethyl chroman-4-one (**10a**); Figure S57: Proton chemical shift spectrum of 7-methoxy 2,2-dimethyl chroman-4-one (**10a**) (CDCl3, 500 MHz); Figure S58: Mass spectrum of 7-ethoxy 2,2-dimethyl chroman-4-one (**10b**); Figure S59: Proton chemical shift spectrum of 7-ethoxy 2,2-dimethyl chroman-4-one (10b) (CDCl3, 500 MHz); Figure S60: Mass spectrum of 7-methoxy 2,2-dimethyl 2H-1-chromene (**11a**) (Precocene I); Figure S61: Proton chemical shift spectrum of 7-methoxy 2,2-dimethyl 2H-1-chromene (**11a**) (Precocene I) (CDCl3, 500 MHz); Figure S62: Carbon chemical shift spectrum of 7-methoxy 2,2-dimethyl 2H-1-chromene (**11a**) (Precocene I) (CDCl3, 500 MHz); Figure S63: Mass spectrum of 7-ethoxy 2,2-dimethyl 2H-1-chromene (**11b**); Figure S64: Proton chemical shift spectrum of 7-ethoxy 2,2-dimethyl 2H-1-chromene (**11b**) (CDCl3, 500 MHz); Figure S65: Carbon chemical shift spectrum of 7-ethoxy 2,2-dimethyl *2H*-1-chromene (**11b**) (CDCl3, 500 MHz).
