# Supplementary material for: The Synthesis, Fungicidal Activity, and in Silico Study of Alkoxy Analogues of Natural Precocenes I, II, and III
Source: Molecules. 2022 Oct 24;27(21):7177. doi: 10.3390/molecules27217177 (PMC9658145; doi:10.3390/molecules27217177)

Supplementary Data—

The Synthesis, Fungicidal Activity, and in Silico Study of Alkoxy Analogues of Natural Precocenes I, II, and III

Khaled M. A. Ramadan [1,2,*], Hossam S. El-Beltagi [3,4,*], Zafar Iqbal [1] and Eslam S. A. Bendary [2]

[1] Central Laboratories, King Faisal University, Al-Ahsa 31982, Saudi Arabia
[2] Biochemistry Department, Faculty of Agriculture, Ain Shams University, Cairo 11241, Egypt
[3] Agricultural Biotechnology Department, College of Agriculture and Food Sciences, King Faisal University, Al-Ahsa 31982, Saudi Arabia
[4] Biochemistry Department, Faculty of Agriculture, Cairo University, Giza 12613, Egypt
* Correspondence: kramadan@kfu.edu.sa (K.M.A.R.); helbeltagi@kfu.edu.sa (H.S.E.-B.)

Supplementary Table S1. Precocene and its derivatives interaction with polygalactouronases (PGU) encoded by *A. niger* and *R. solani*; and interaction with Volatge dependent anion channel (VDAC) encoded by mouse and *F. solani*

7-alkoxy 2,2-dimethyl 2H-1-chromene [(11 a; Precocene-I)]

Fungi	Protein	Ligand	Vina score	Cavity score	Ligand binding sites (AA number)
A. niger	PGU1_1CZF_pdb	Precocene 1	-5.8	246	127,150,153,155,180,183,184,185,202,205,207,
A. niger	PGU1_1NHC_pdb	Precocene 1	-6.4	126	227,228,229,256,257,258,305,307,308,336,337
R. solani	PGU1_KP896518_Alpha2	Precocene 1	-7.4	75	142,143,176,180,182,185,202,203,206,207,208,209,224,229,230,231,255,257,258,291
R. solani	PGU2_ KP896519_ Alpha2	Precocene 1	-5.8	178	130,166,167,168,201,231,233,236,260,277,283,308,314,349,350
R. solani	PGU1_KP896518_iTASSER	Precocene 1	-6.2	359	115,142,179,180,182,185,202,203,206,208,224,229,230,255,257,291,

R. solani	PGU2_ KP896519_ iTASSER	Precocene 1	-6.7	1417	115,118,121,125,126,128,129,130,134,166,168,178,196,199,201,231,232,233,236,254,266,283,314,

7-alkoxy 2,2-dimethyl 2H-1-chromene [(11 a; Precocene-I)]

Fungi	Protein	Ligand	Vina score	Cavity score	Ligand binding sites (AA number)
Mouse	mVDA_3emn	Precocene 1	-5.8	139	18,207,208,209,218,219,220,236,238,242
F. solani	FsVDAC_iT	Precocene 1	-6.3	3870	1,2,3,6,112,118,121,164,165,168,169

Dialkoxy 2,2-dimethyl *2H*-1-chromene [(7b; Precocene-II)]

Fungi	Protein	Ligand	Vina score	Cavity score	Ligand binding sites (AA number)
A. niger	PGU1_1CZF_pdb	Precocene II	-5.4	246	127,150,153,154,180,183,184,185,205,207, 229,233,
A. niger	PGU1_1NHC_pdb	Precocene II	-6.4	126	227,228,229,256,257,267,268,288,306,307,308,336,337,
R. solani	PGU1_KP896518_Alpha2	Precocene II	-6.0	172	35,38,39,41,59,60,61,62,83,84,85,86,
As above	As above	As above	-5.4	142	176,207,209,229,230,231,258,260,
R. solani	PGU2_ KP896519_ Alpha2	Precocene II	-6.2	1038	130,166,167,168,231,233,236,254,260,277,283,314,349,350
R. solani	PGU1_KP896518_iTASSER	Precocene II	-6.2	359	283,286,287,288,291,294,295,323,326,327,328,351,353,
R. solani	ExoPGU2_ KP896519_ iTASSER	Precocene II	-6.6	1417	112,115,118,121,125,126,129,132,134,176,178,198,200,201

Fungi	Protein	Ligand	Vina score	Cavity score	Ligand binding sites (AA number)
Mouse	mVDAC_3emn	Precocene II	-6.0	139	14,15,18,207,218,219,220,236,237238
F. solani	FsVDAC_iT	Precocene II	-6.3	3870	1,2,3,6,112,118,121,164,165,168,169

Dialkoxy 2,2-dimethyl *2H*-1-chromene [(7d; Precocene-II)]

Fungi	Protein	Ligand	Vina score	Cavity score	Ligand binding sites (AA number)
A. niger	PGU1_1CZF_pdb	7E8M22DM	-5.8	483	150,153,154,155,180,183,184,185,188,201,202,205,206,207,228,233
A. niger	PGU1_1NHC_pdb	7E8M22DM	-6.7	1901	206,227,228,229,257,258,259,268,287,288,306,307,308,309
R. solani	PGU1_KP896518_Alpha2	7E8M22DM	-5.3	432	196,219,241,270,276,274,276,300,301,304,306
As above	As above	7E8M22DM	-5.2	172	35,38,39,41,59,60,61,62,83,84,85,86,
R. solani	ExoPGU2_ KP896519_ Alpha2	7E8M22DM	-6.3	1038	130,166,167,168,201,231,232,233,254,260,277,283,316,349,350,351,
R. solani	PGU1_KP896518_iTASSER	7E8M22DM	-5.6	359	283,286,287,288,291,294,295,323,326,327,348,351,353,
R. solani	ExoPGU2_ KP896519_ iTASSER	7E8M22DM	-6.8	1417	118,121,125,126,128,173,176,178,196,198,199,200,201,232,254,

Fungi	Protein	Ligand	Vina score	Cavity score	Ligand binding sites (AA number)
Mouse	mVDAC_3emn	7E8M22DM	-5.8	139	14,15,18,19,207,220,236,238,242,262,
F. solani	VDAC_iT	7E8M22DM	-6.5	3839	23,24,25,46,48,50,333,335,394,395,396,397,399,401

7-methoxy-5-hydroxy-2,2-dimethylchroman-4-one (5c)

Fungi	Protein	Ligand	Vina score	Cavity score	Ligand binding sites (AA number)
A. niger	PGU1_1CZF_pdb	7M5H22DMC4one	-6	764	127,150,153,155,180,183,185,186,202,205,207,228,233
		7M5H22DMC4one	-5.9	567	178,180,201,202,205,207,223,226,228,233,252,256
A. niger	PGU1_1NHC_pdb	7M5H22DMC4one	-7.2	1901	227,228,256,257,258,259,267,268,288,305,306,307,308,337,
R. solani	PGU1_KP896518_Alpha2	7M5H22DMC4one	-5.8	432	136,137,144,167,196,198,201,219,220,223,225,246,274,276,309
As above	As above	7M5H22DMC4one	-5.8	172	35,38,39,41,59,60,61,62,83,84,85,86,
R. solani	ExoPGU2_ KP896519_ Alpha2	7M5H22DMC4one	-6.7	1038	130,166,167,168,201,231,233,254,255,258,260,277,312,314,319, 349,350,

R. solani	PGU1_KP896518_iTASSER	7M5H22DMC4one	-6.4	359	286,288,291,294,295,325,326,327,328,348,351,353,355
R. solani	ExoPGU2_ KP896519_ iTASSER	7M5H22DMC4one	-6.7	1417	115,118,121,125,126,128,129,134,173,176,178,196,198,200,201, 232,

Fungi	Protein	Ligand	Vina score	Cavity score	Ligand binding sites (AA number)
Mouse	mVDAC_3emn	7M5H22DMC4one	-6.2	933	7,8,10,11,15,154,168,181,183,185,189,190,191,193,209
Mouse	mVDAC_3emn	7M5H22DMC4one	-5.6	139	7,8,10,11,15,254,168,181,183,185,189,190,191,193,209
F. solani	FsVDAC_iT	7M5H22DMC4one	-7.0	3839	23,24,25,46,48,50,117,333,334,335,394,395,396,397,398,399

7-methoxy-2,2-dimethylchroman-4-one (10a)

Fungi	Protein	Ligand	Vina score	Cavity score	Ligand binding sites (AA number)
A. niger	PGU1_1CZF_pdb	7M5H22DMC4one	-5.6	764	127,150,153,155,180,183,185,186,183,202,205,228,233
		7M5H22DMC4one	-5.2	567	178,180,183,201,202,205,207,223,228,233,252,256,258,294,
A. niger	PGU1_1NHC_pdb	7M5H22DMC4one	-7.1	1901	228,229,256,257,258,267,268,287,288,287,306,307,308,336,337,
R. solani	PGU1_KP896518_Alpha2	7M5H22DMC4one	-5.5	432	137,167,196,198,201,219,223,225,226,241,247,270,274,276,309
R. solani	ExoPGU2_ KP896519_ Alpha2	7M5H22DMC4one	-6.0	1038	130,166,167,168,201,231,233,254,255,258,260,314,349,350
R. solani	PGU1_KP896518_iTASSER	7M5H22DMC4one	-7.0	359	283,286,288,291,294,295,326,327,328,348,351,353,355
R. solani	ExoPGU2_ KP896519_ iTASSER	7M5H22DMC4one	-6.8	1417	115,118,121,125,126,128,129,134,173,176,178,196,198,200,201, 230,232

Fungi	Protein	Ligand	Vina score	Cavity score	Ligand binding sites (AA number)

Mouse	mVDAC_3emn	7M22DMC4one	-5.9	933	6,94,96,116,117,118,119,124,125,126,127,128,139,141,
Mouse	mVDAC_3emn	7M22DMC4one	-5.6	139	15,18,207,219,220,236,237,238,242,262
F. solani	FsVDAC_iT	7M22DMC4one	-6.7	3839	23,24,25,48,50,117,333,334,335,394,395,396,397,399

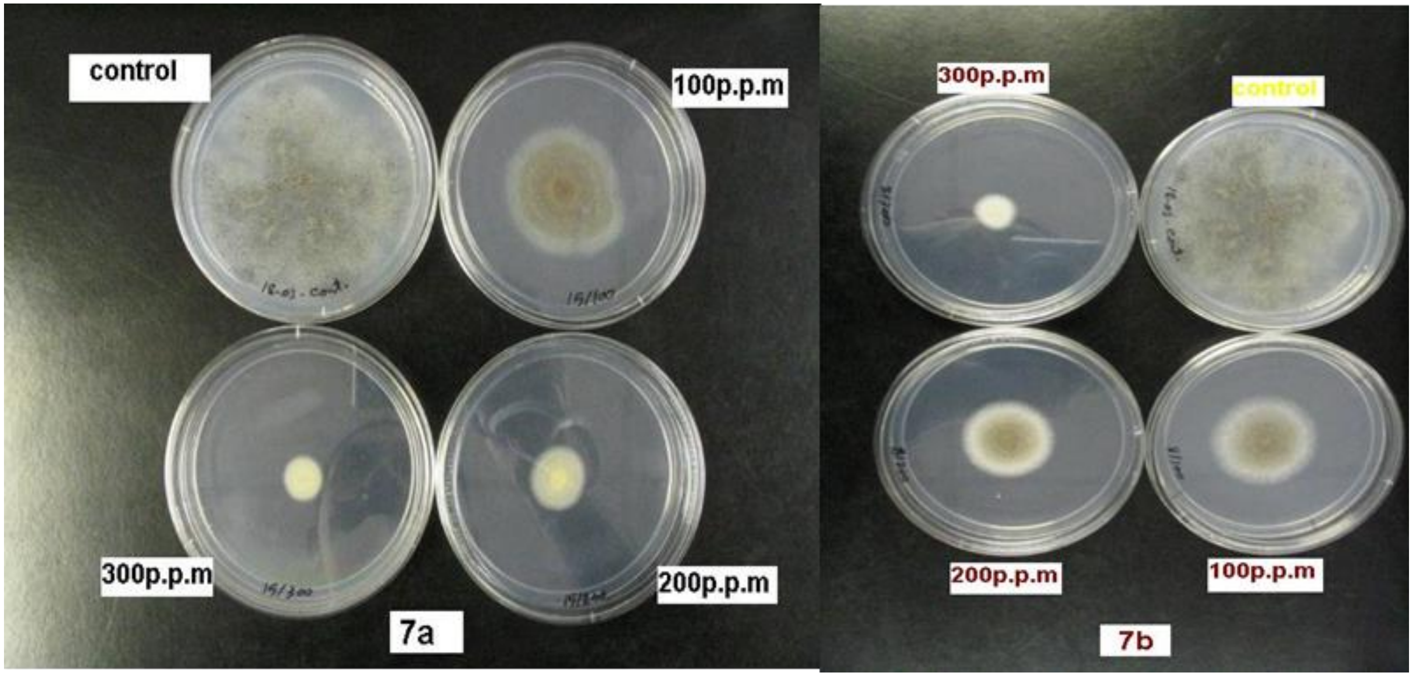

Supplementary Figure S1. Mycelial growth inhibition of fungus *A. niger* by using different concentrations of 7a and 7b.

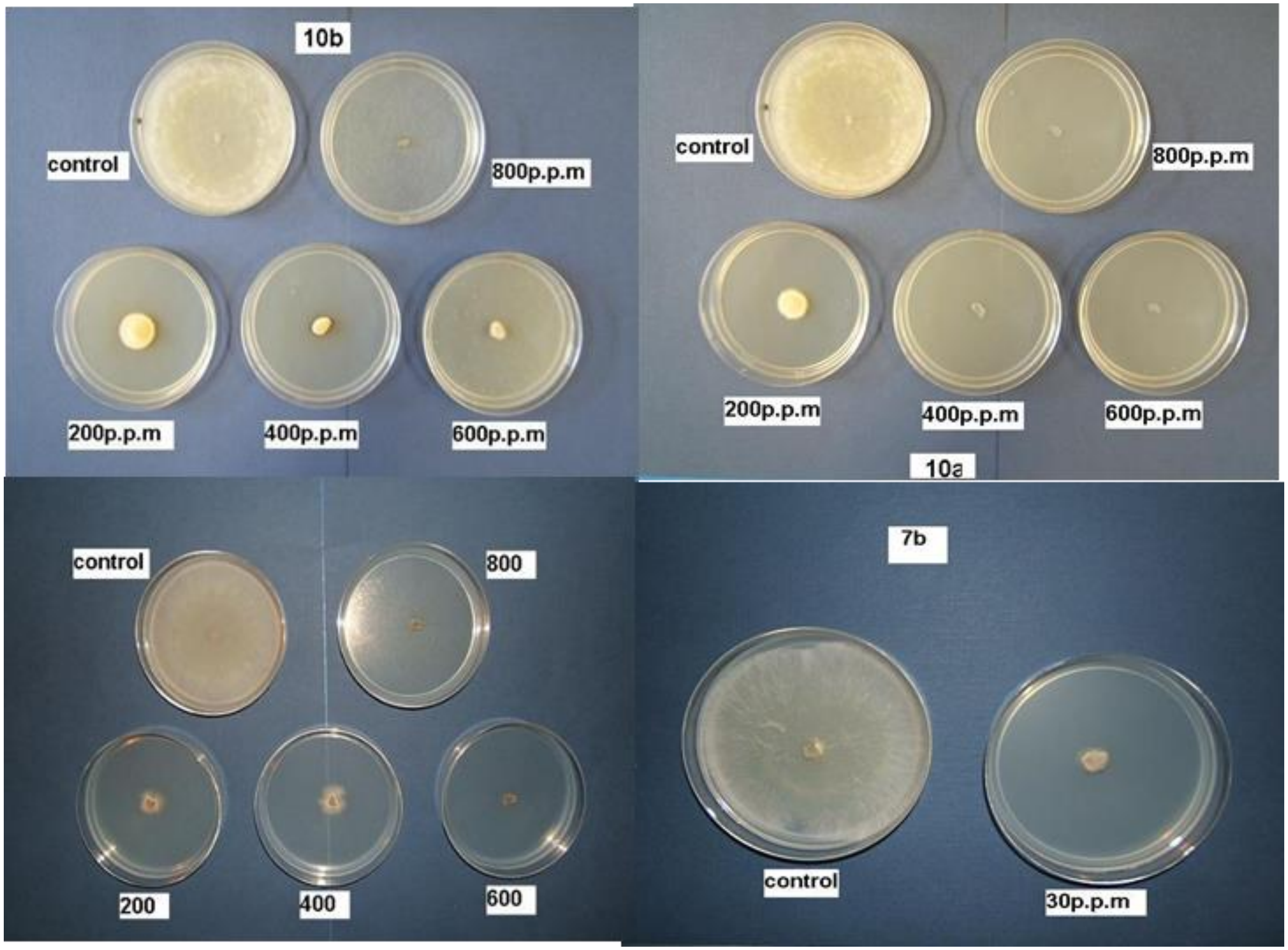

Supplementary Figure S2. Mycelial growth inhibition of fungus *R. solani* by using different concentrations of compounds 7b, 10a, 10b, and 11a.

Supplementary Figure S3.- Mass spectrum of 1-(2`,3`,4`-trihydroxyphenyl.-3-methyl-1- oxo,buta-2-ene (3a).

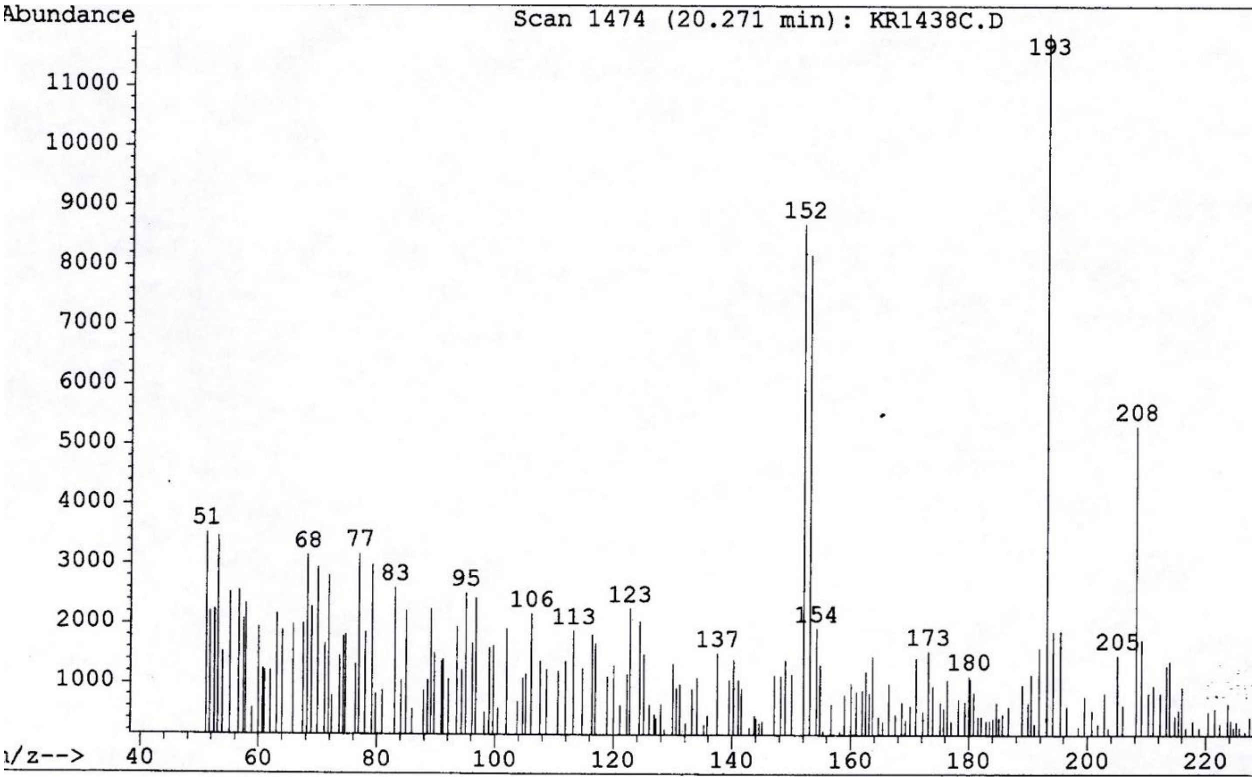

Supplementary Figure S4.- Proton chemical shift spectrum of 1-(2`,3`,4`-trihydroxyphenyl.-3-methyl-1- oxo, buta-2-ene (3a).

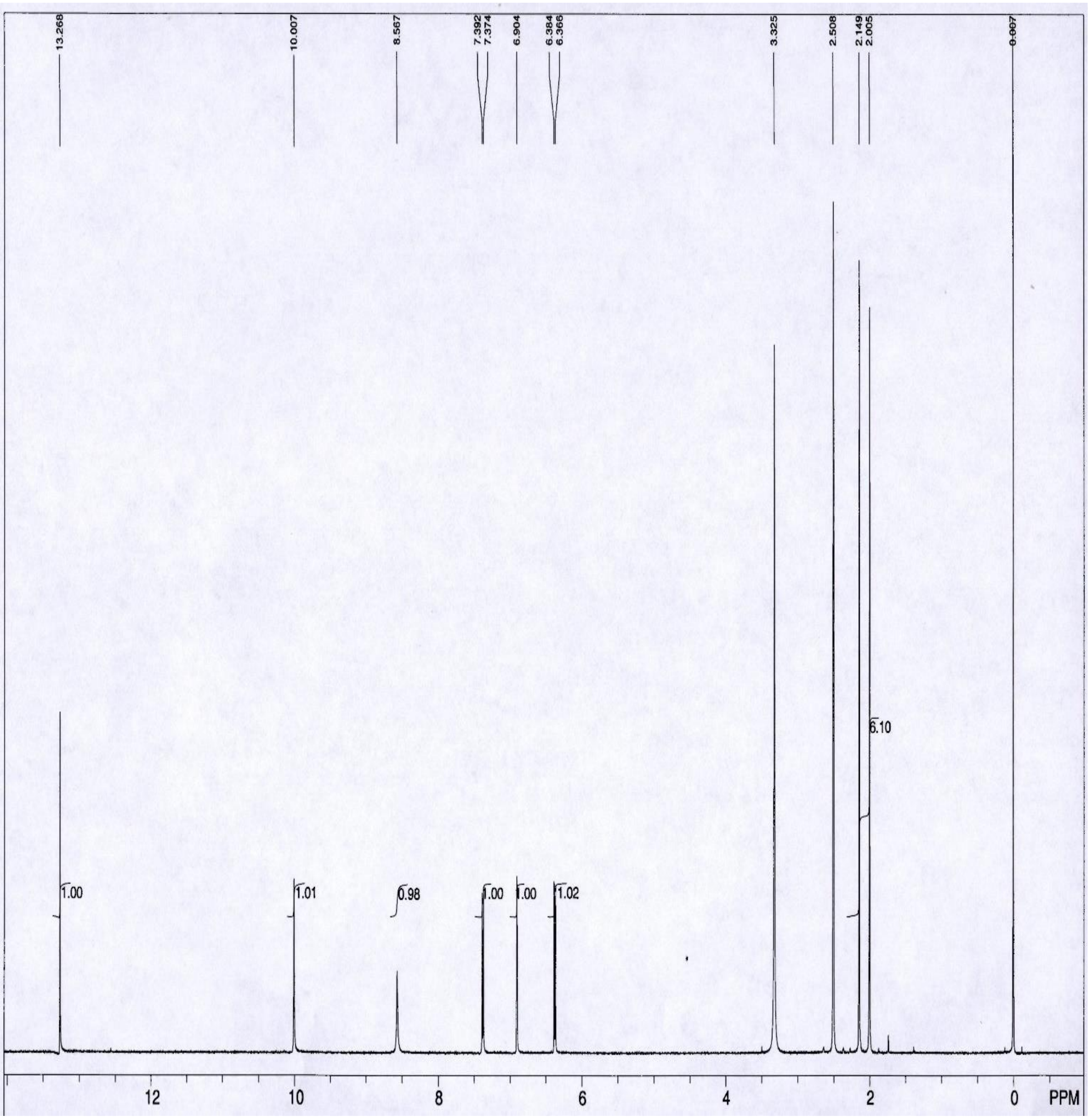

Supplementary Figure S5.- Proton chemical shift spectrum of 1-(2`,4`,5`-trihydroxyphenyl.-3-methyl-1- oxo, buta-2-ene (3b).

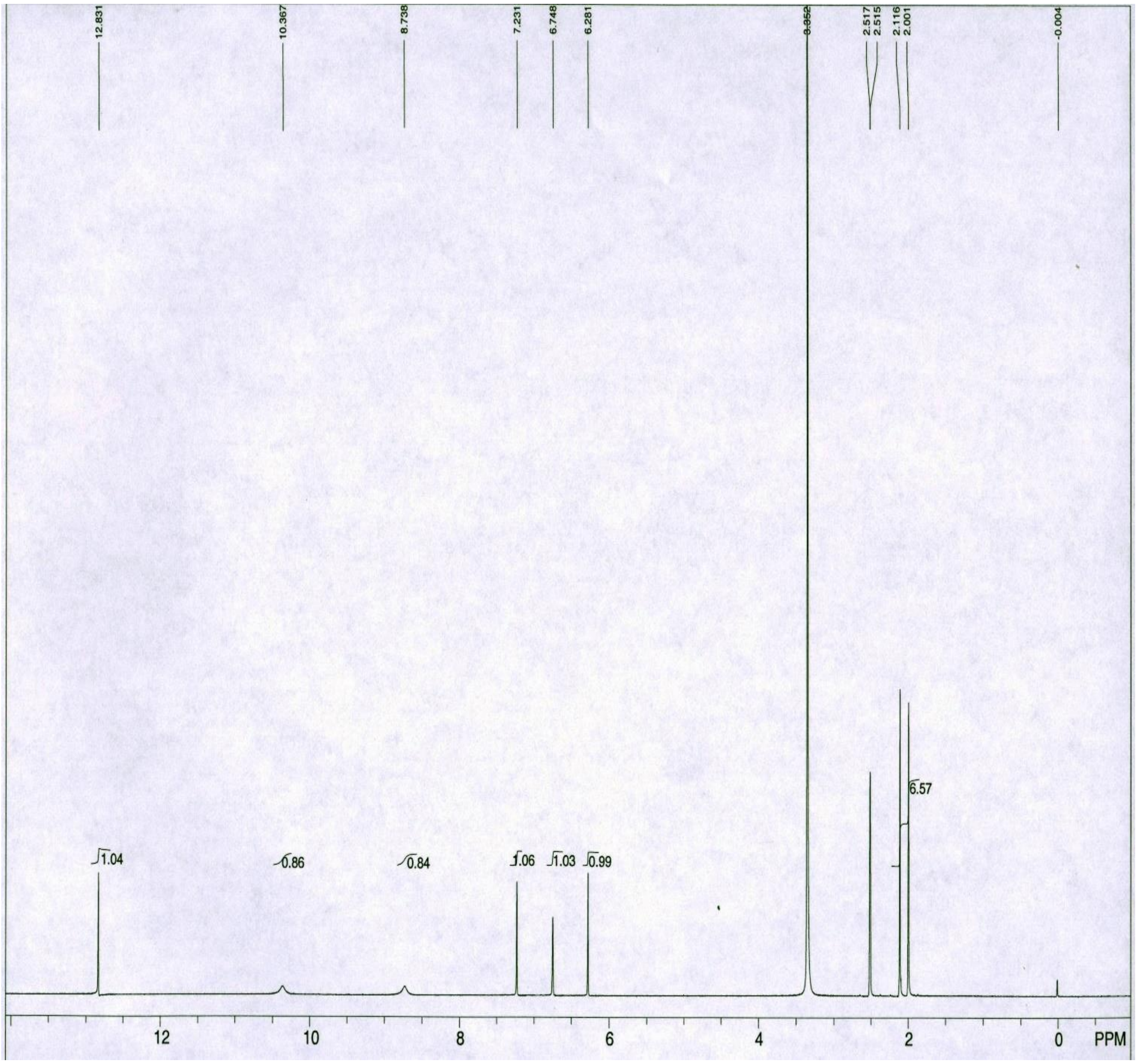

Supplementary Figure S6.- Mass spectrum of 7,8-dihydroxy 2,2-dimethyl chroman-4-one (4a).

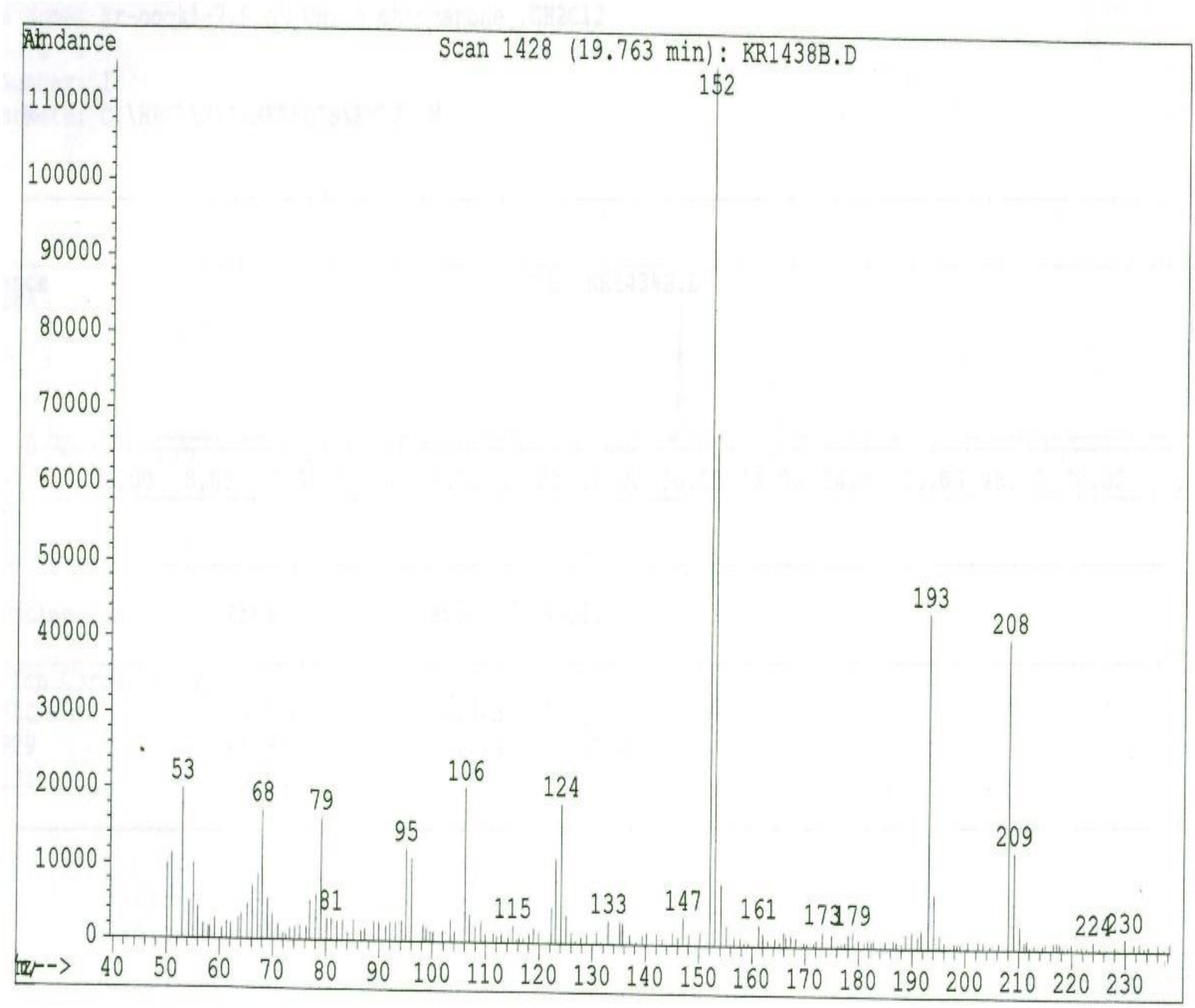

Supplementary Figure S7.- Proton chemical shift spectrum of 7,8-dihydroxy 2,2-dimethyl chroman-4-one (4a).

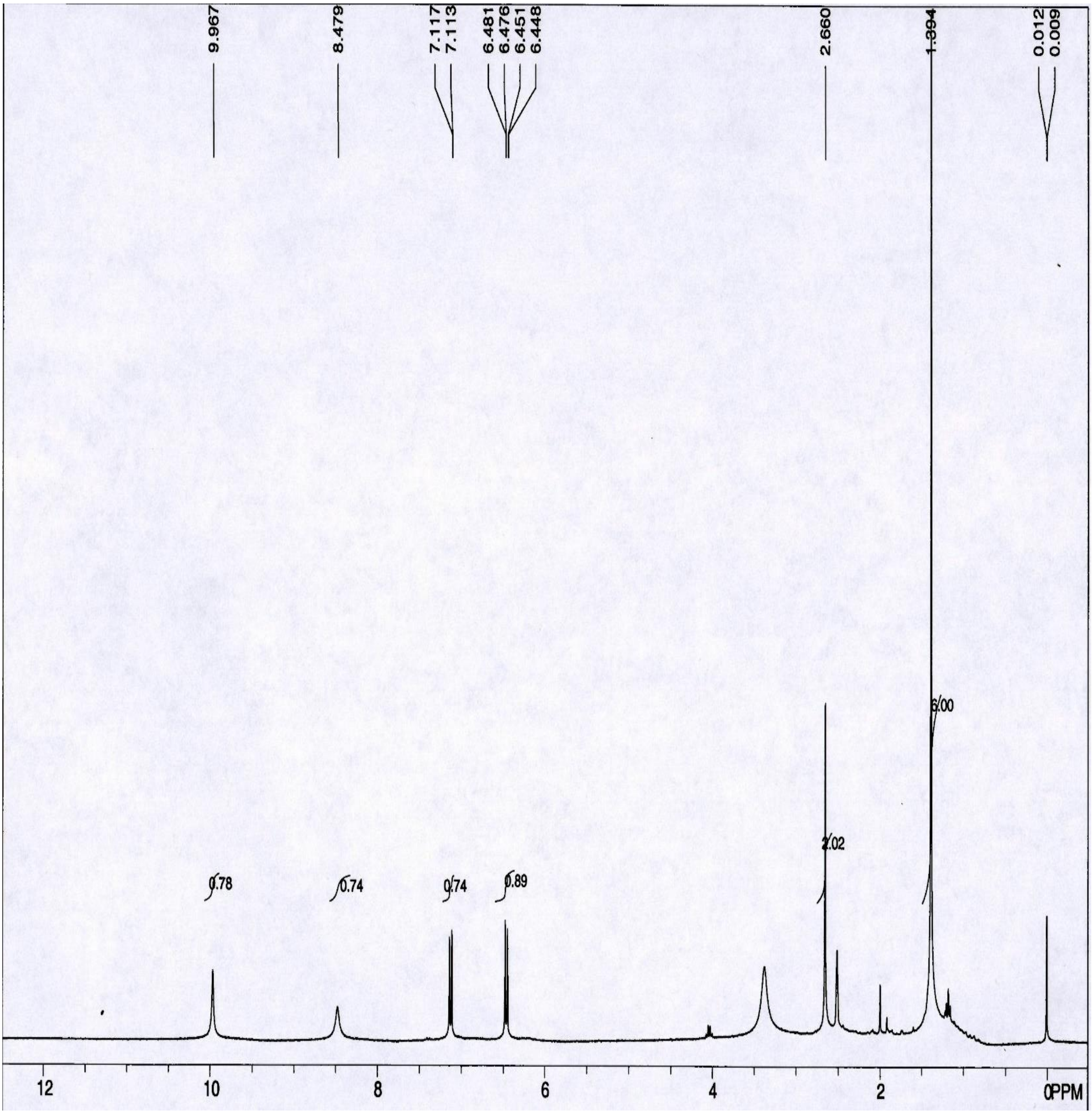

Supplementary Figure S8.- Proton chemical shift spectrum 6,7-dihydroxy 2,2-dimethyl chroman-4-one (4b).

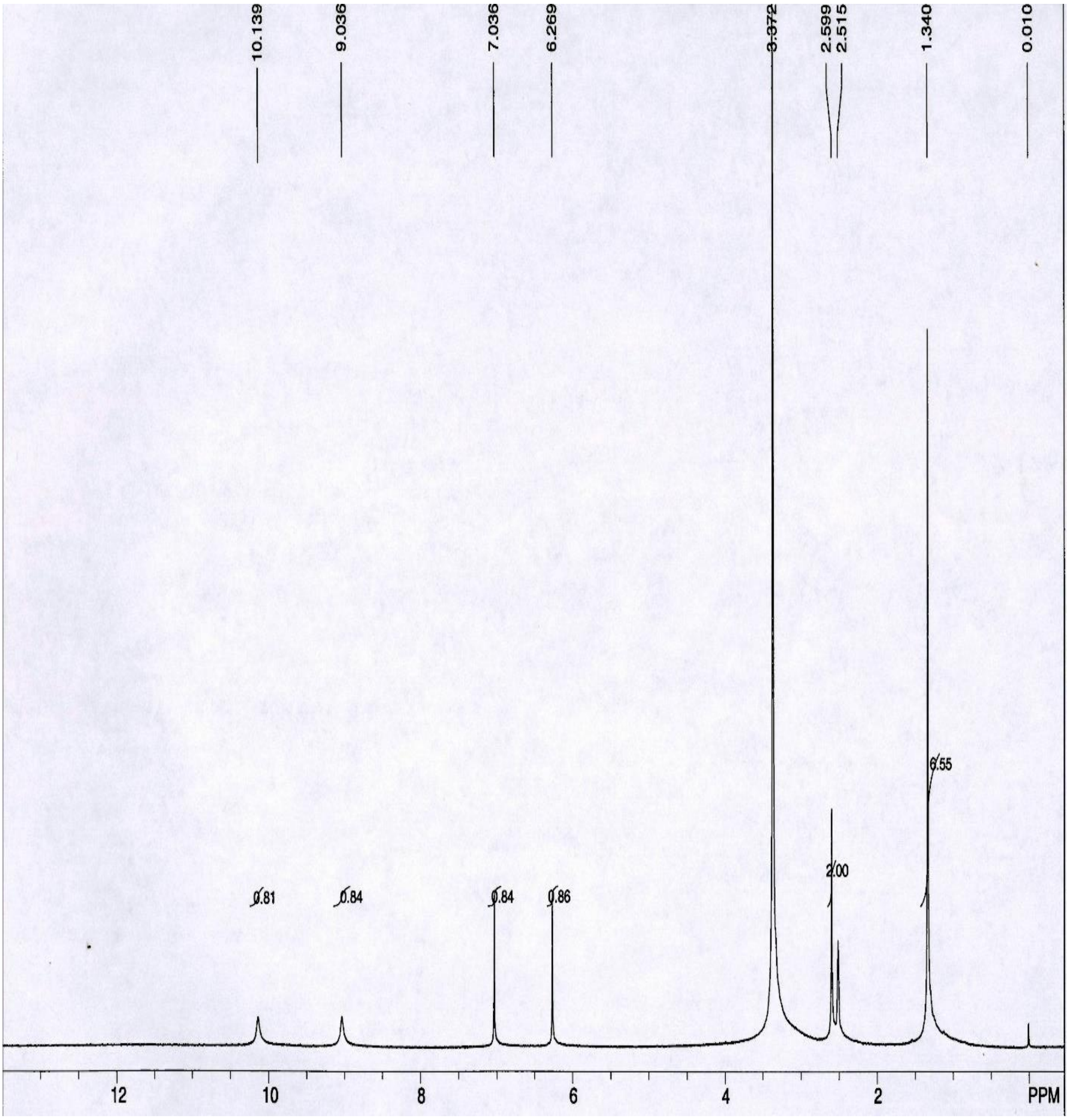

Supplementary Figure S9.- Mass spectrum of 5,7-dihydroxy 2,2-dimethyl chroman-4-one (4c).

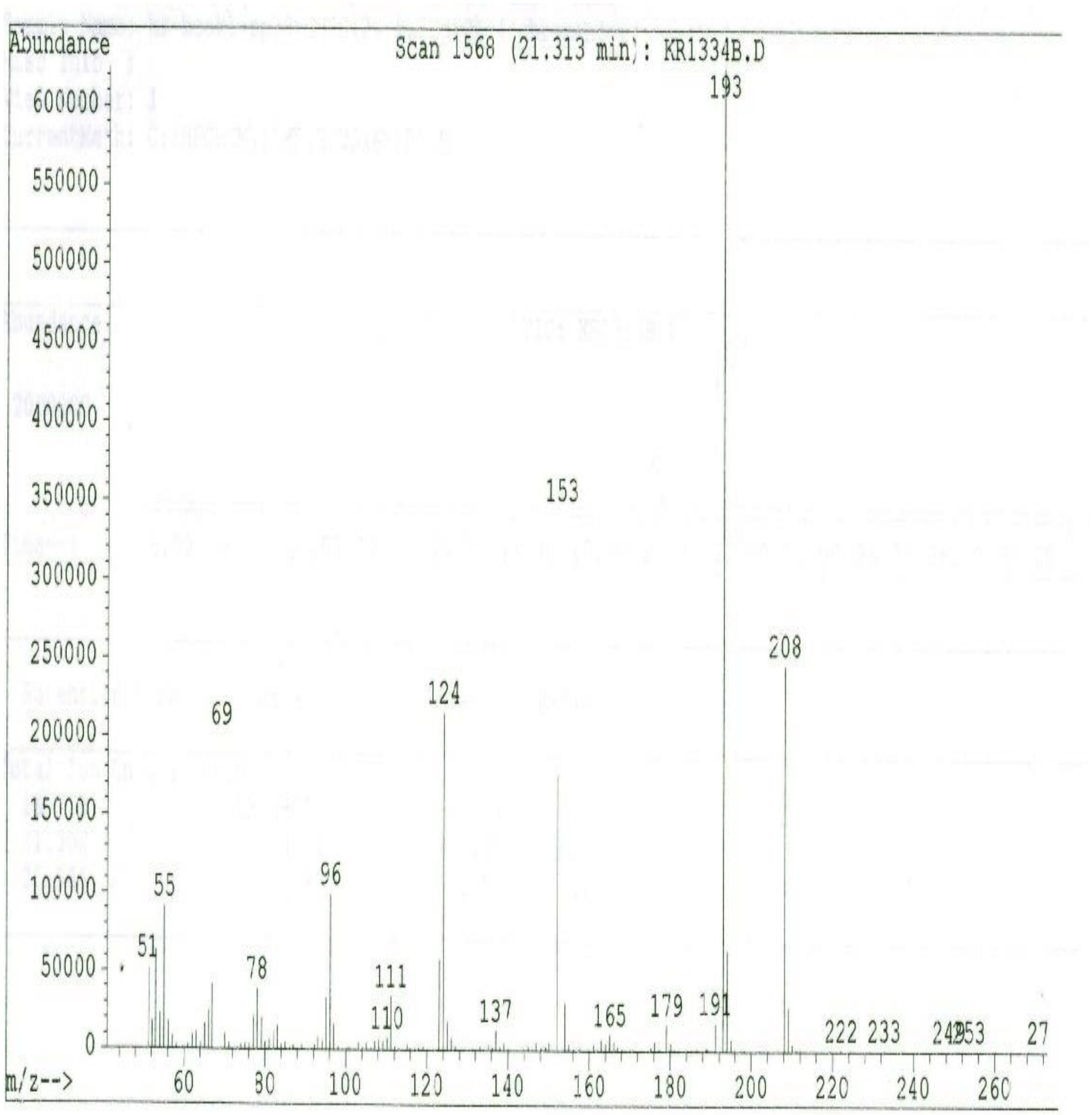

Supplementary Figure S10.- Proton chemical shift spectrum of 5,7-dihydroxy 2,2-dimethyl chroman-4-one (4c).

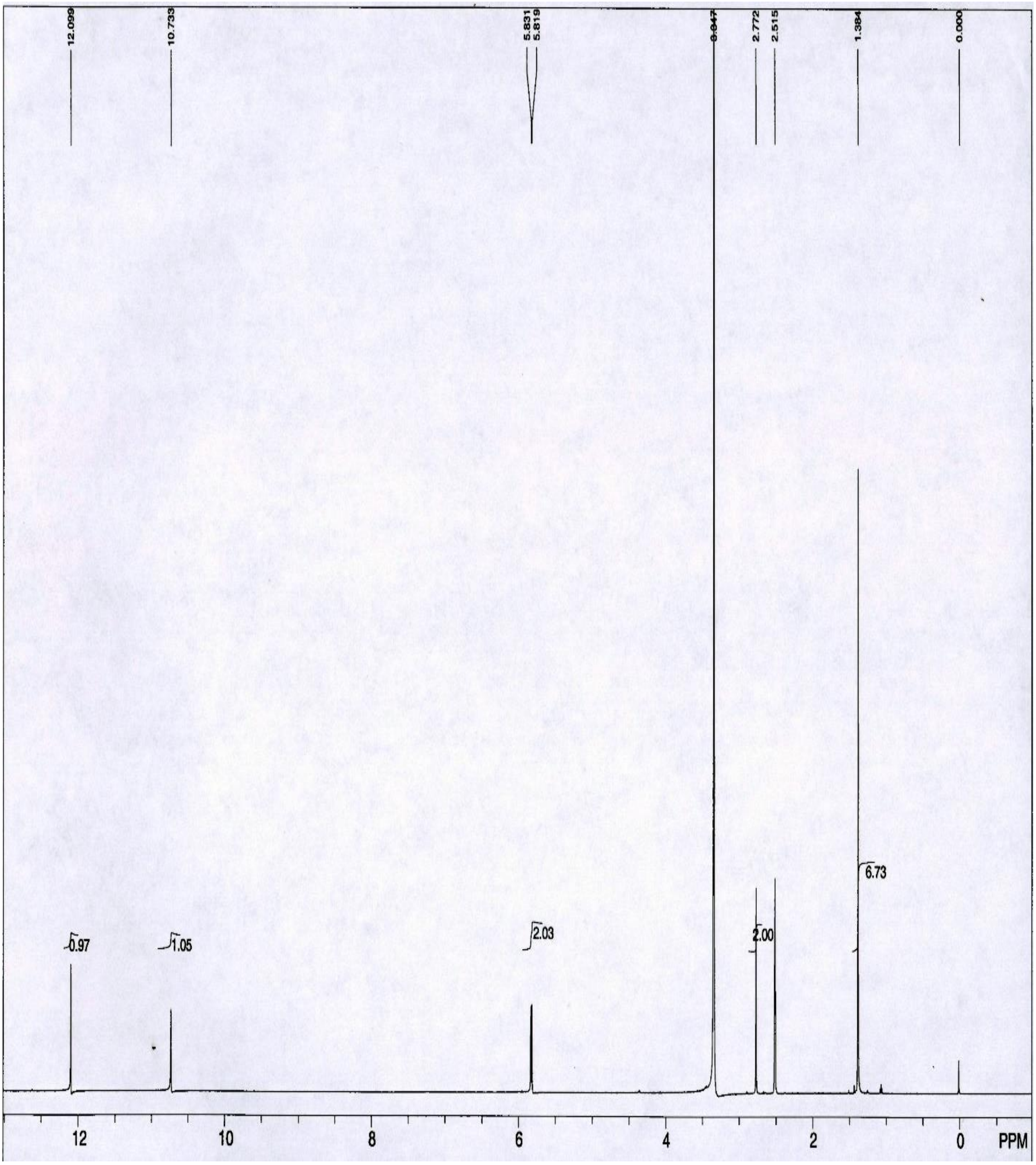

Supplementary Figure S11.- Mass spectrum of 7-methoxy, 8-hydroxy 2,2-dimethyl chroman-4-one (5a).

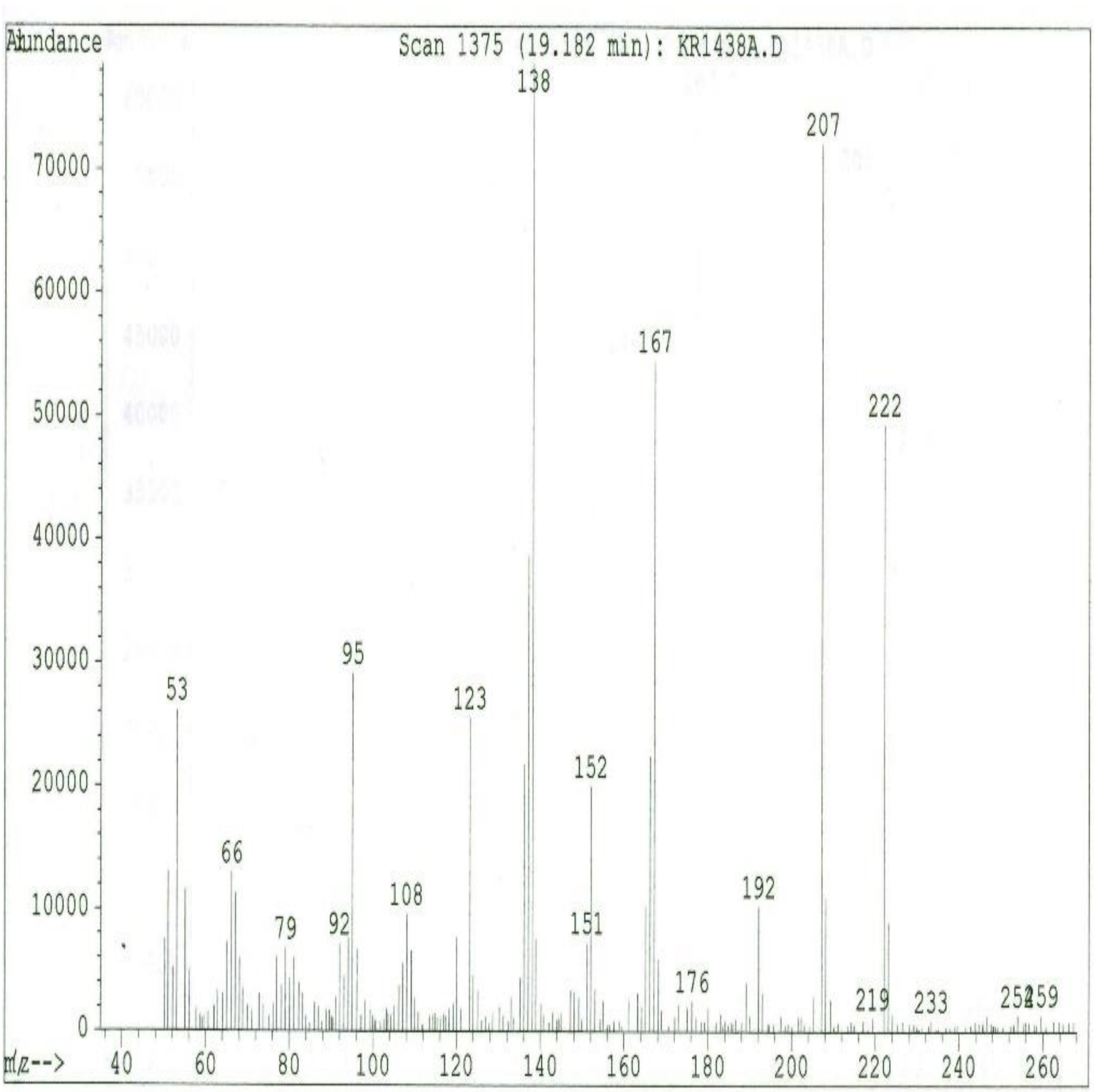

Supplementary Figure *S*12.- Proton chemical shift spectrum of 7-methoxy, 8-hydroxy 2,2-dimethyl chroman-4-one (5a).

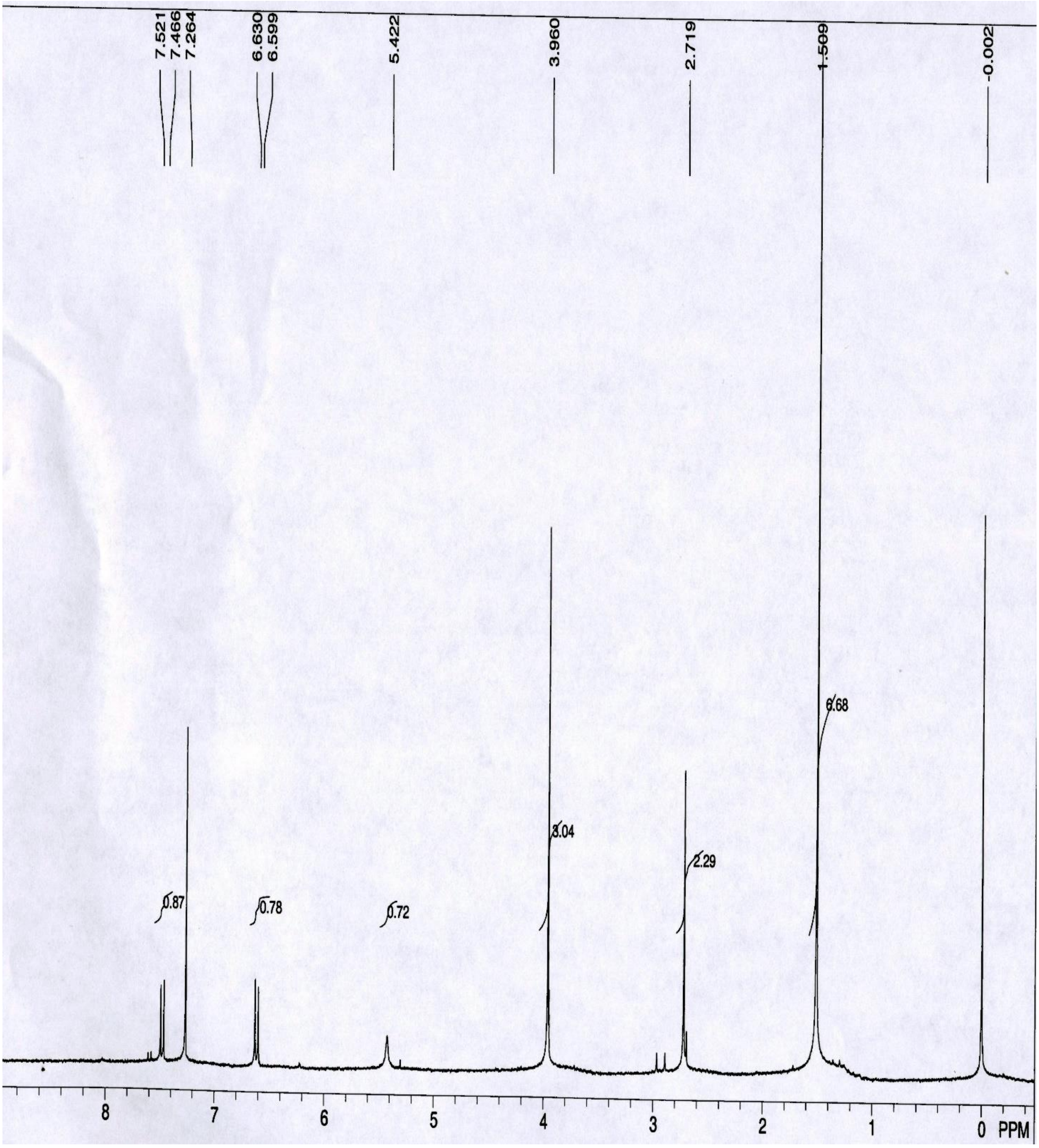

Supplementary Figure *S*13.- Mass spectrum of 7-methoxy, 6-hydroxy 2,2-dimethyl chroman-4-one (5b).

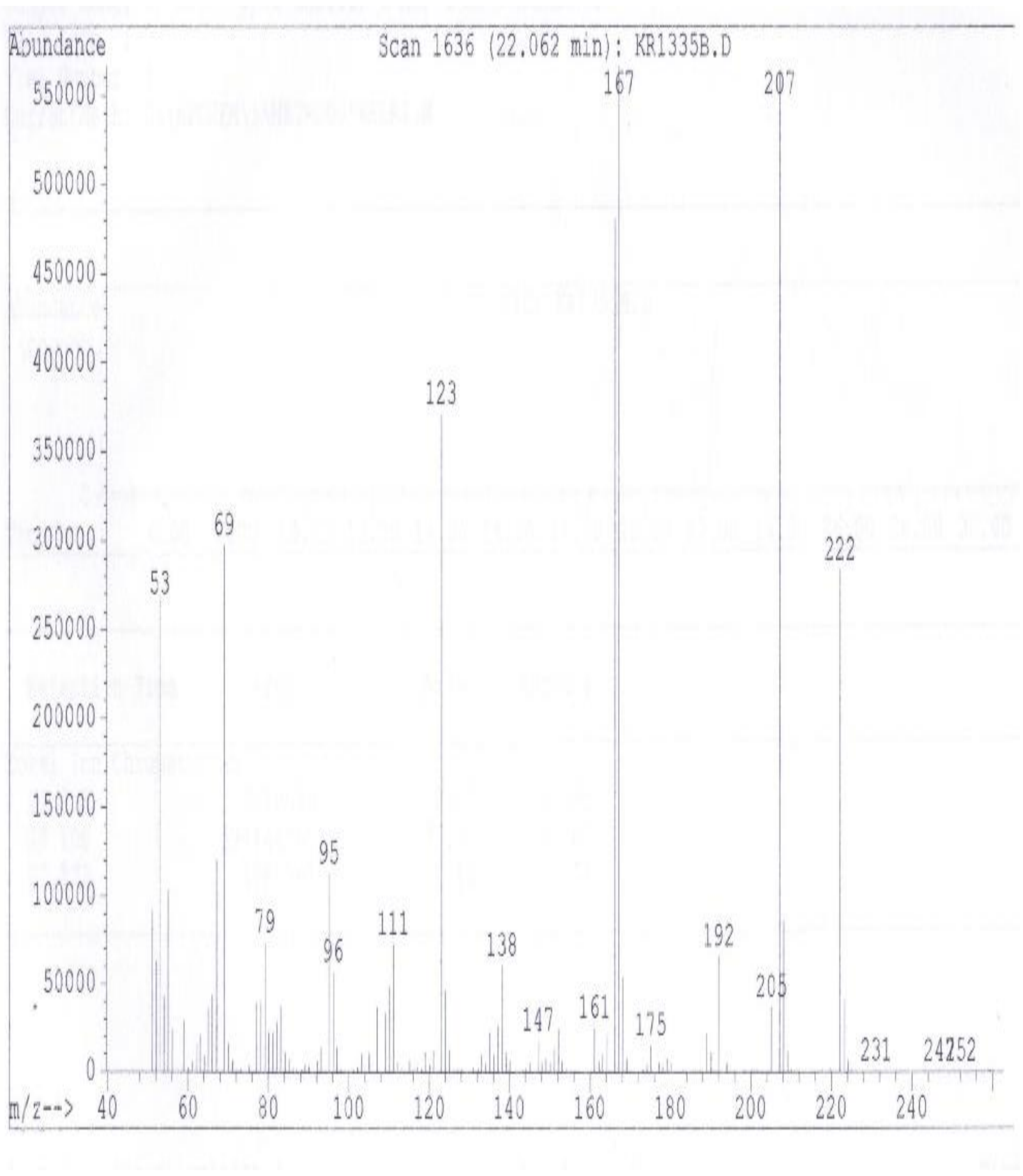

Supplementary Figure *S*14.- Proton chemical shift spectrum of 7-methoxy, 6-hydroxy 2,2-dimethyl chroman-4-one (5b).

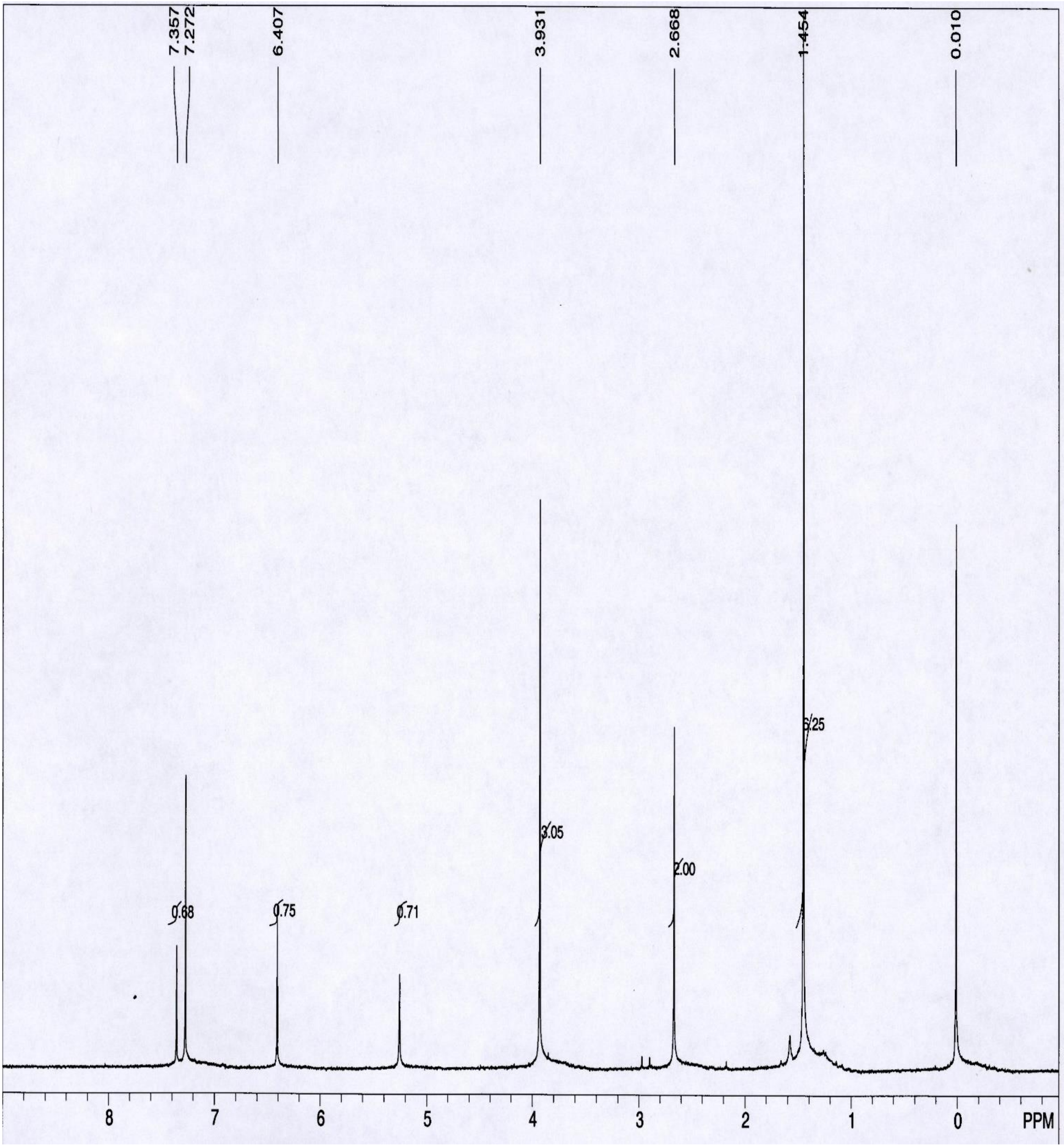

Supplementary Figure *S*15) - Mass spectrum of 7-methoxy, 5-hydroxy 2,2-dimethyl chroman-4-one (5c).

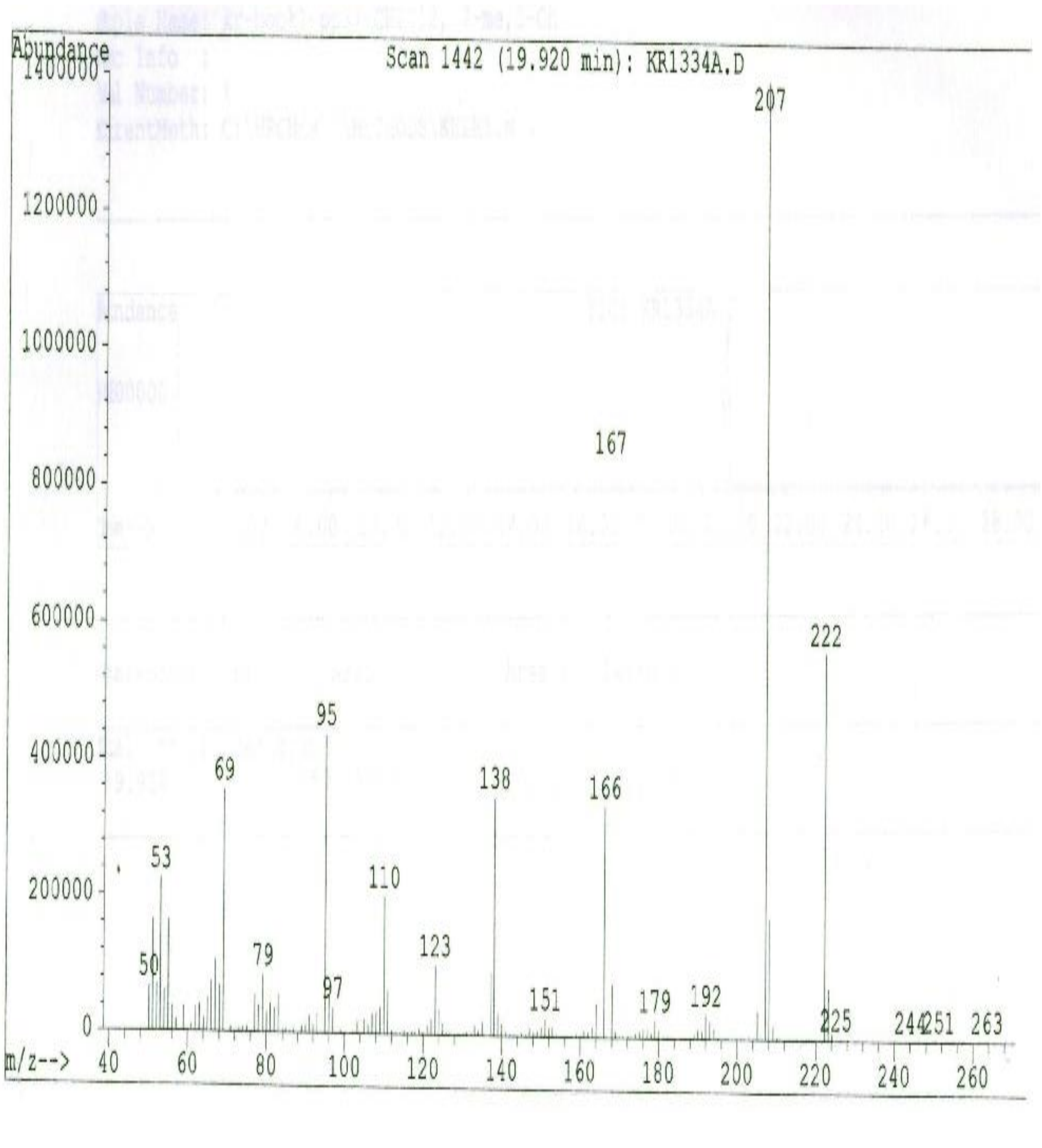

Supplementary Figure *S*16.- Proton chemical shift spectrum of 7-methoxy, 5-hydroxy 2,2-dimethyl chroman-4-one (5c).

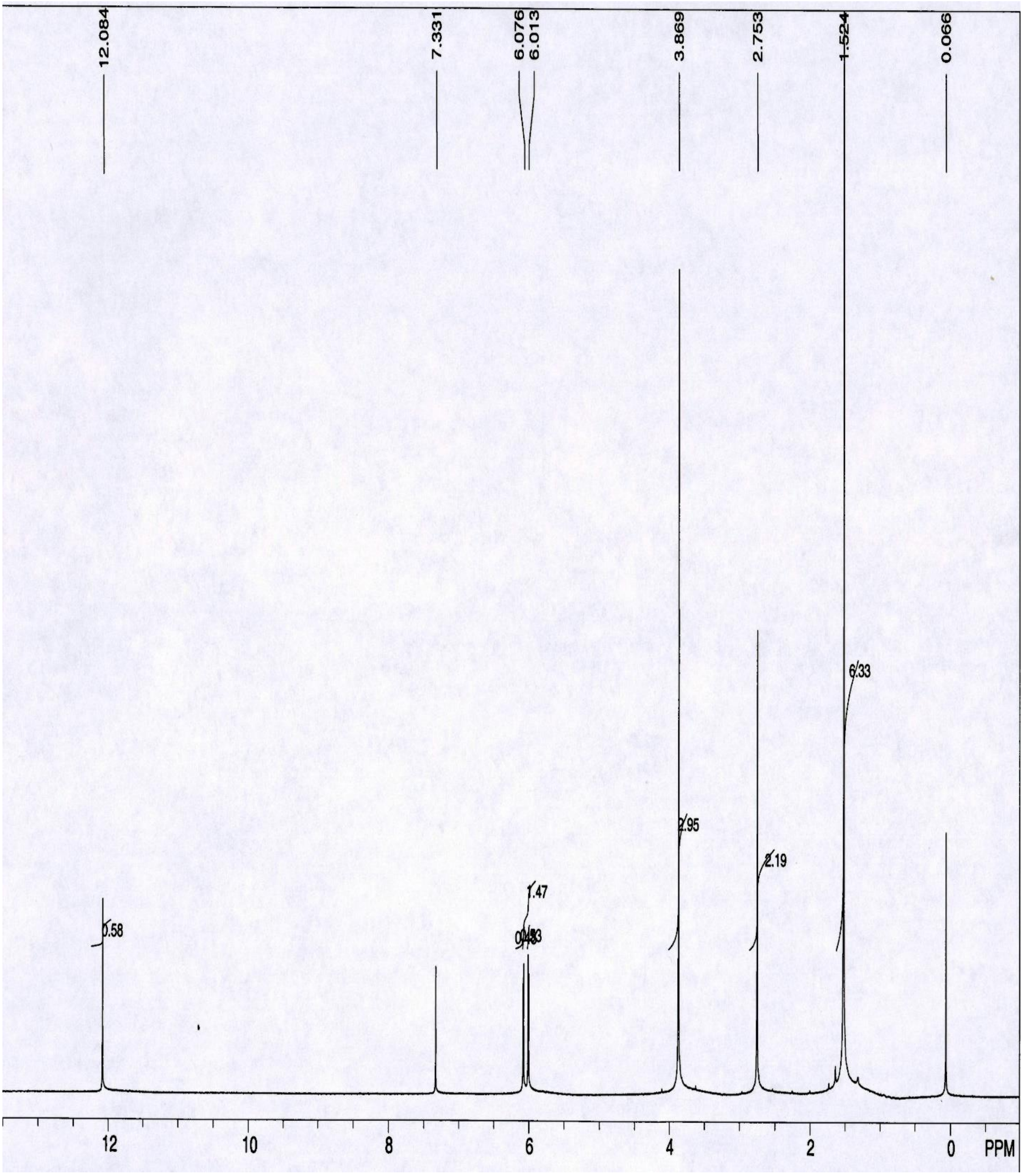

Supplementary Figure *S*17.- Mass spectrum of 7-ethoxy, 8-hydroxy 2,2-dimethyl chroman-4-one (5d).

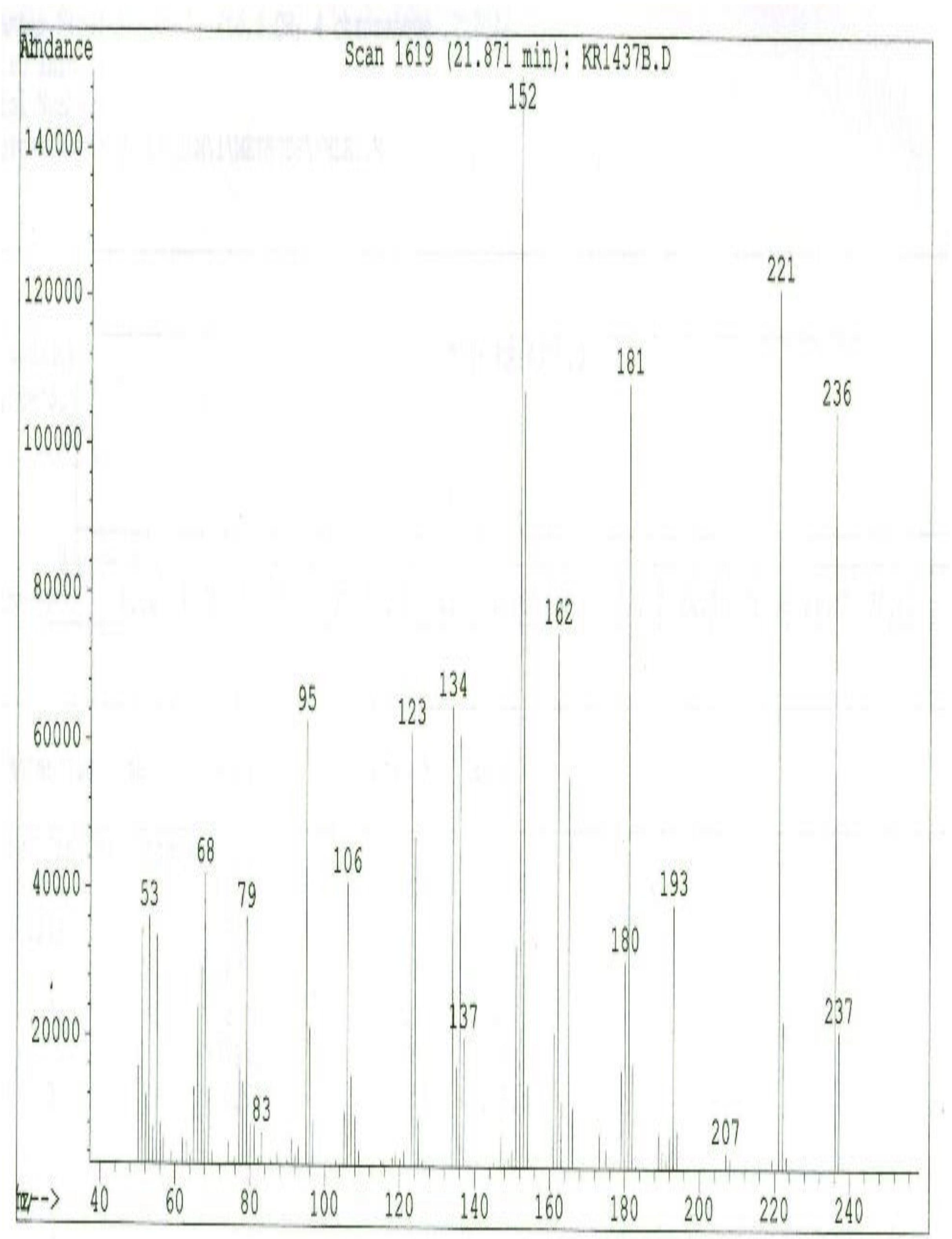

Supplementary Figure *S*18.- Proton chemical shift spectrum of 7-ethoxy, 8-hydroxy 2,2-dimethyl chroman-4-one (5d).

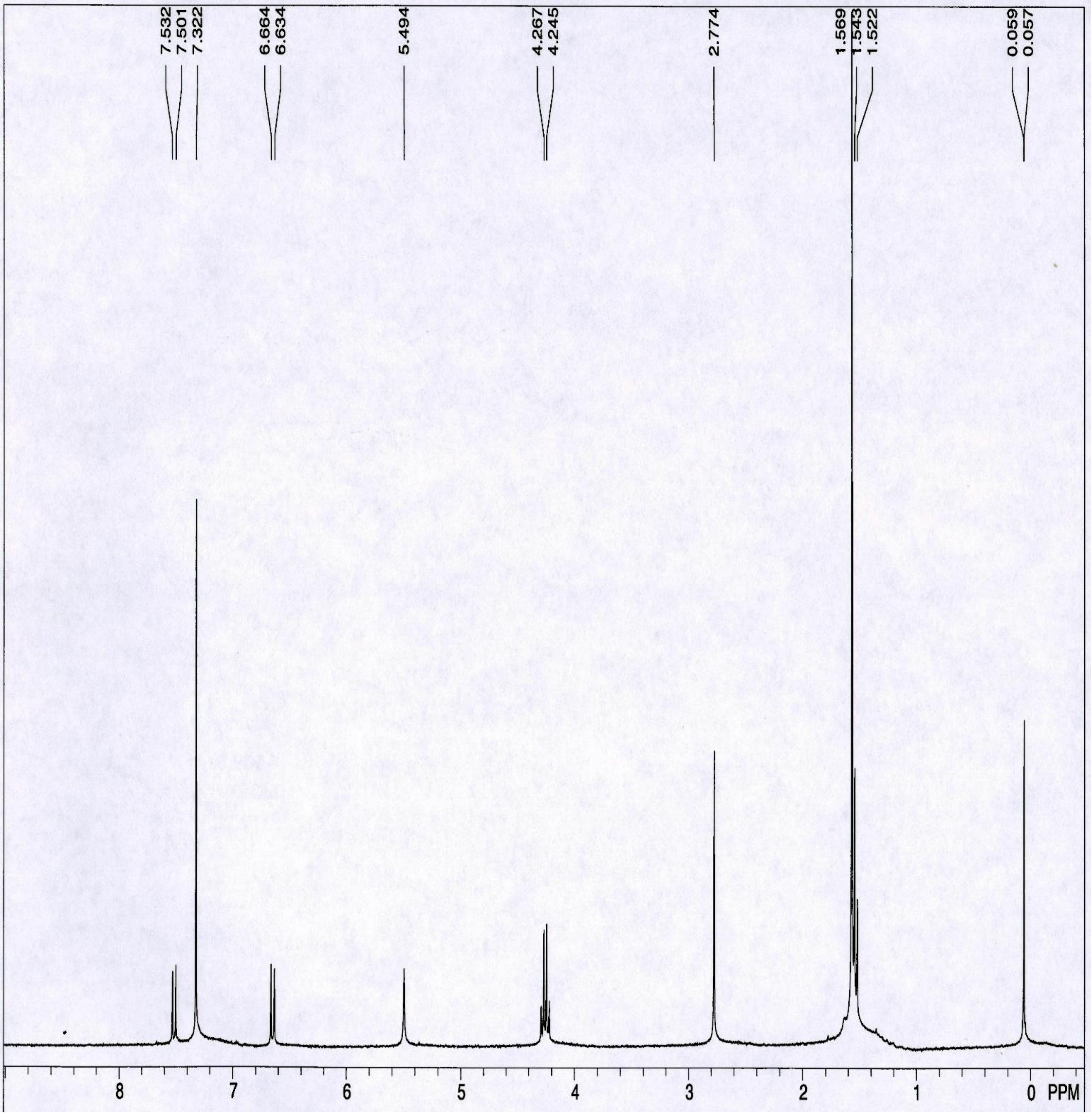

Supplementary Figure *S*19.- Mass spectrum of 7-ethoxy, 6-hydroxy 2,2-dimethyl chroman-4-one (5e).

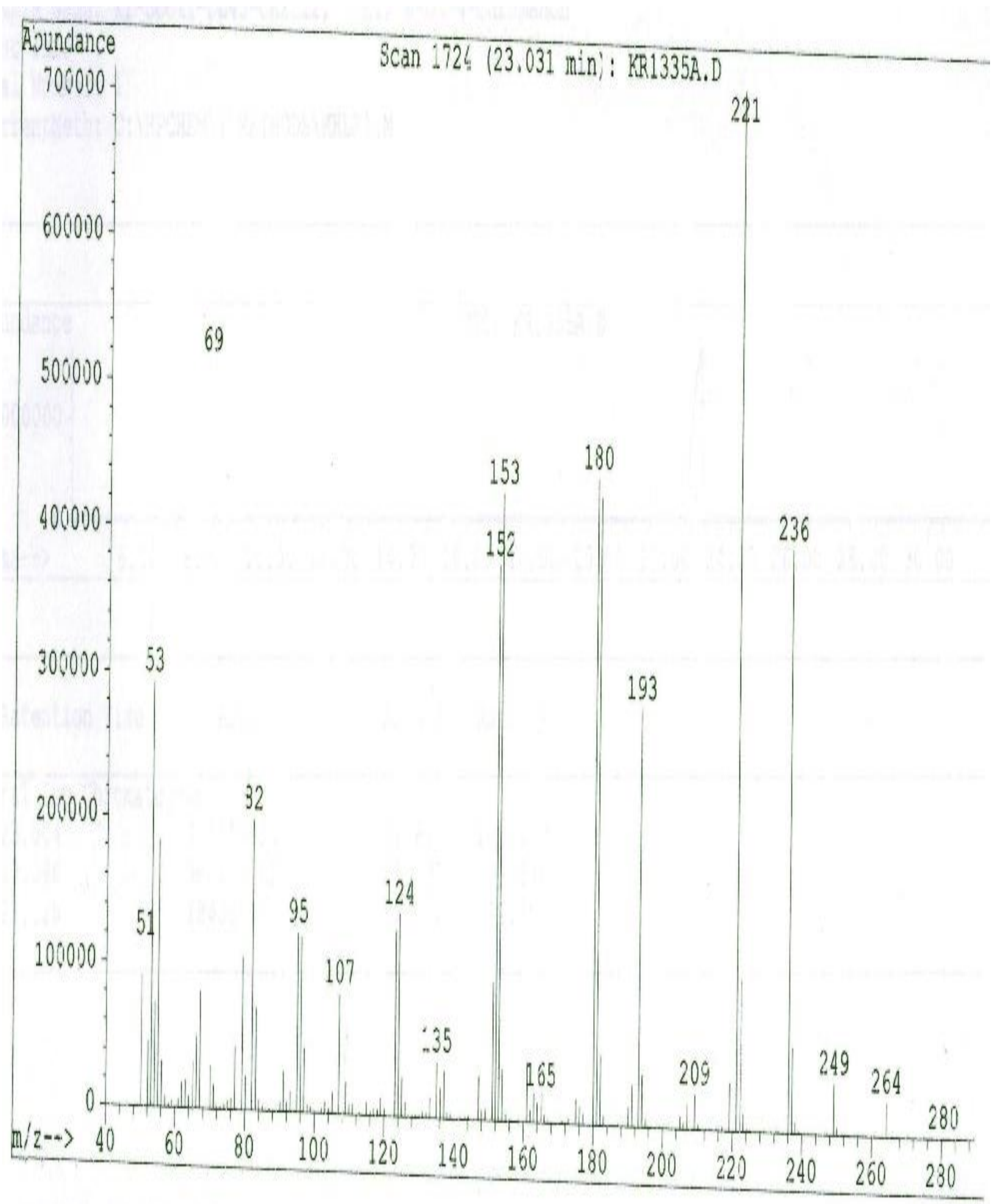

Supplementary Figure *S*20.- Proton chemical shift spectrum of 7-ethoxy, 6-hydroxy 2,2-dimethyl chroman-4-one (5e).

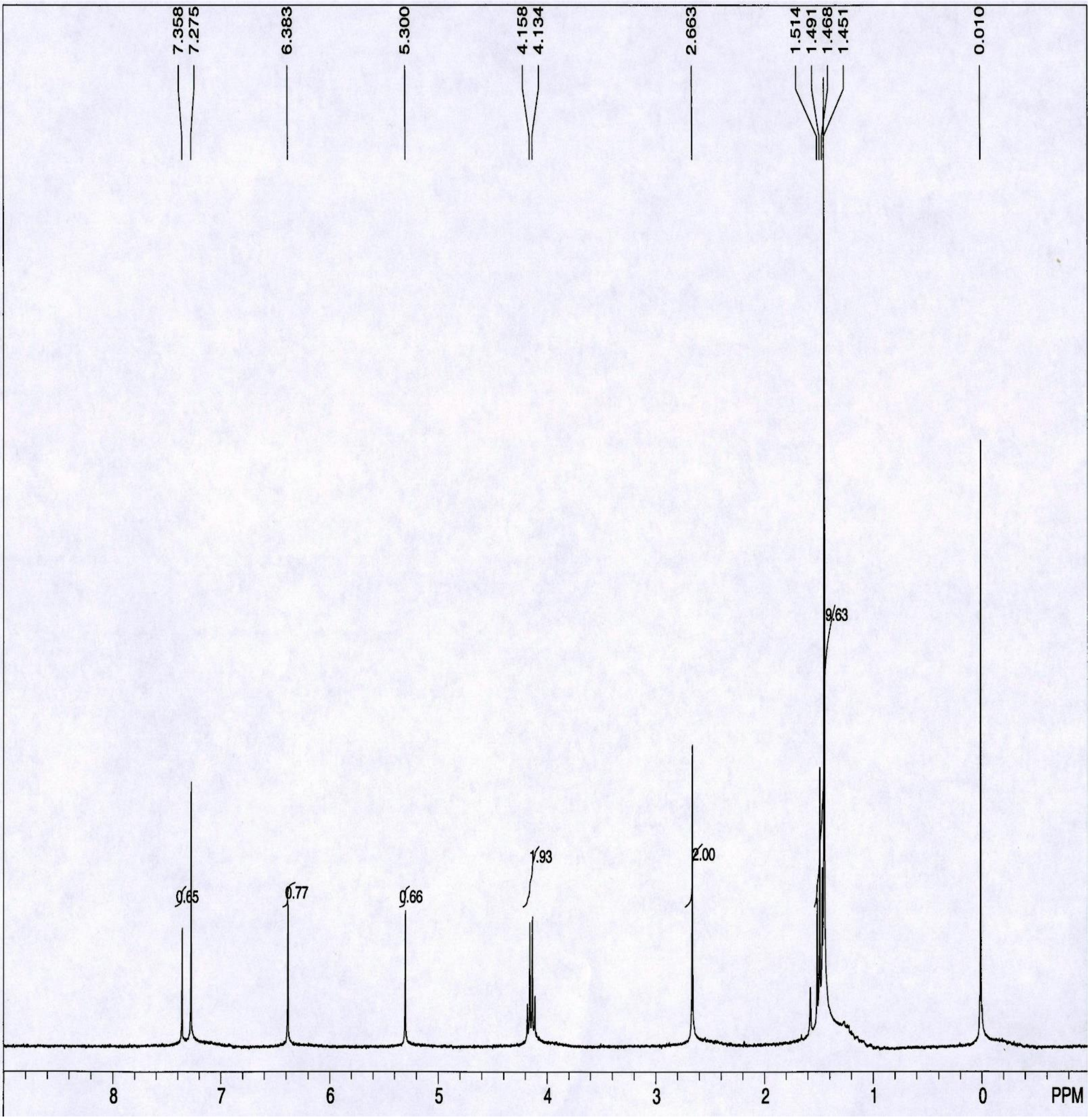

Supplementary Figure *S*21) - Mass spectrum of 7-ethoxy, 5-hydroxy 2,2-dimethyl chroman-4-one (5f).

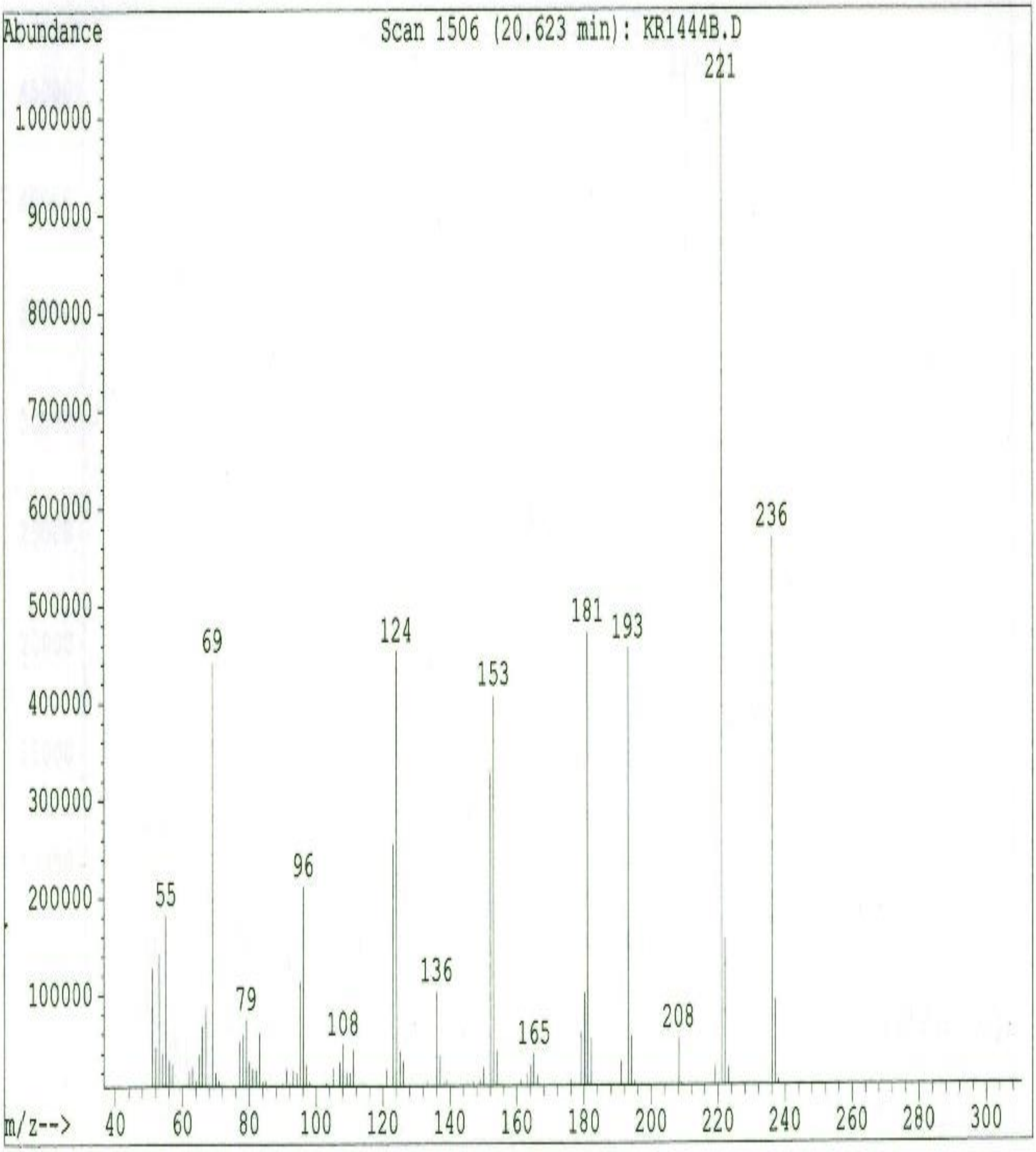

Supplementary Figure *S*22.- Proton chemical shift spectrum of 7-ethoxy, 5-hydroxy 2,2-dimethyl chroman-4-one (5f).

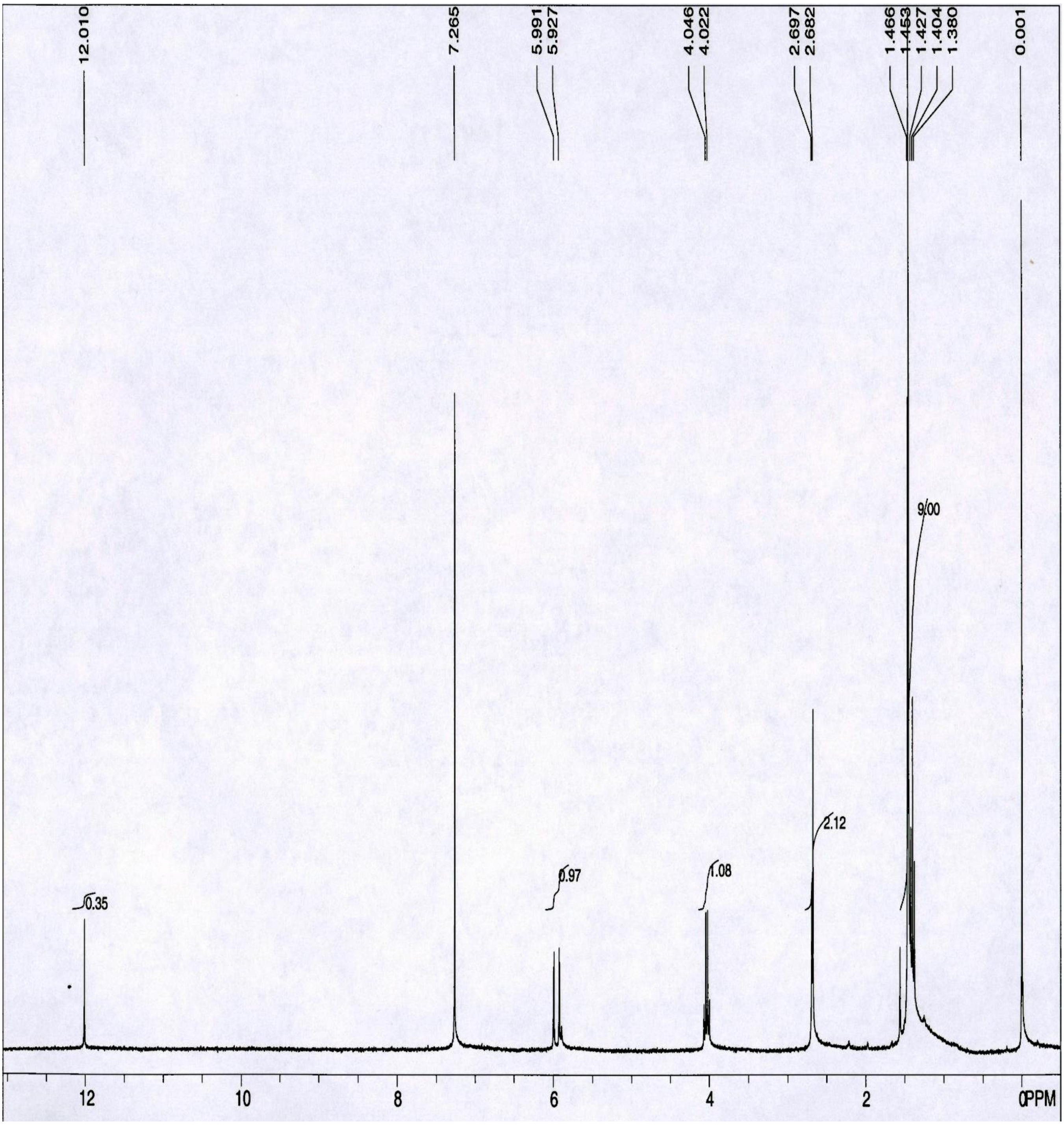

Supplementary Figure *S*23.- Mass spectrum of 7,8-dimethoxy, 2,2-dimethyl chroman-4-one (6a).

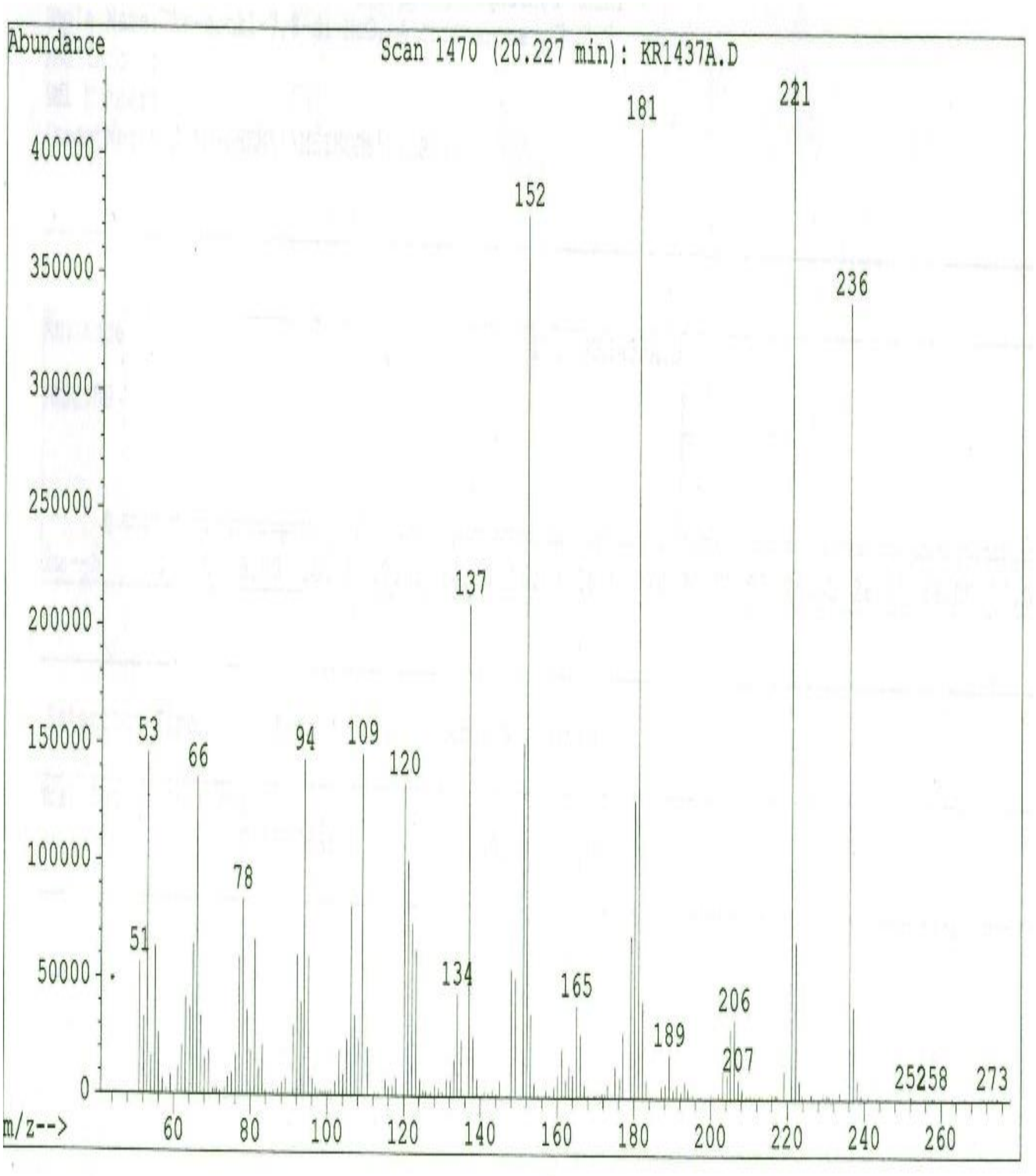

Supplementary Figure *S*24.- Proton chemical shift spectrum of 7,8-dimethoxy, 2,2-dimethyl chroman-4-one (6a).

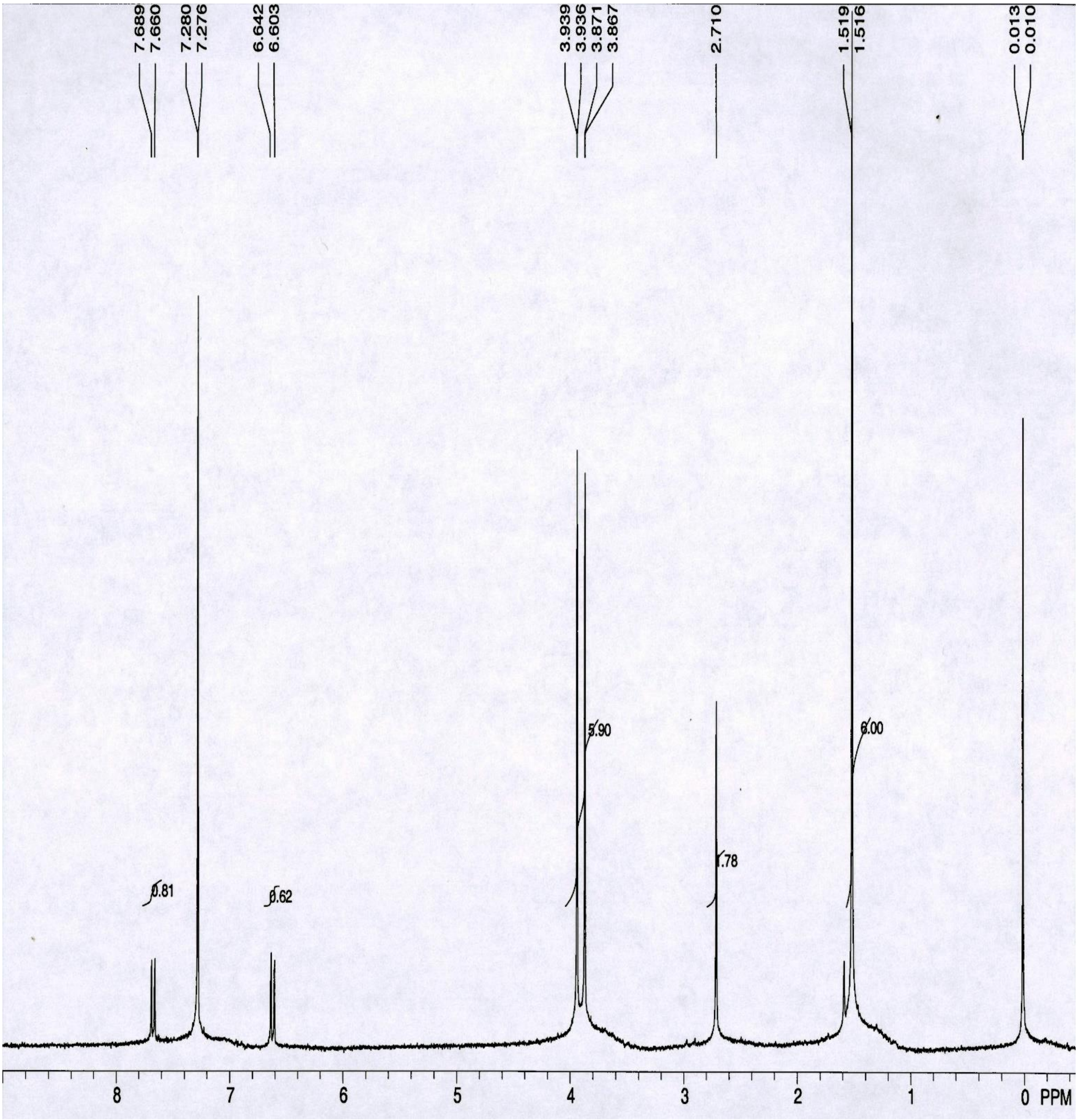

Supplementary Figure *S*25.- Mass spectrum of 6,7-dimethoxy, 2,2-dimethyl chroman-4-one (6b).

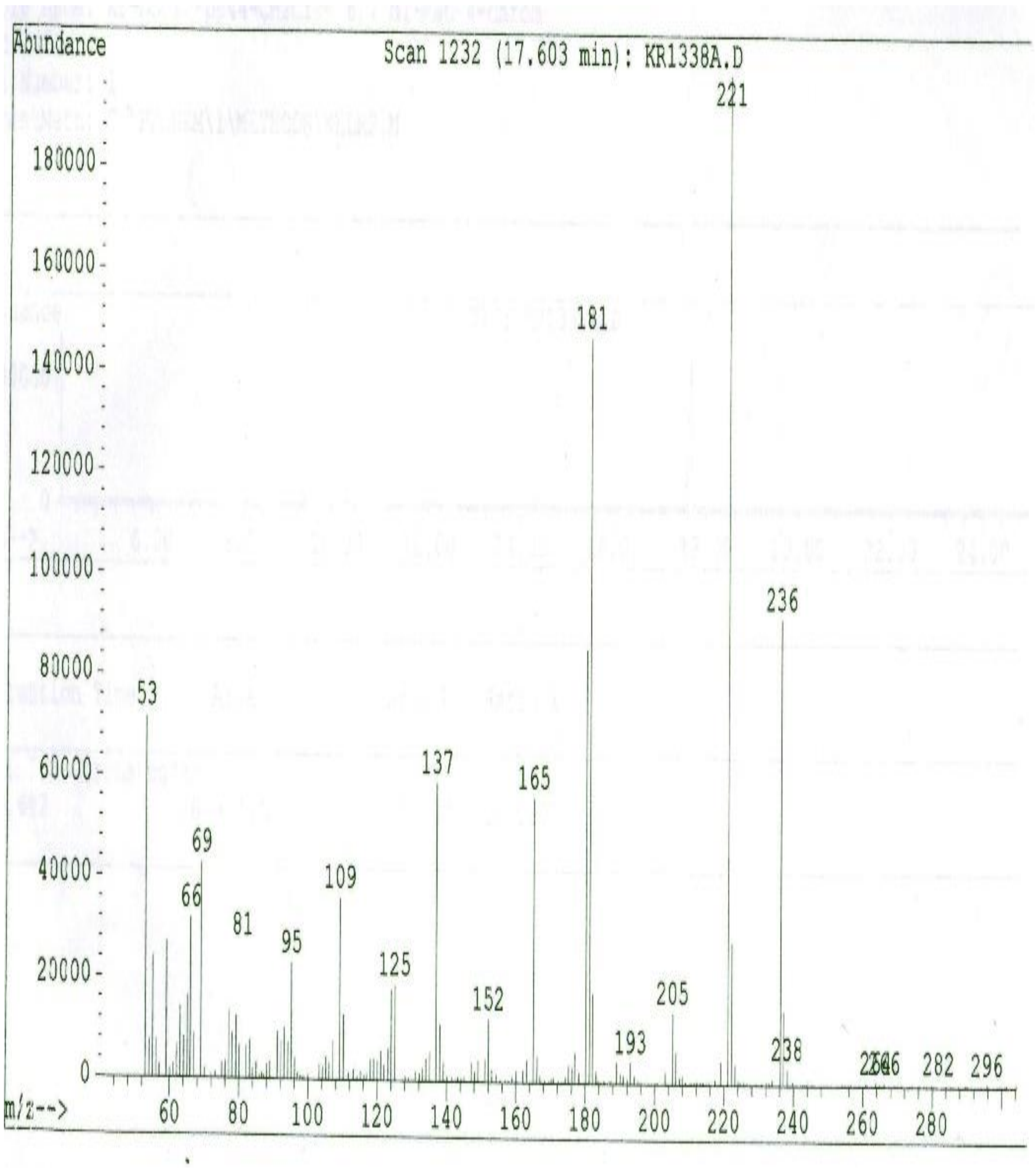

Supplementary Figure *S*26.- Proton chemical shift spectrum of 6,7-dimethoxy, 2,2-dimethyl chroman-4-one (6b).

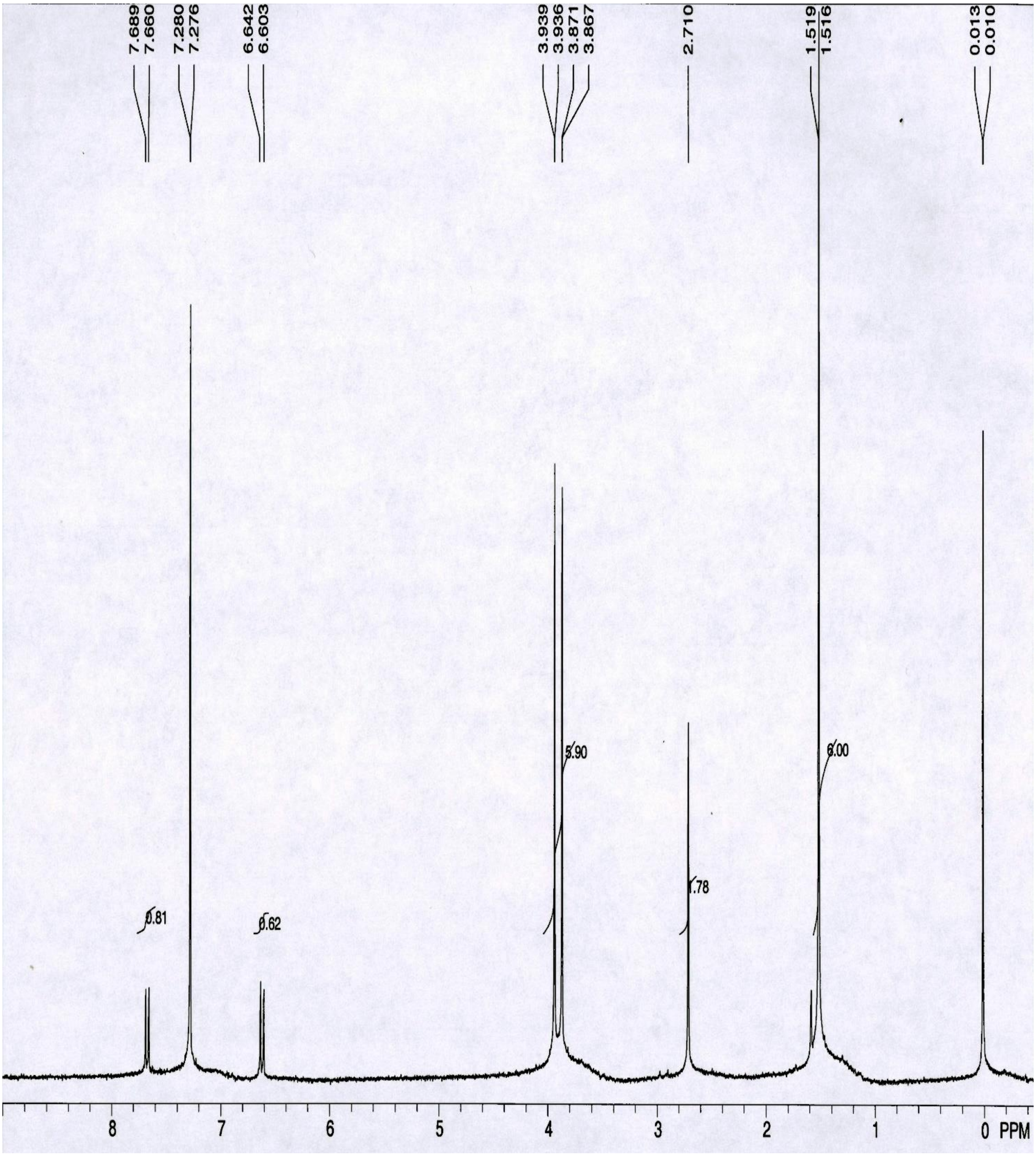

Supplementary Figure *S*27) - Mass spectrum of 5,7-dimethoxy 2, 2-dimethyl chroman-4-one (6c).

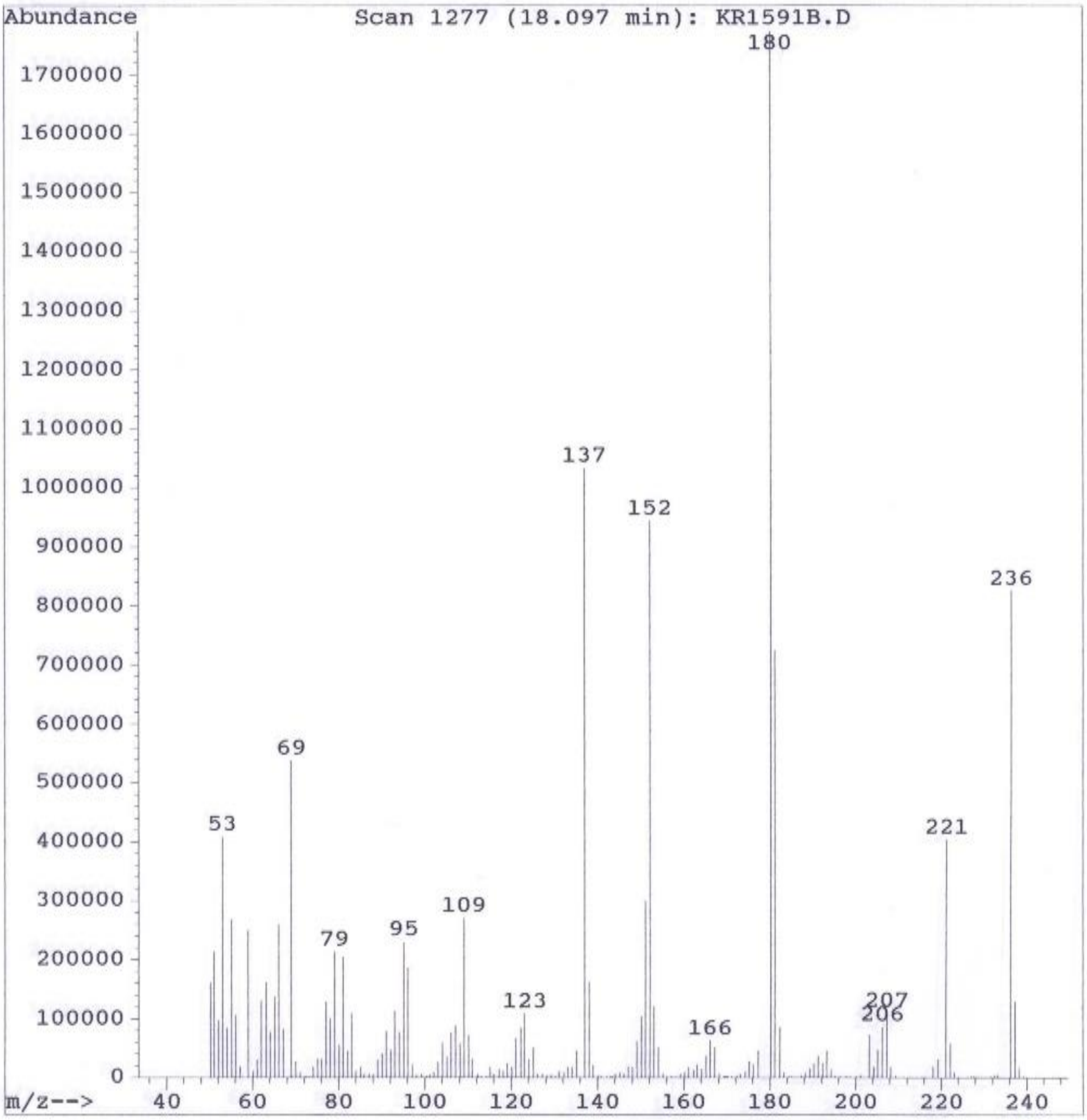

Supplementary Figure *S*28.- Proton chemical shift spectrum of 5,7-dimethoxy 2,2-dimethyl chroman-4-one (6c).

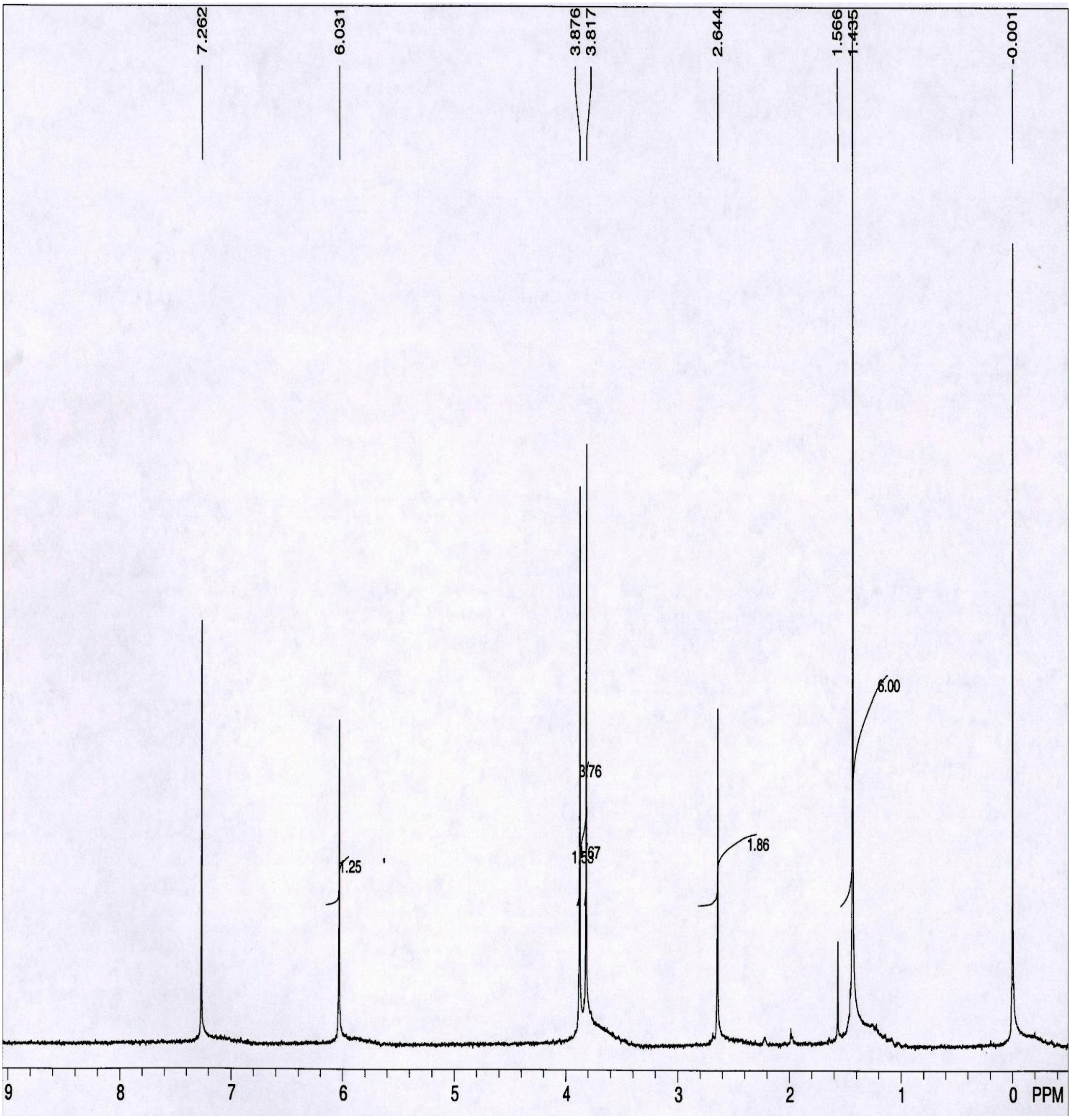

Supplementary Figure *S*29) - Mass spectrum of 7-ethoxy,8-mehoxy 2, 2-dimethyl chroman-4-one (6d).

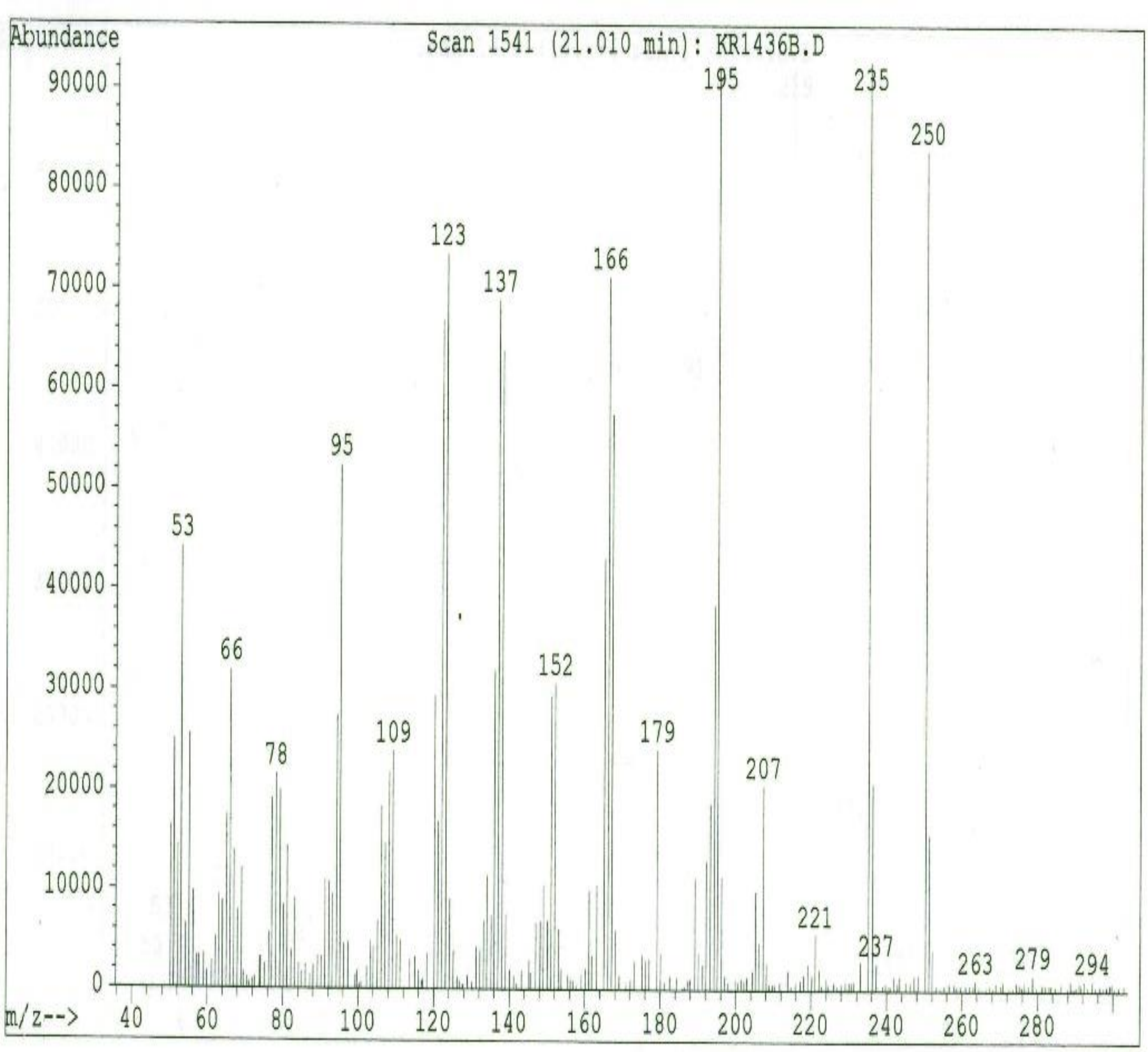

Supplementary Figure *S*30.- Proton chemical shift spectrum of 7-ethoxy,8-mehoxy 2, 2-dimethyl chroman-4-one (6d).

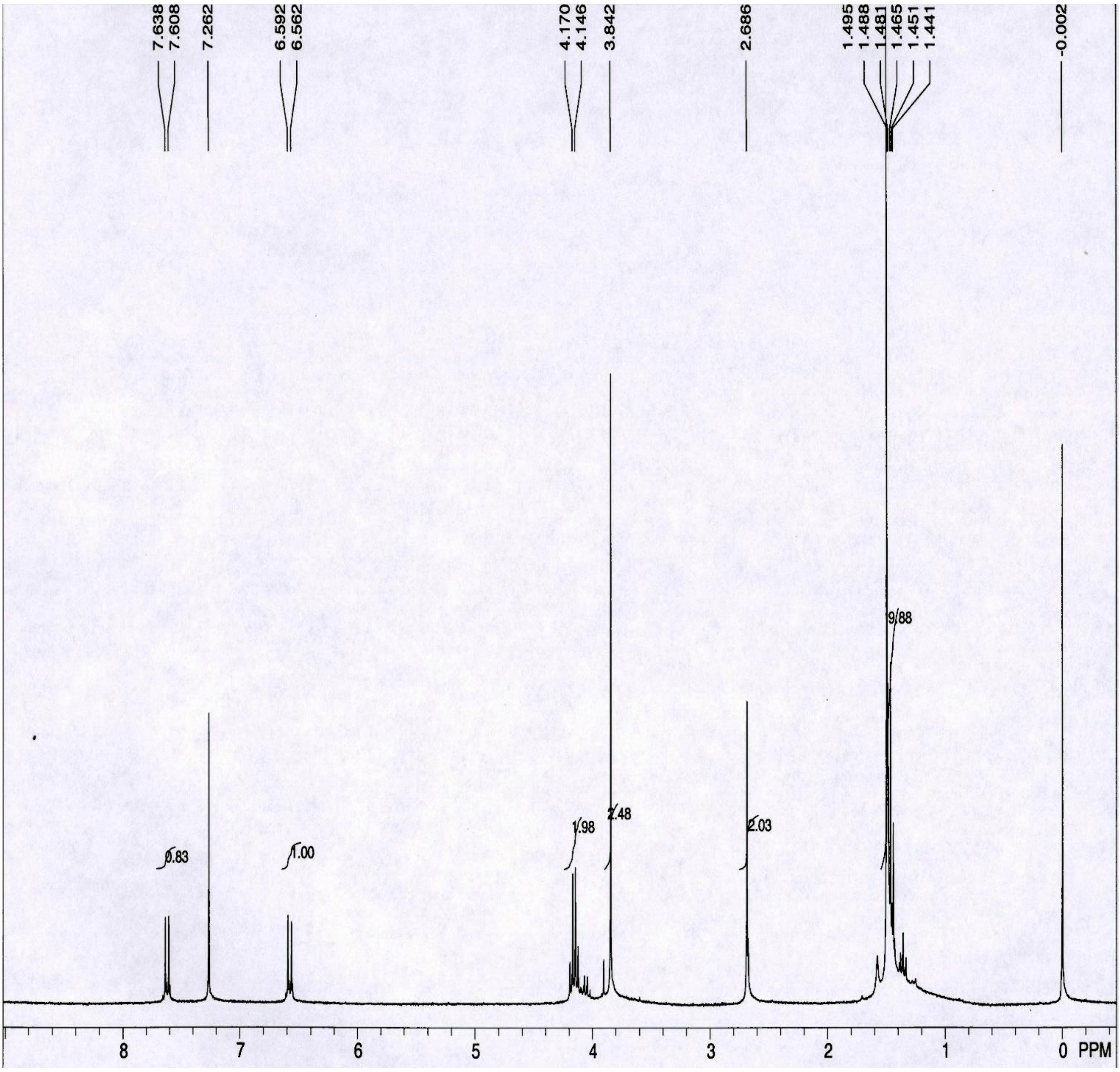

Supplementary Figure *S*31) - Mass spectrum of 7-ethoxy,6-mehoxy 2, 2-dimethyl chroman-4-one (6e).

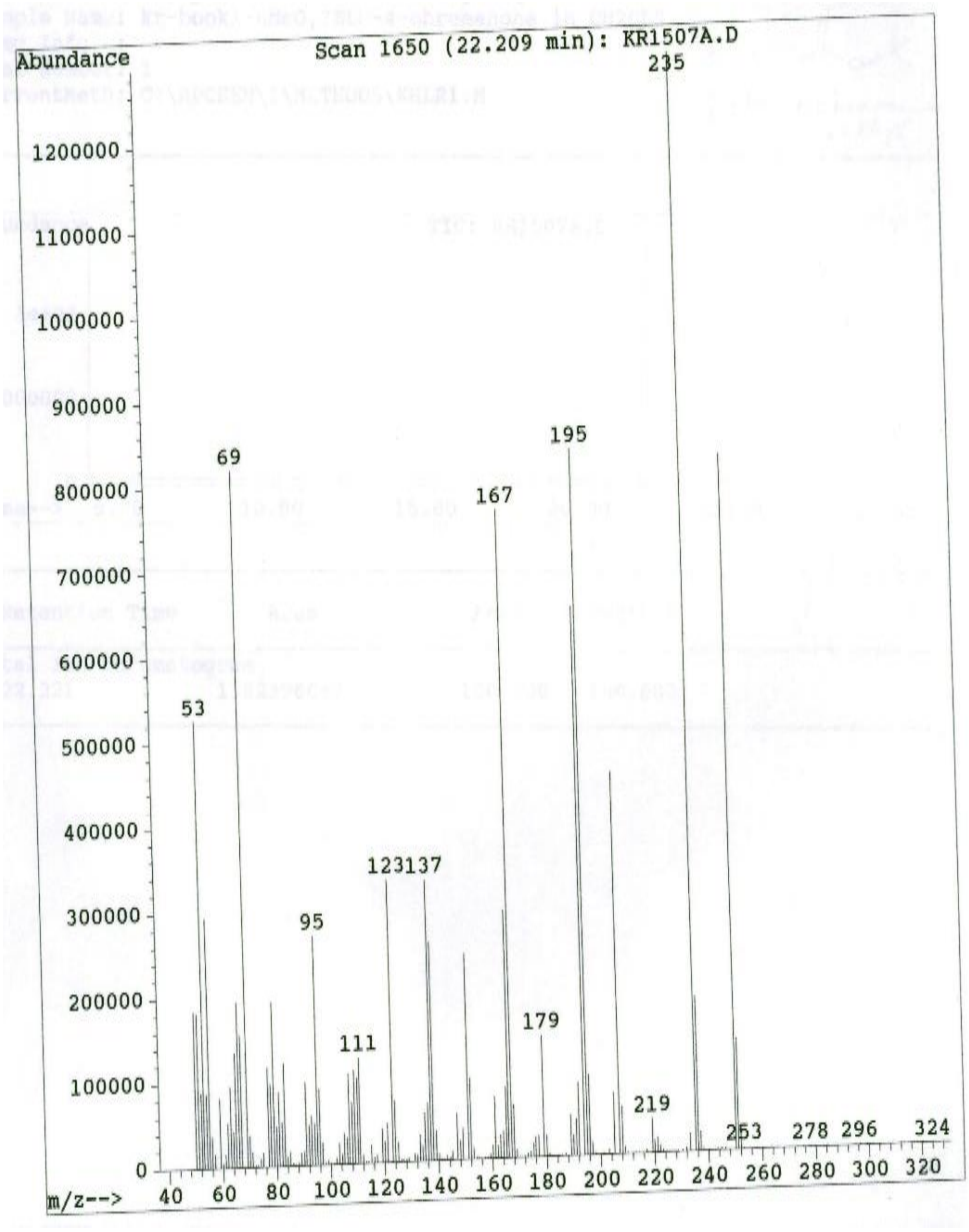

Supplementary Figure *S*32.- Proton chemical shift spectrum of 7-ethoxy,6-mehoxy 2, 2-dimethyl chroman-4-one (6e).

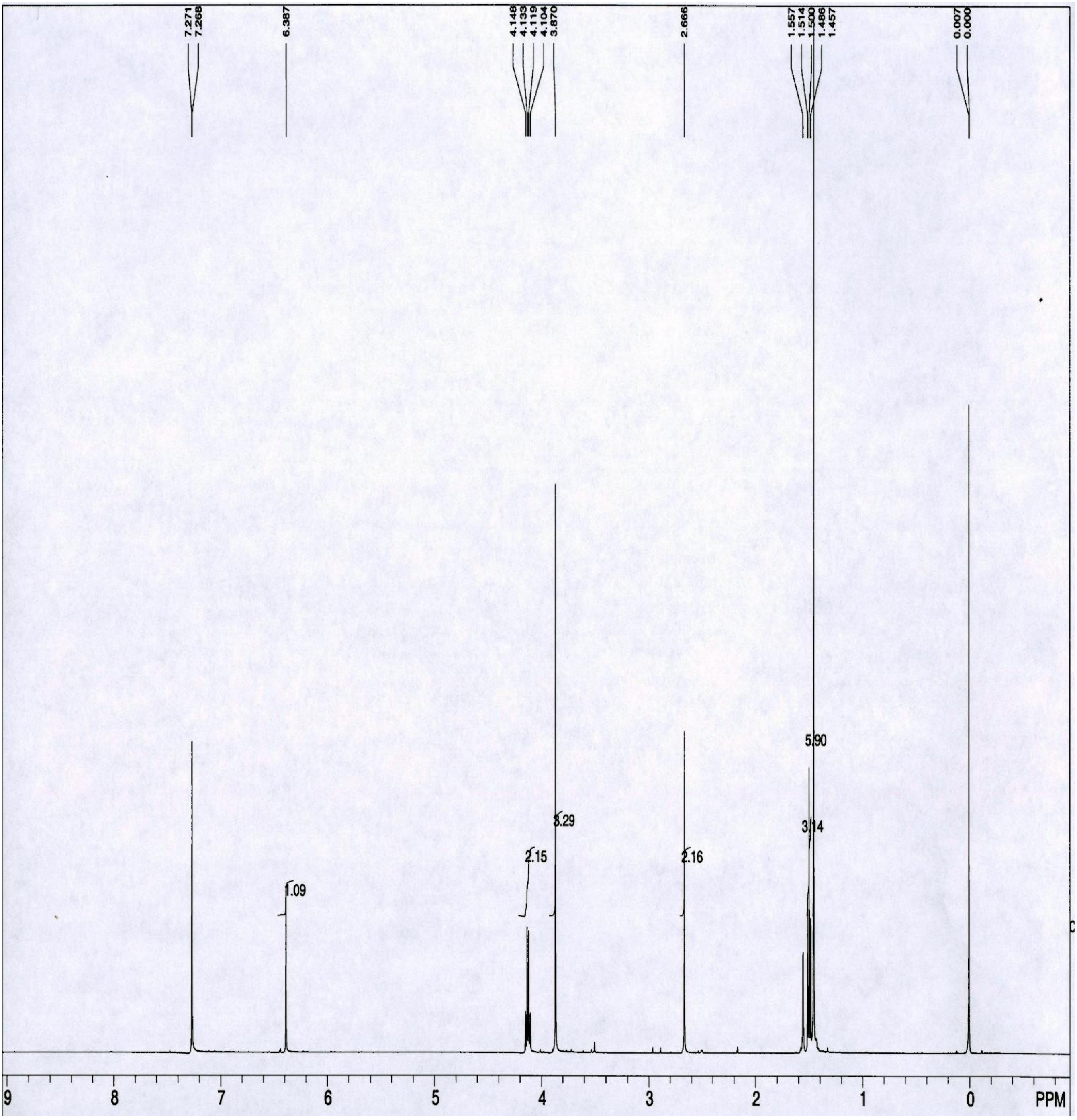

Supplementary Figure *S*33) - Mass spectrum of 7-ethoxy,5-mehoxy 2, 2-dimethyl chroman-4-one (6f).

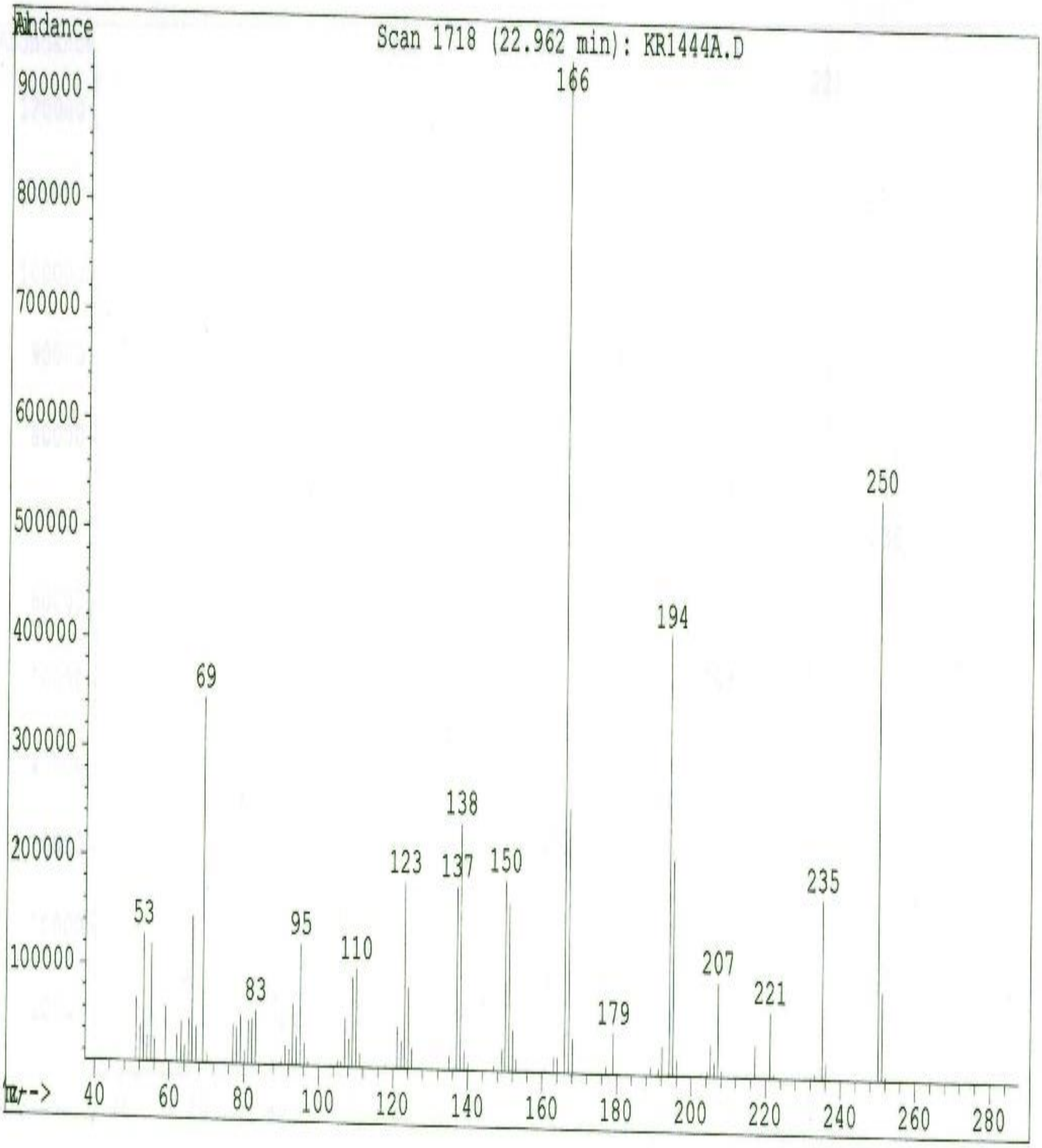

Supplementary Figure *S*34.- Proton chemical shift spectrum of 7-ethoxy,5-mehoxy 2, 2-dimethyl chroman-4-one (6f).

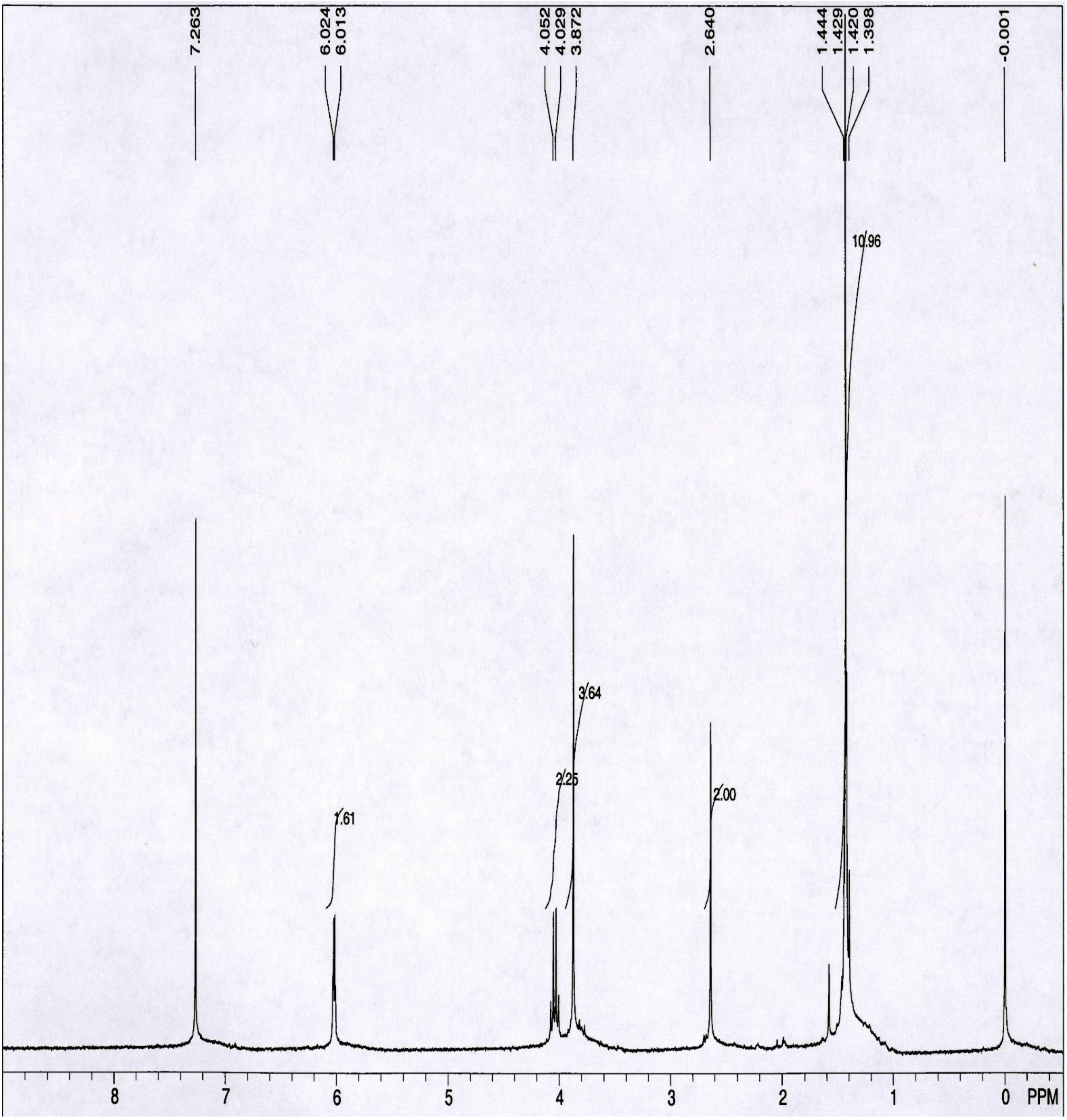

Supplementary Figure *S*35.- Mass spectrum of 7,8-dimethoxy, 2,2-dimethyl *2H*-1-chromene (7a).

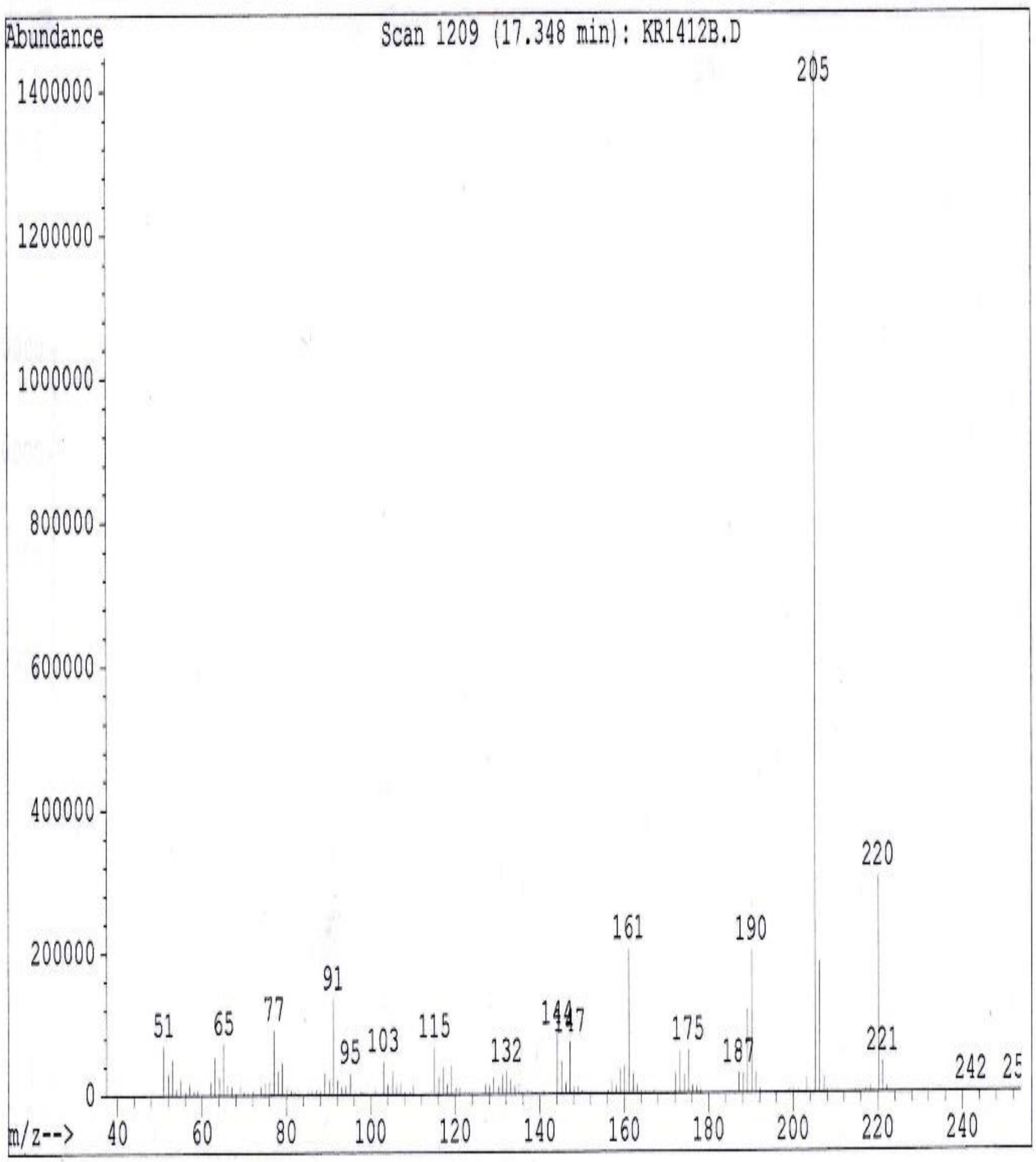

Supplementary Figure *S*36.- Proton chemical shift spectrum of 7,8-dimethoxy 2,2-dimethyl *2H*-1-chromene (7a).

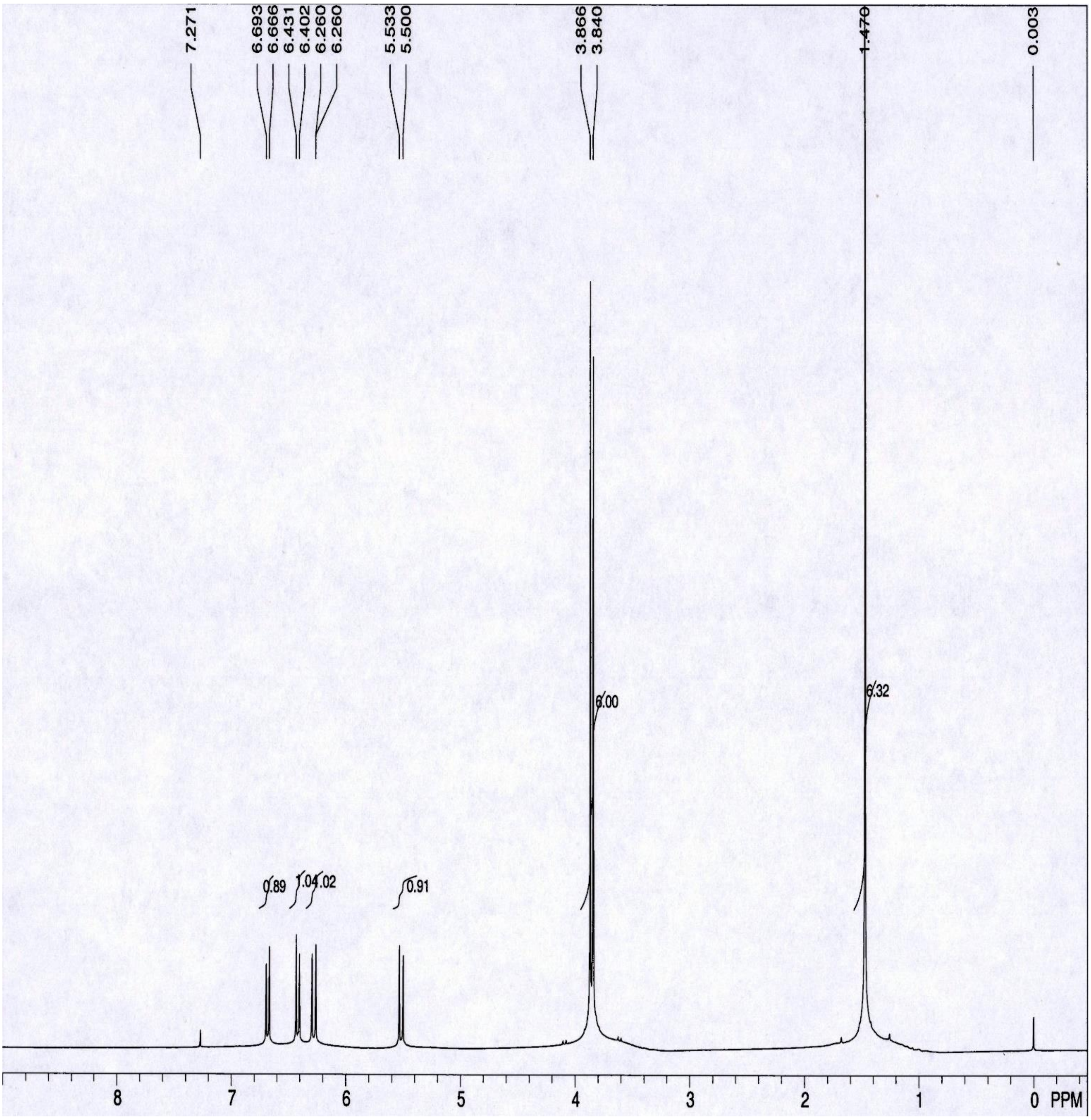

Supplementary Figure *S*37.- Carbon chemical shift spectrum of 7,8-dimethoxy 2,2-dimethyl *2H*-1-chromene (7a).

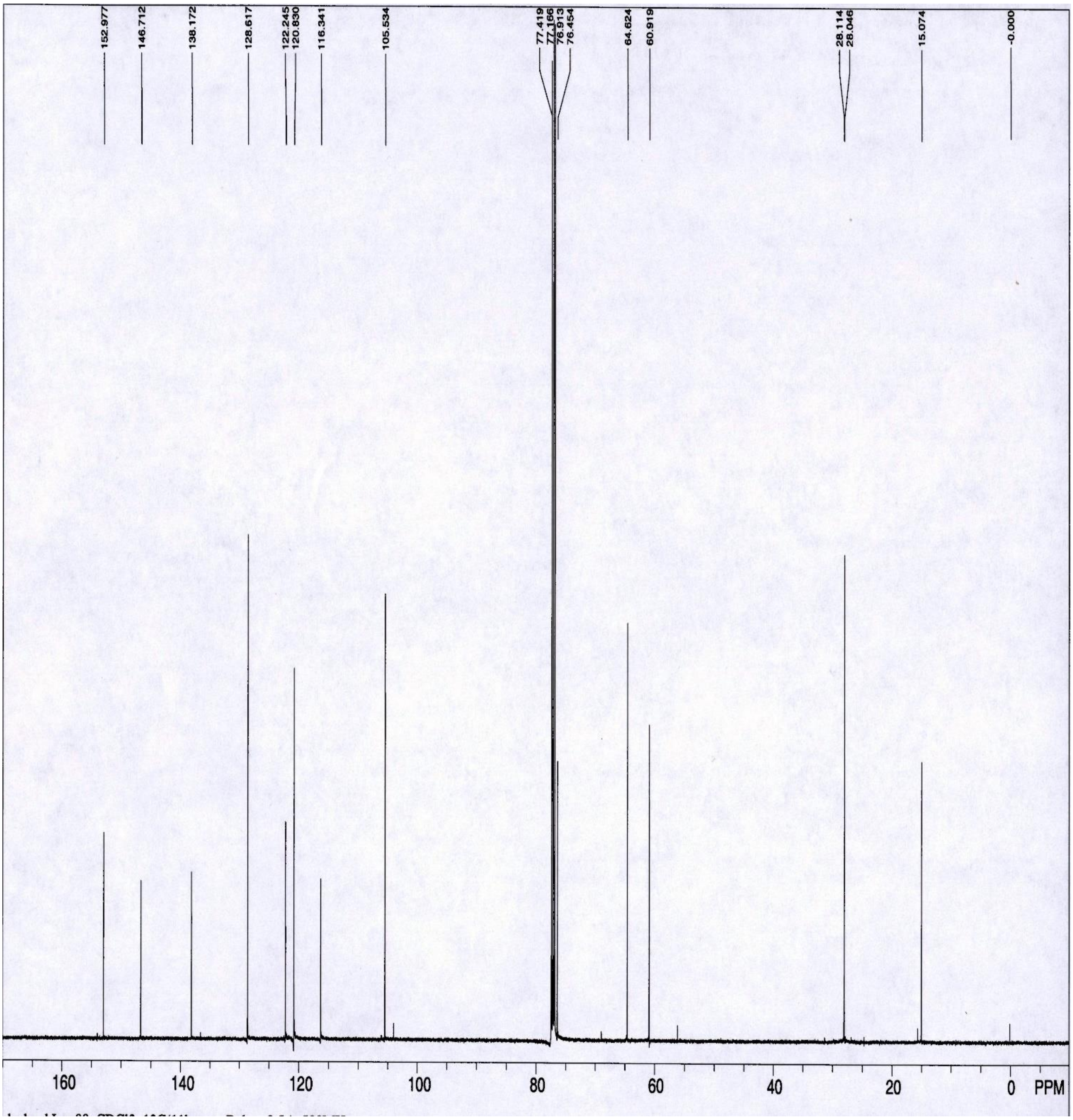

Supplementary Figure *S*38.- Mass spectrum of 6,7-dimethoxy, 2,2-dimethyl *2H*-1-chromene (7b) (Precocene II).

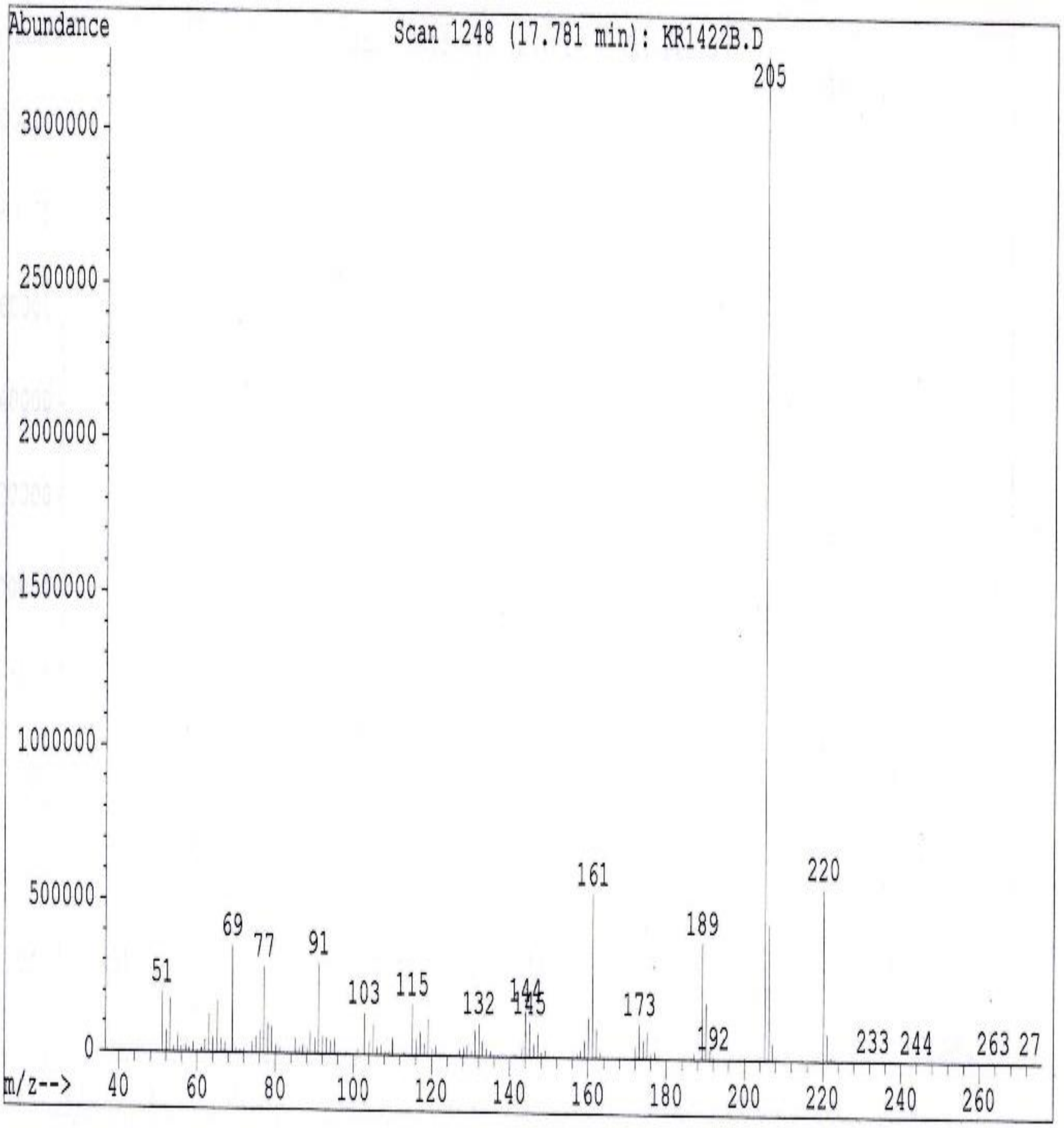

Supplementary Figure *S*39.- Proton chemical shift spectrum of 6,7-dimethoxy 2,2-dimethyl *2H*-1-chromene (7b) (Precocene II).

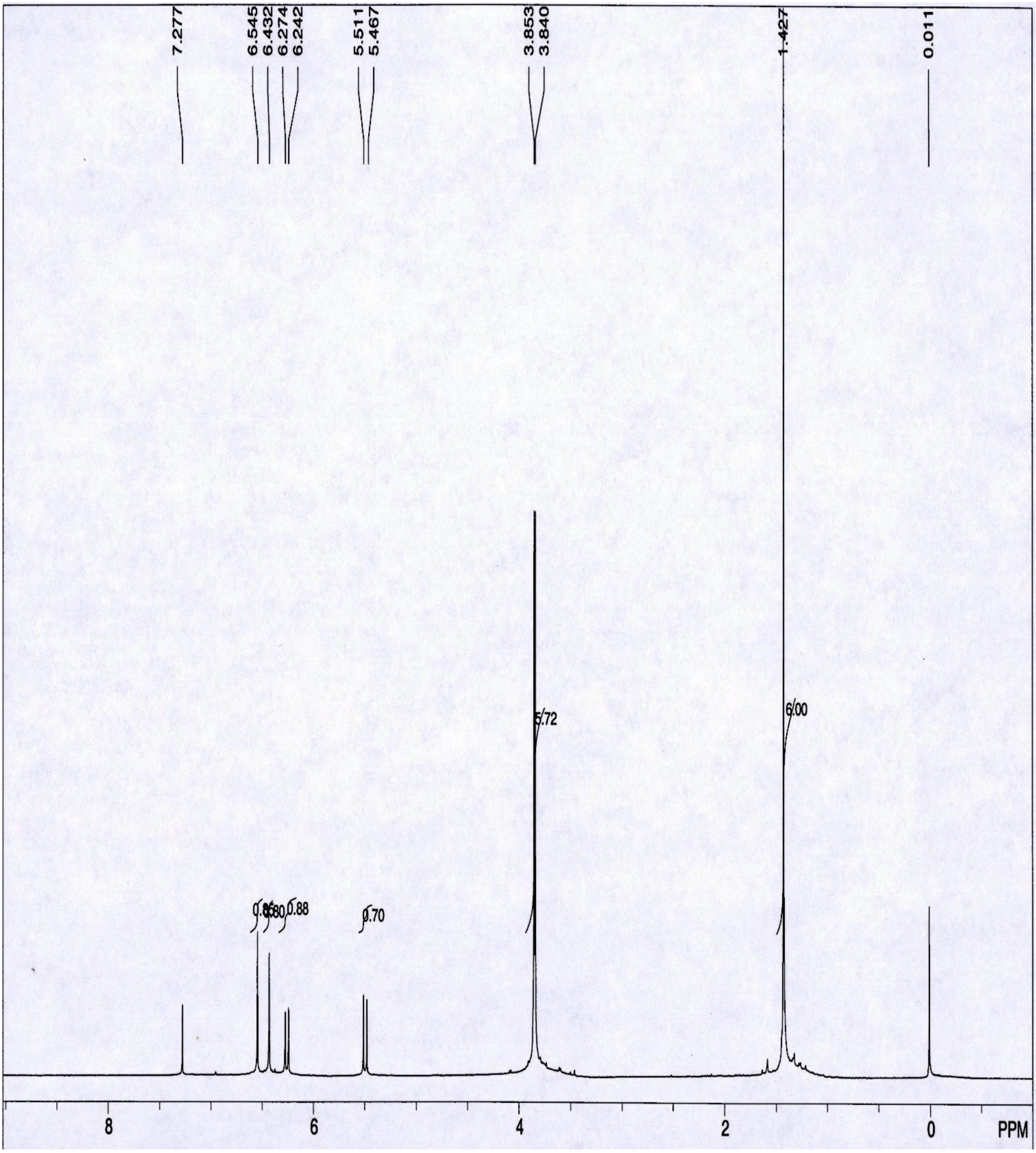

Supplementary Figure *S*40.- Carbon chemical shift spectrum of 6,7-dimethoxy 2,2-dimethyl *2H*-1-chromene (7b) (Precocene II).

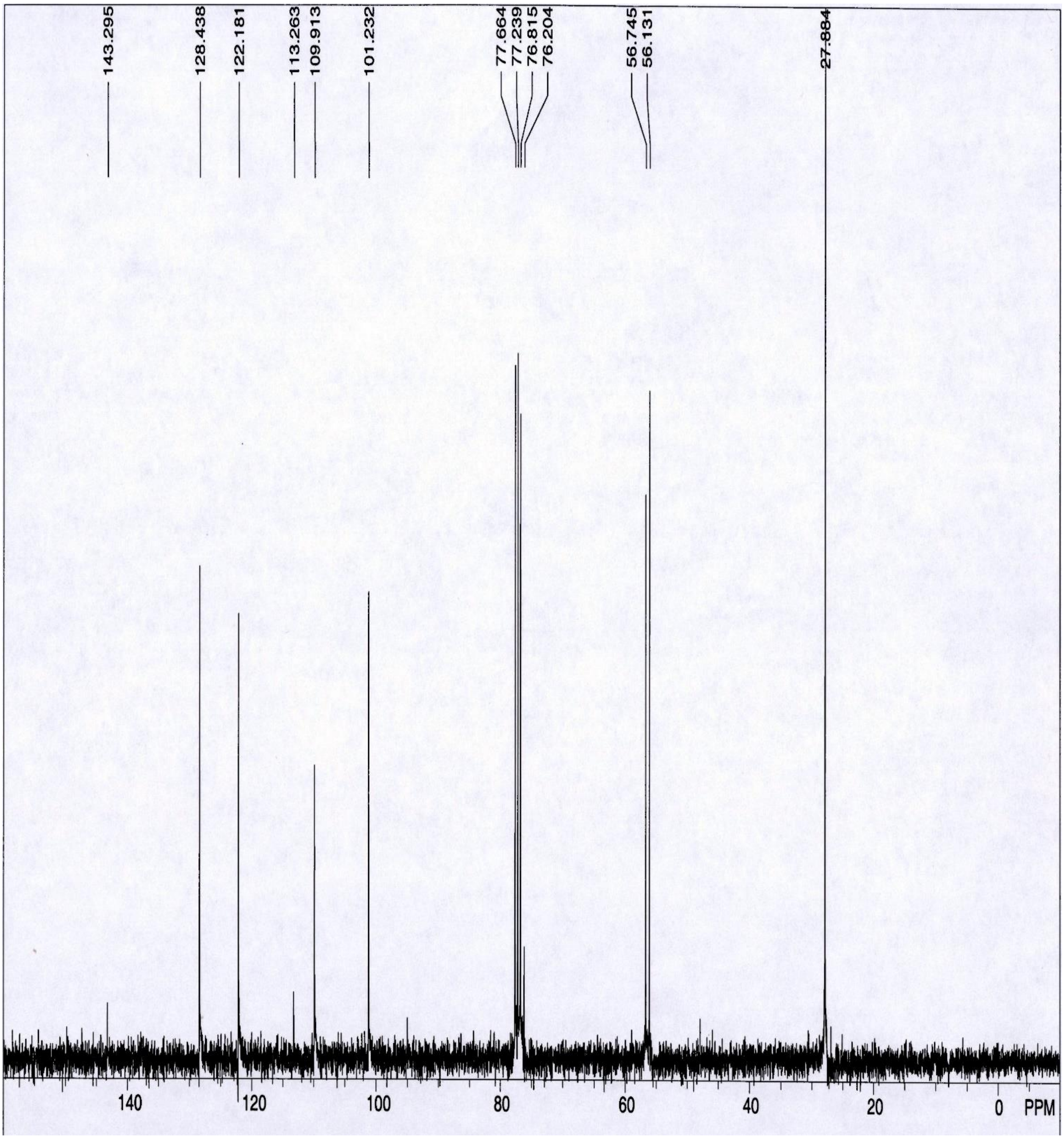

Supplementary Figure *S*41.- Mass spectrum of 5,7-dimethoxy, 2,2-dimethyl *2H*-

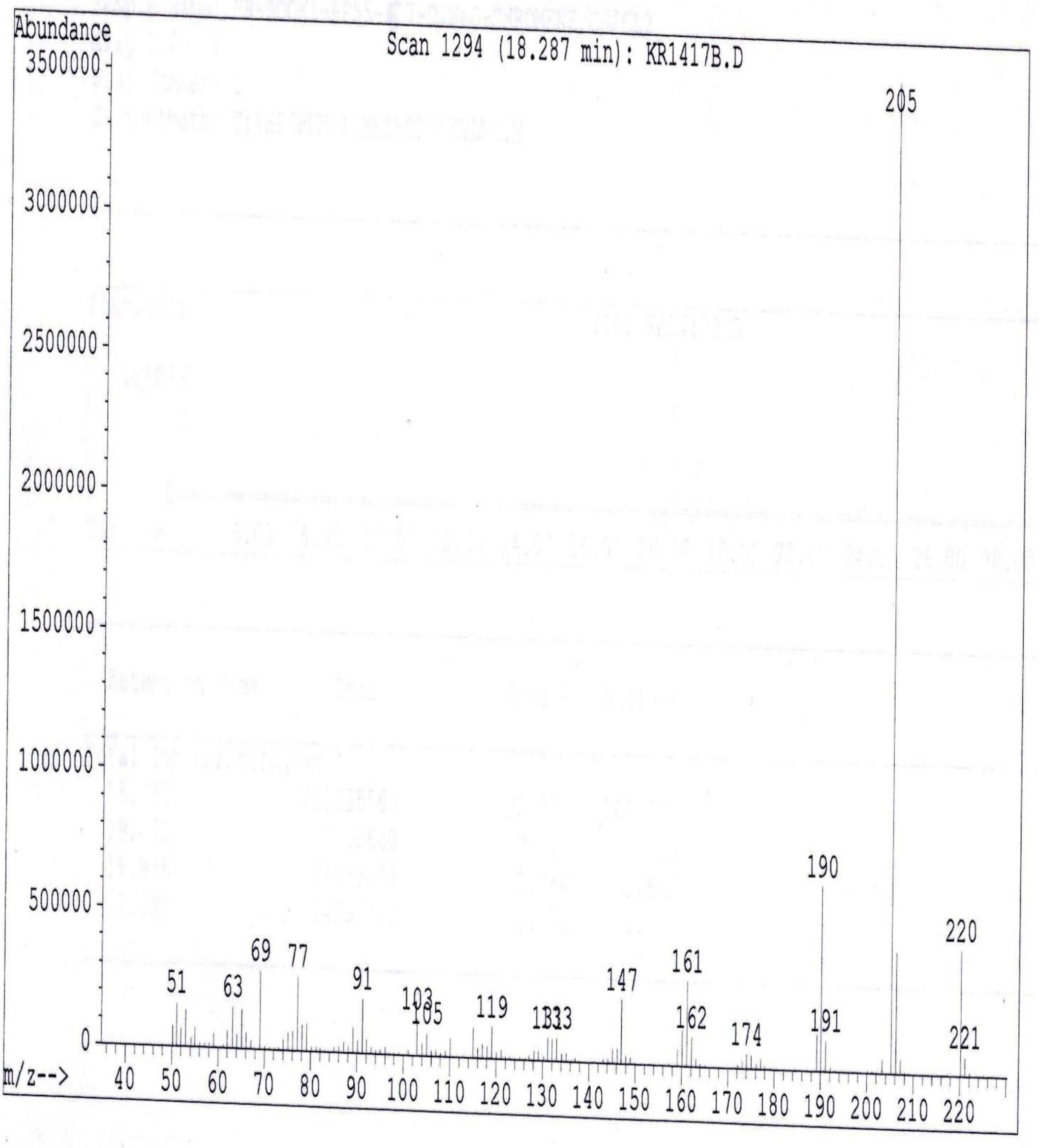

1-chromene (7c).

Supplementary Figure *S*42.- Proton chemical shift spectrum of 5,7-dimethoxy 2,2-dimethyl *2H*-1-chromene (7c)

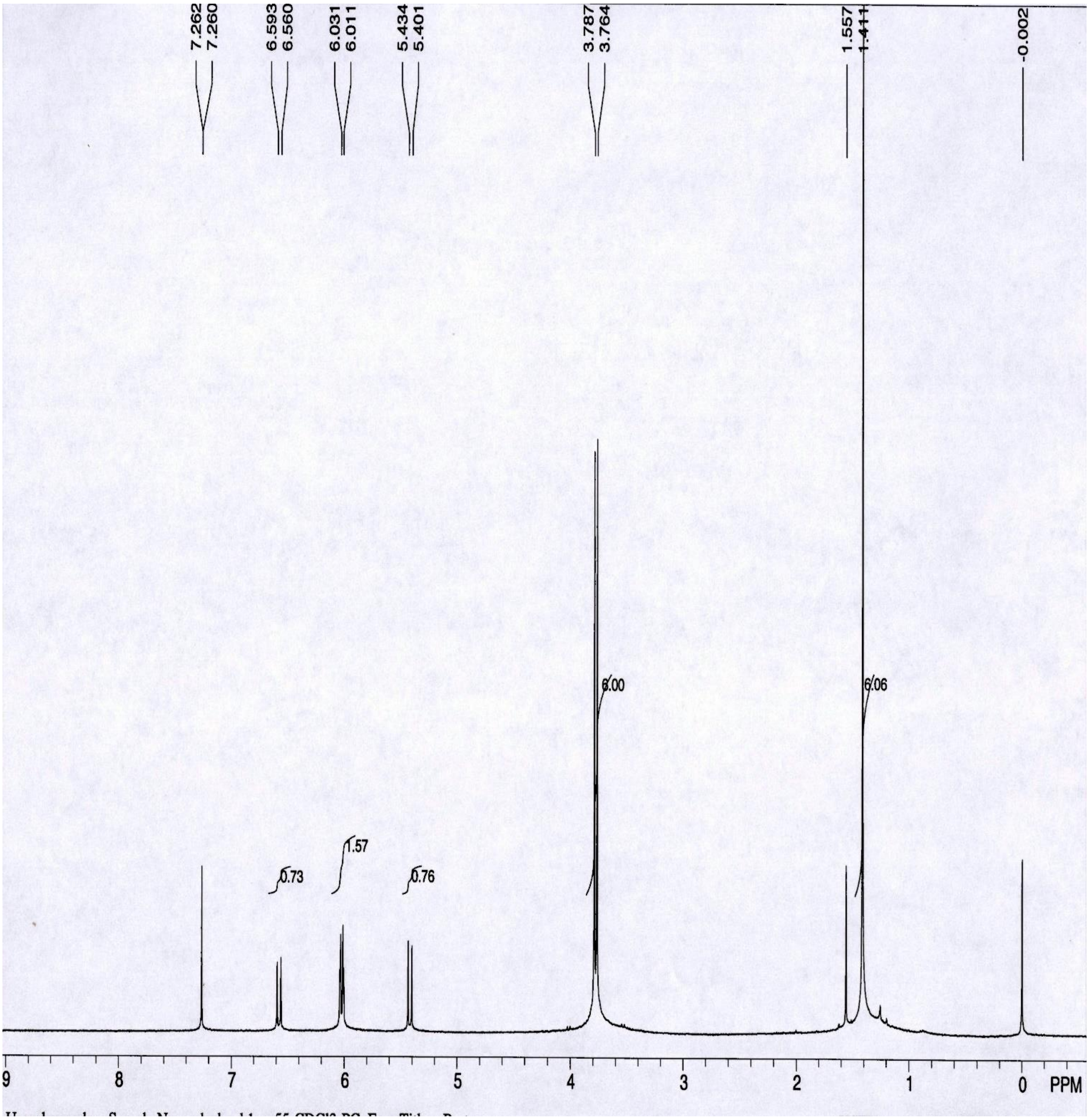

Supplementary Figure *S*43.- Mass spectrum of 7-ethoxy, 8-methoxy, 2,2-dimethyl *2H*-1-chromene (7d).

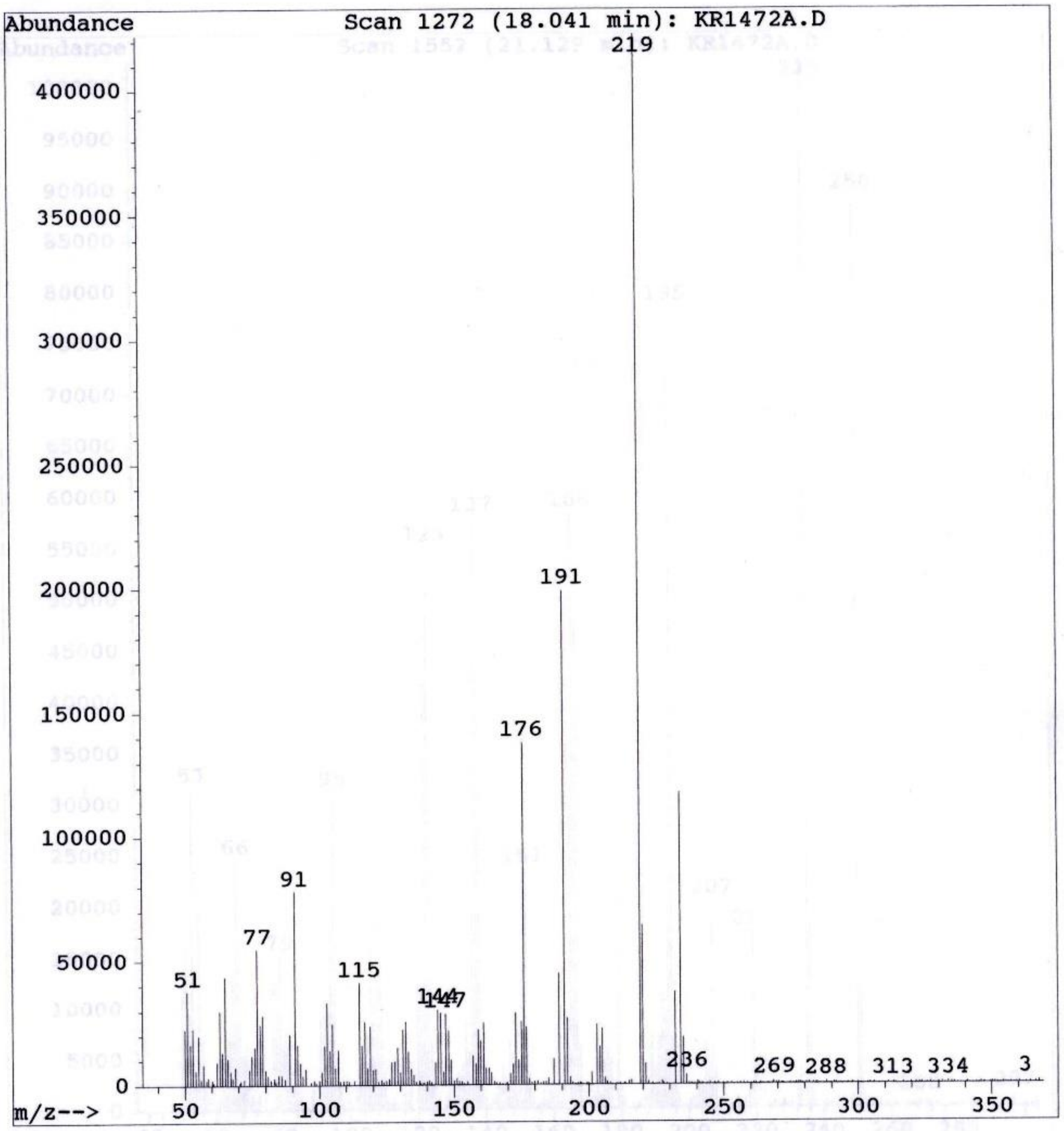

Supplementary Figure *S*44.- Proton chemical shift spectrum of 7-ethoxy, 8-methoxy 2,2-dimethyl *2H*-1-chromene (7d).

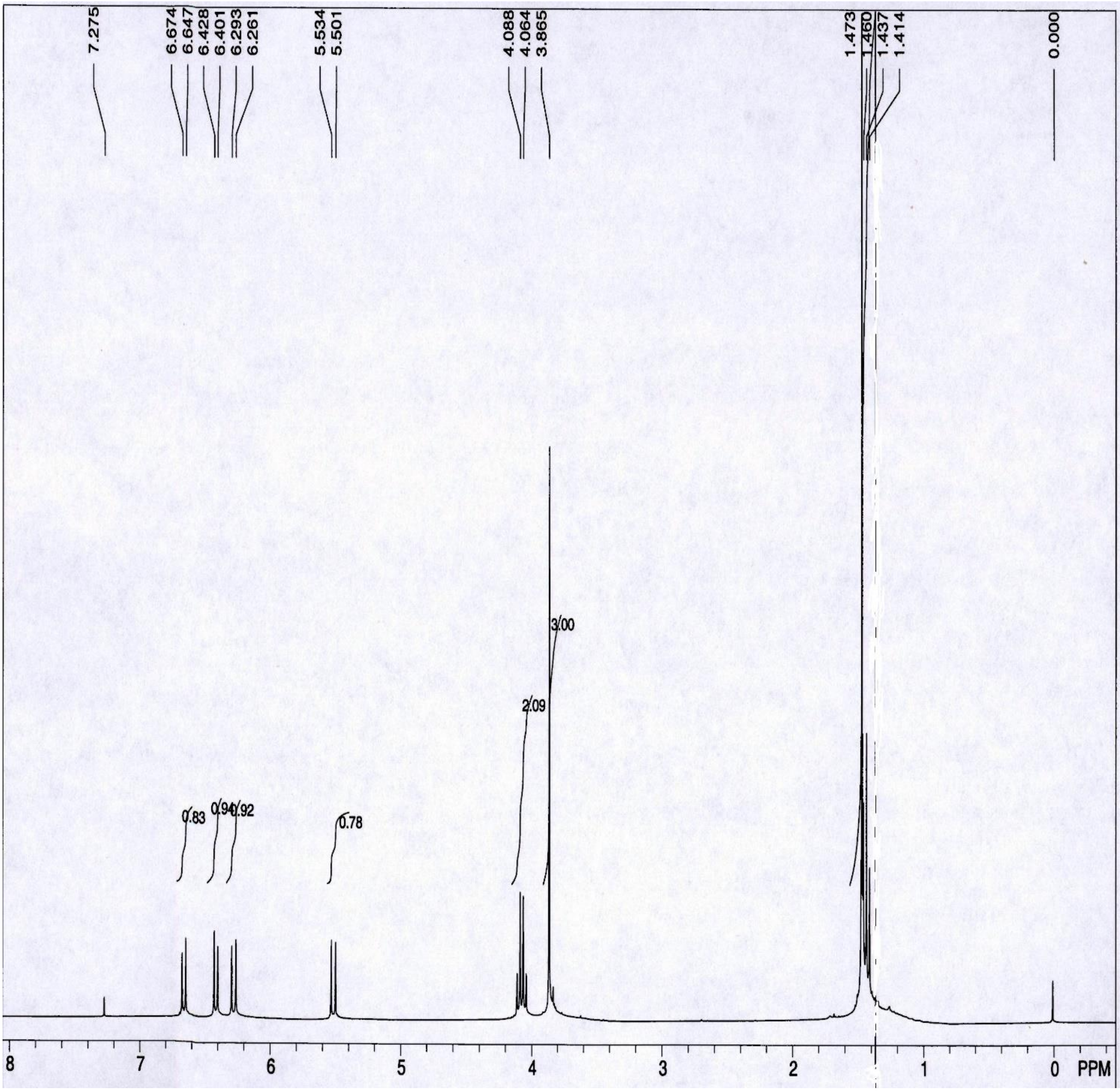

Supplementary Figure *S*45.- Carbon chemical shift spectrum of 7-ethoxy, 8-methoxy 2,2-dimethyl *2H*-1-chromene (7d).

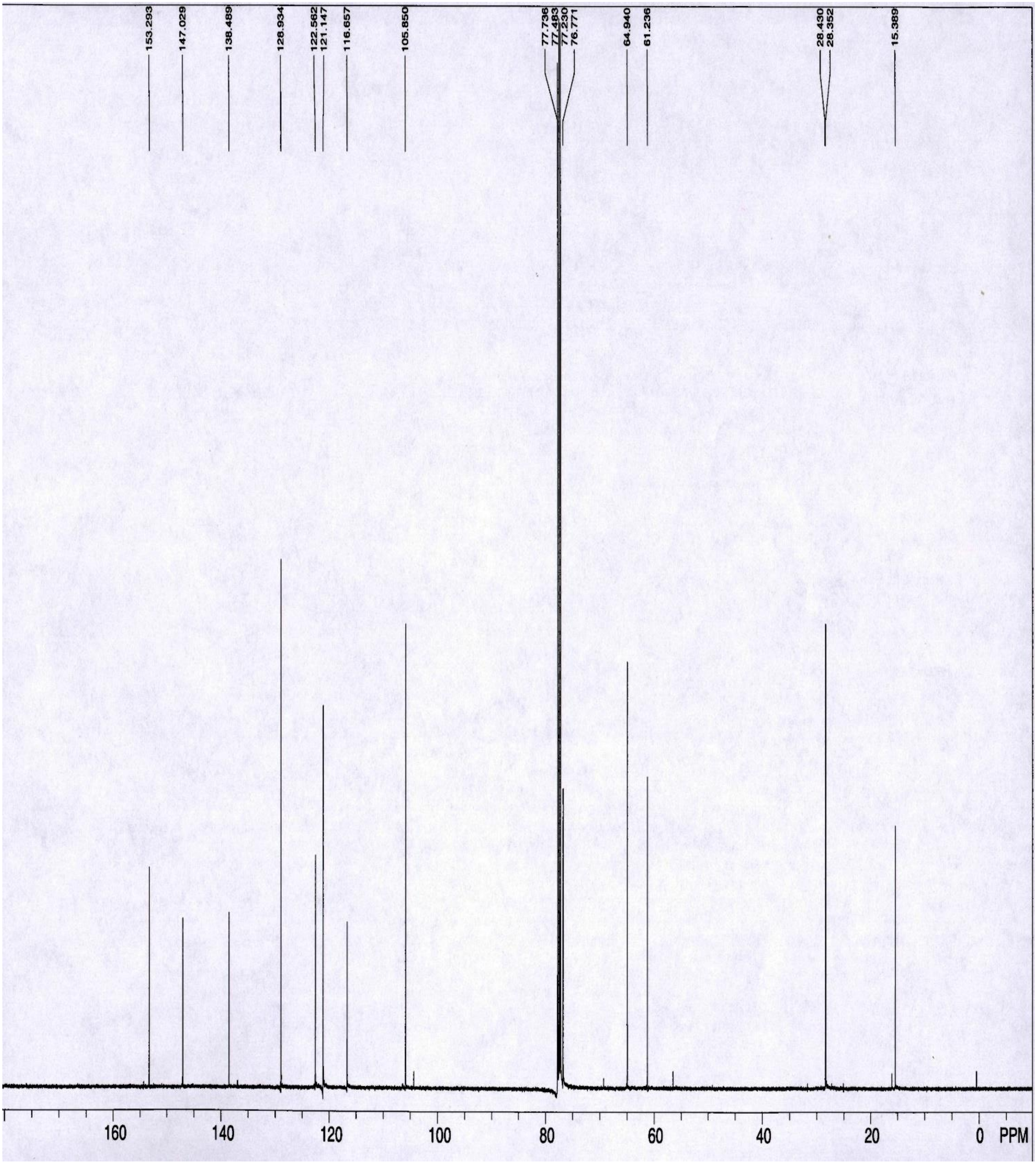

Supplementary Figure *S*46.- Mass spectrum of 7-ethoxy, 6-methoxy, 2,2-dimethyl *2H*-1-chromene (7e) (Precocene III).

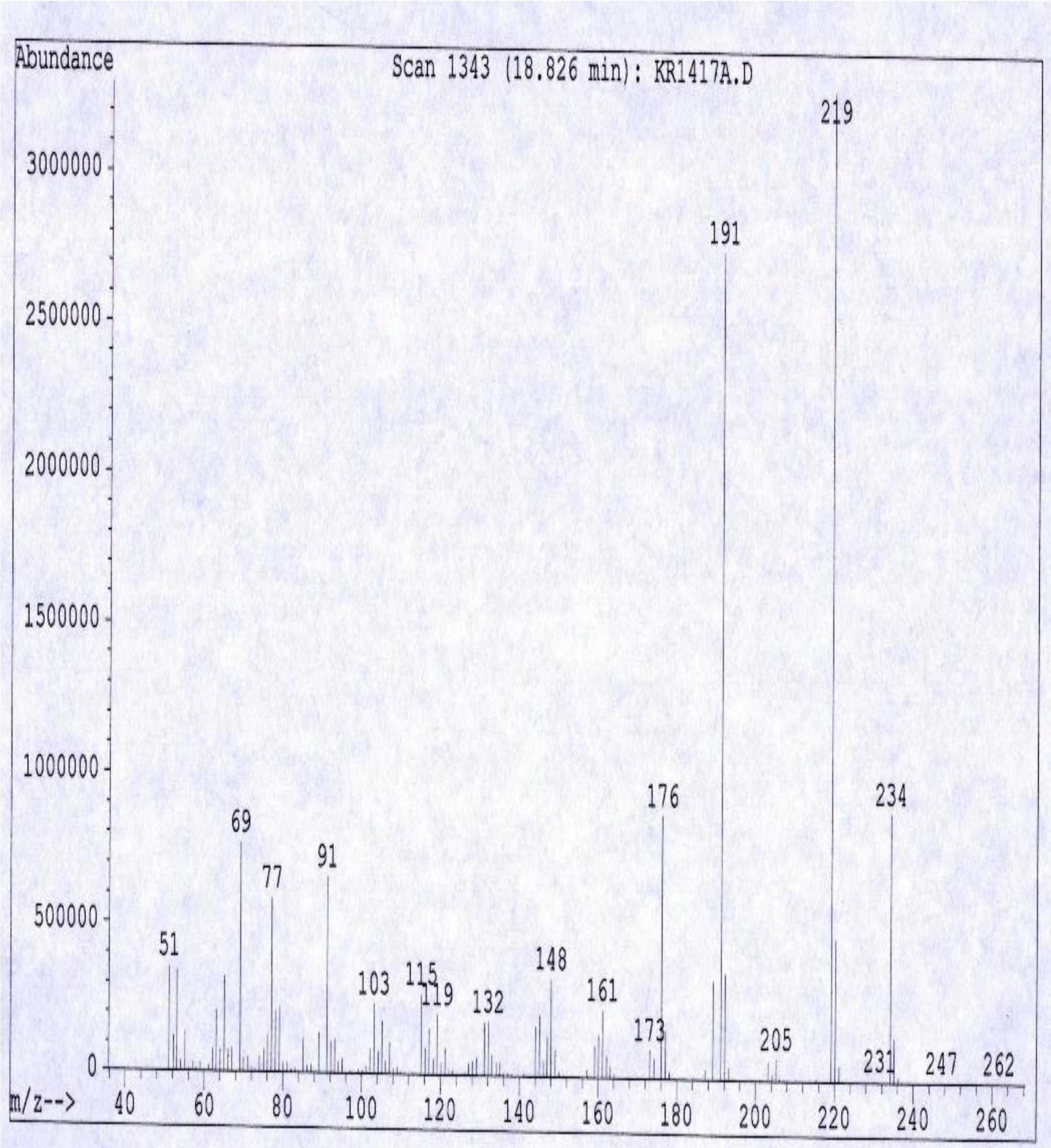

Supplementary Figure *S*47.- Proton chemical shift spectrum of 7-ethoxy, 6-methoxy, 2,2-dimethyl *2H*-1-chromene (7e) (Precocene III).

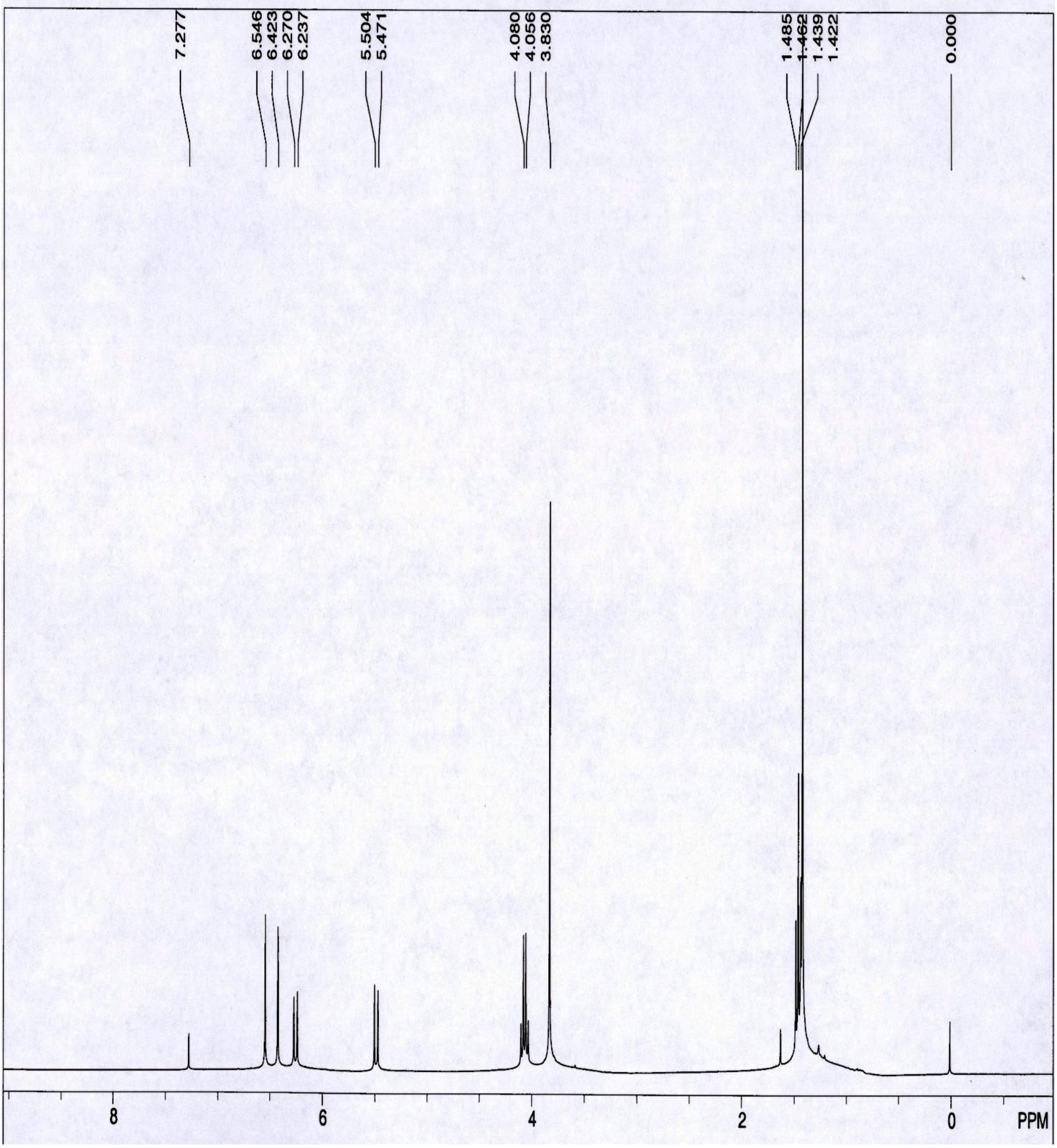

Supplementary Figure *S*48.- Carbon chemical shift spectrum of 7-ethoxy, 6-methoxy, 2,2-dimethyl *2H*-1-chromene (7e) (Precocene III).

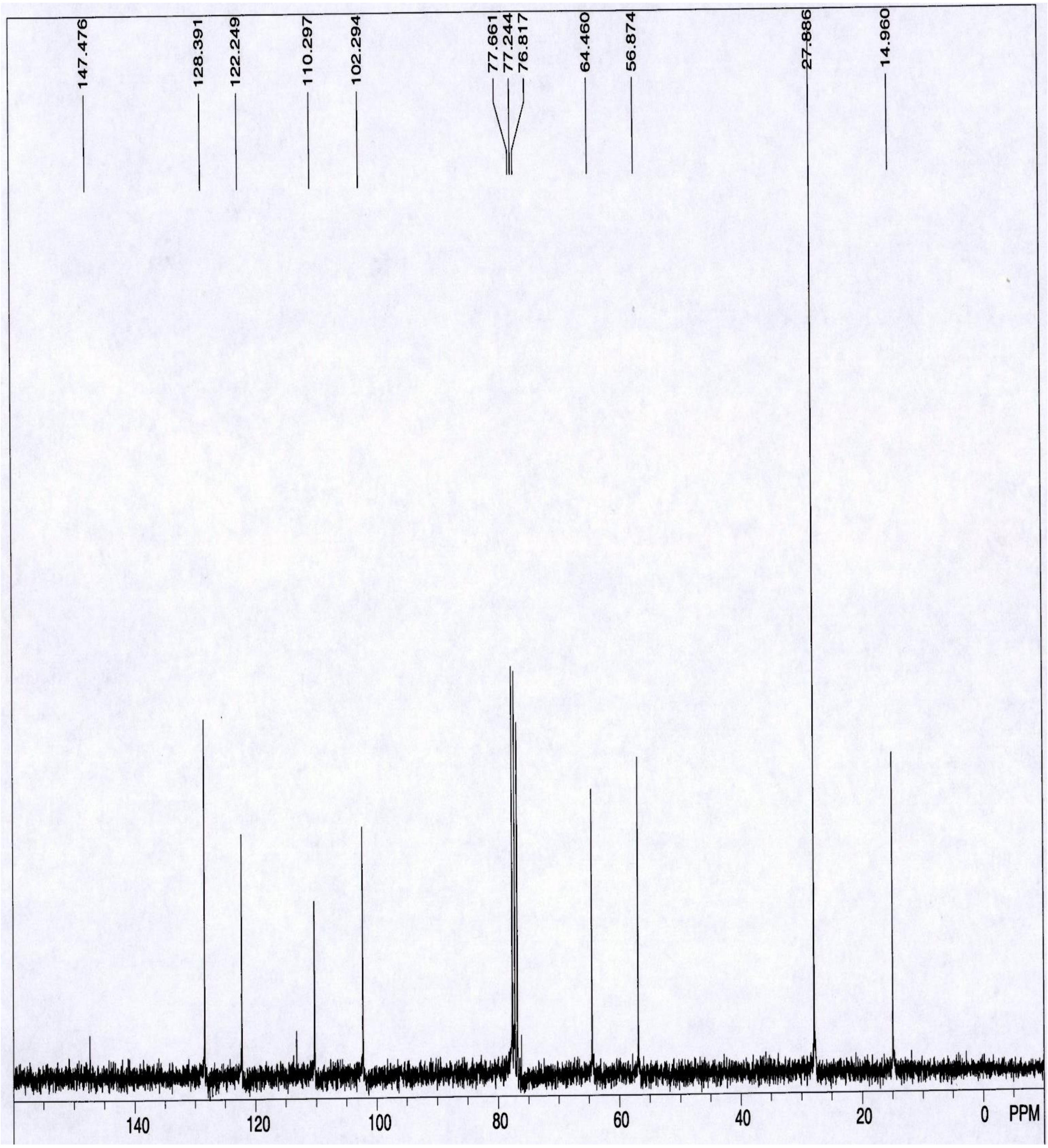

Supplementary Figure *S*49.- Mass spectrum of 7-ethoxy, 5-methoxy, 2,2-dimethyl *2H*-1-chromene (7f).

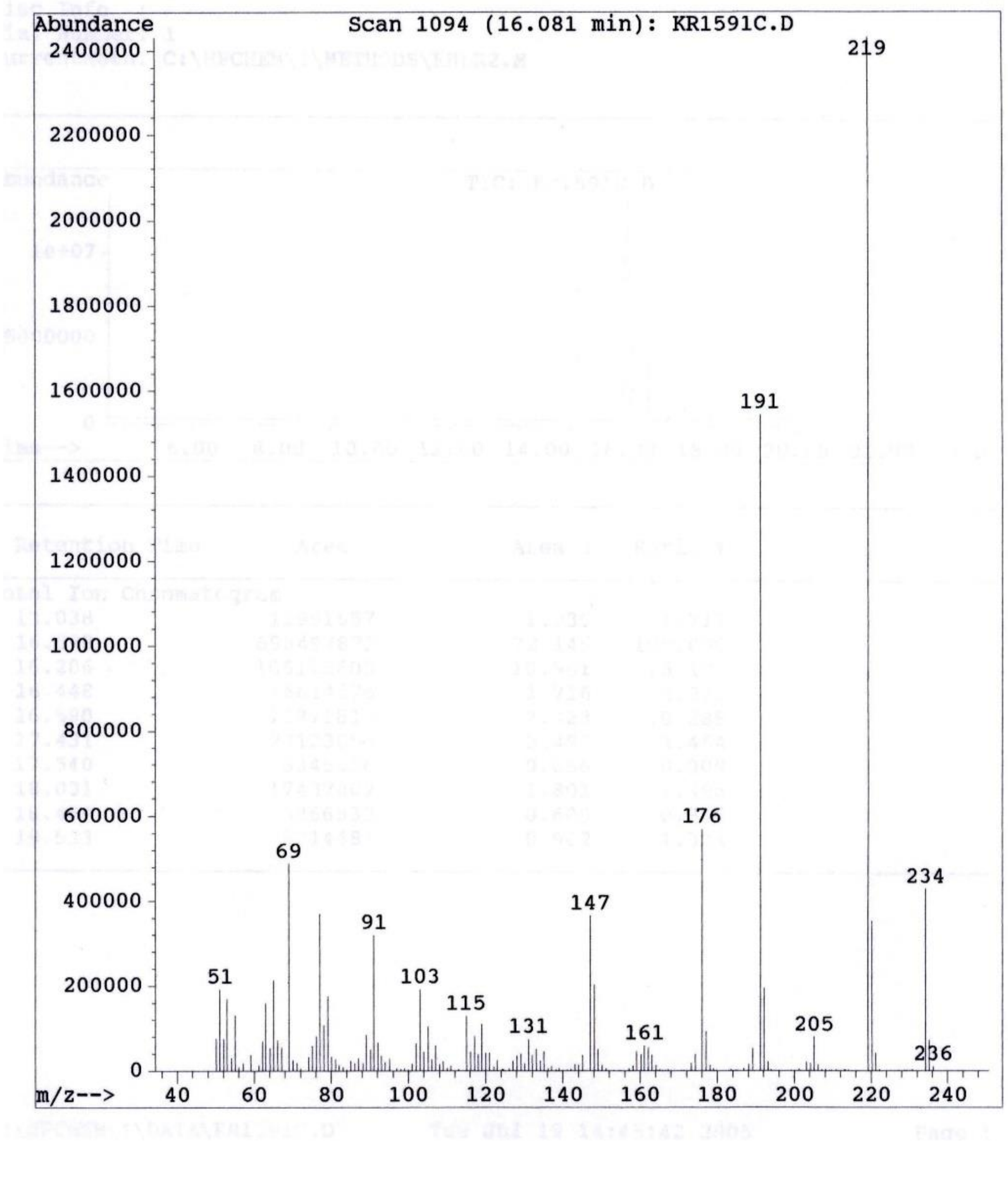

Supplementary Figure *S*50.- Proton chemical shift spectrum of 7-ethoxy,5-methoxy, 2,2-dimethyl *2H*-1-chromene (7f).

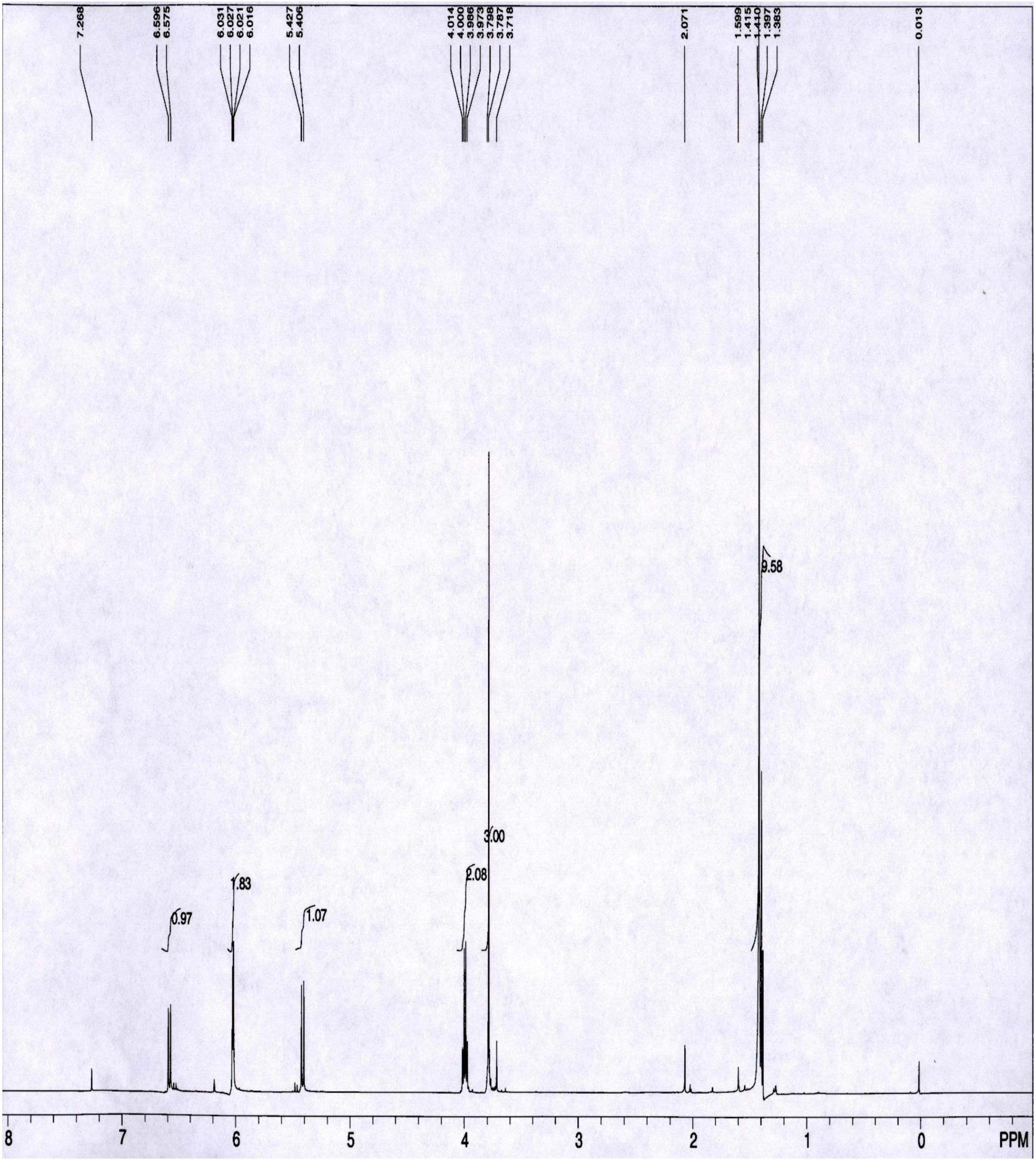

Supplementary Figure *S*51.- Carbon chemical shift spectrum of 7-ethoxy,5-methoxy, 2,2-dimethyl *2H*-1-chromene (7f).

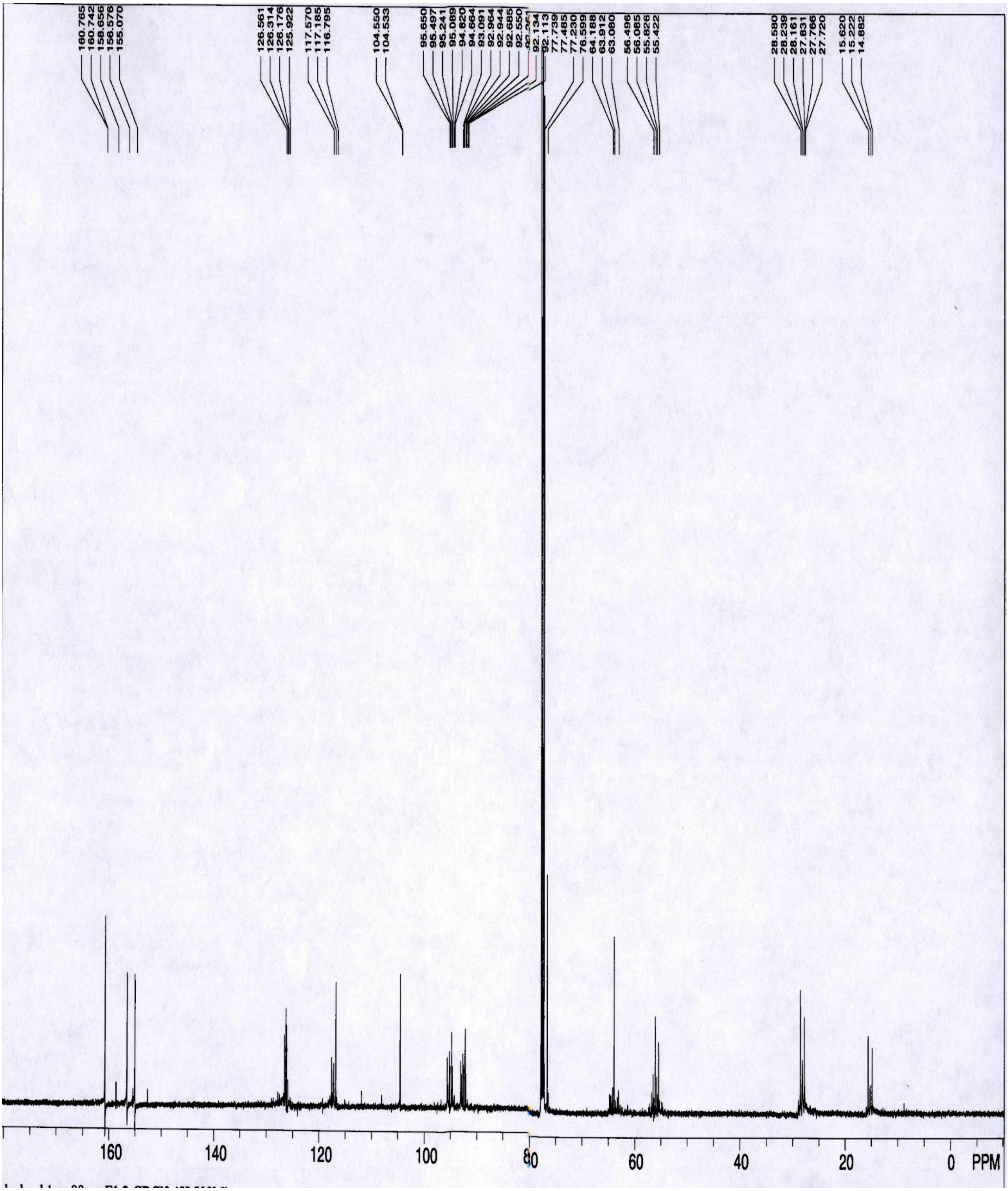

Supplementary Figure *S*52.- Mass spectrum of 1-(2`,4`-dihydroxyphenyl.-3-methyl-1- oxo-buta-2-ene (8).

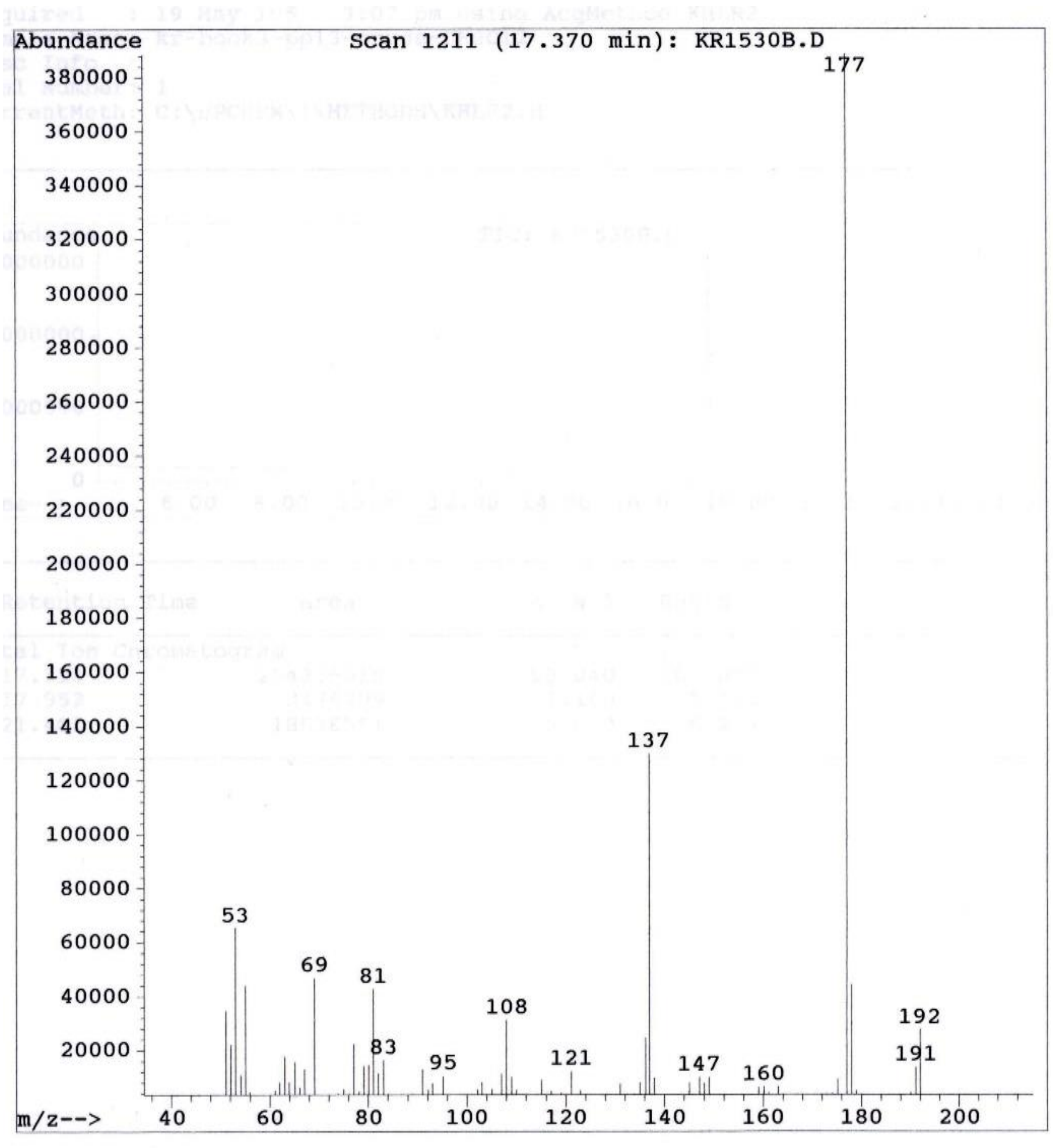

Supplementary Figure *S*53.- Proton chemical shift spectrum of 1-(2`,4`-dihydroxyphenyl.-3-methyl-1- oxo-buta-2-ene (8).

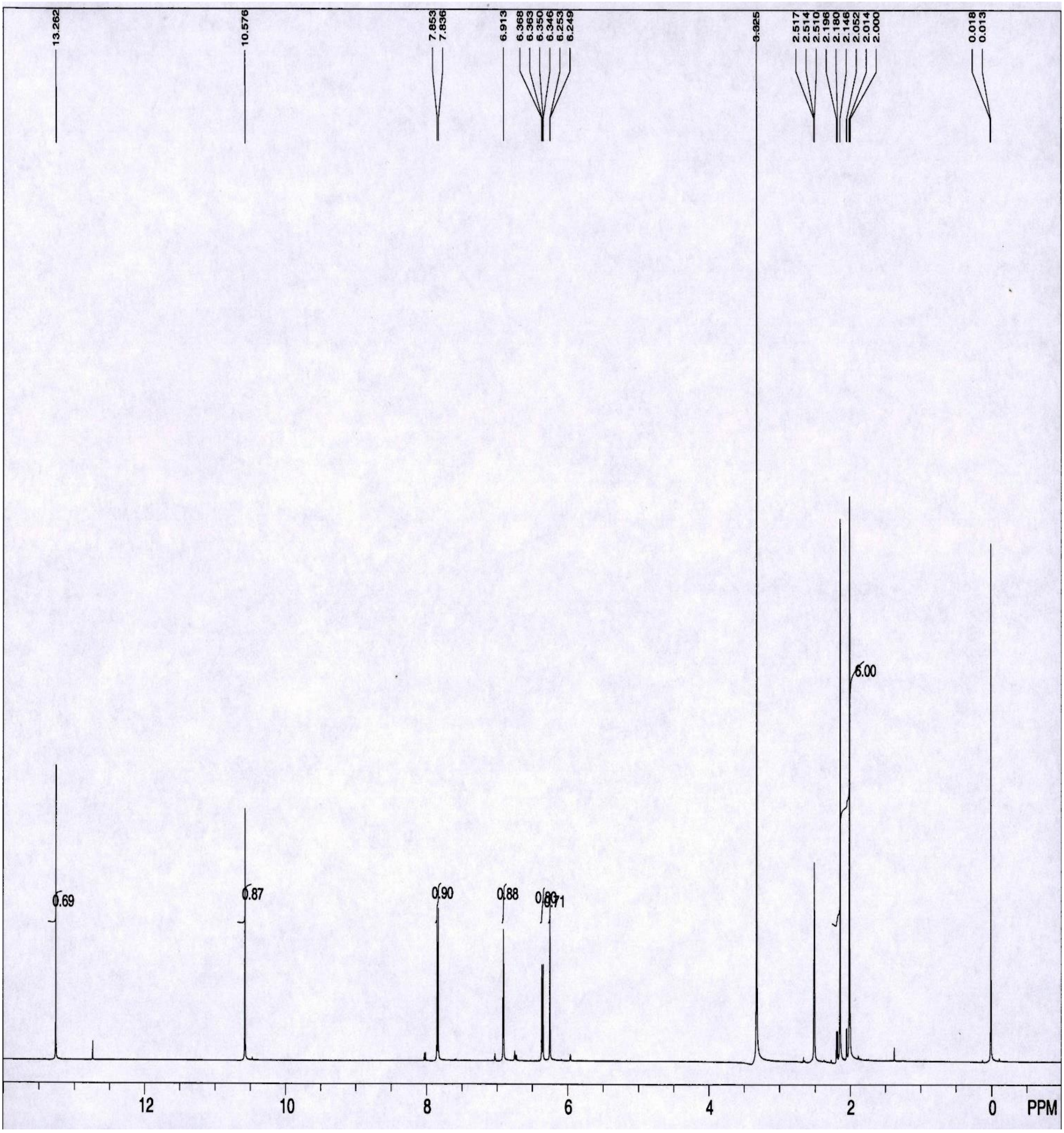

Supplementary Figure *S*54.- Mass spectrum of 7-hydroxy 2,2-dimethyl chroman-4-one (9).

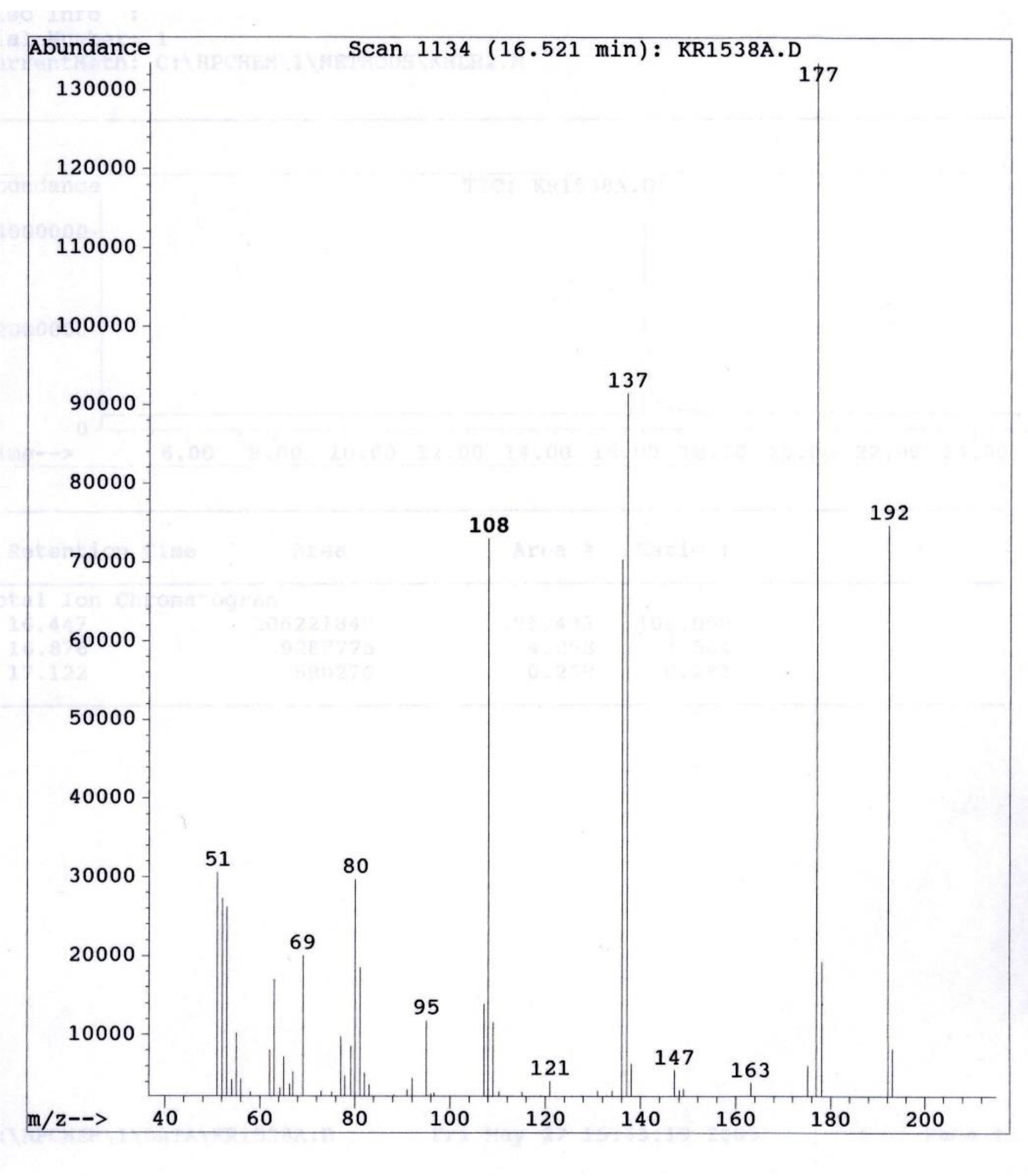

Supplementary Figure *S*55.- Proton chemical shift spectrum of 7-hydroxy 2,2-dimethyl chroman- 4-one (9).

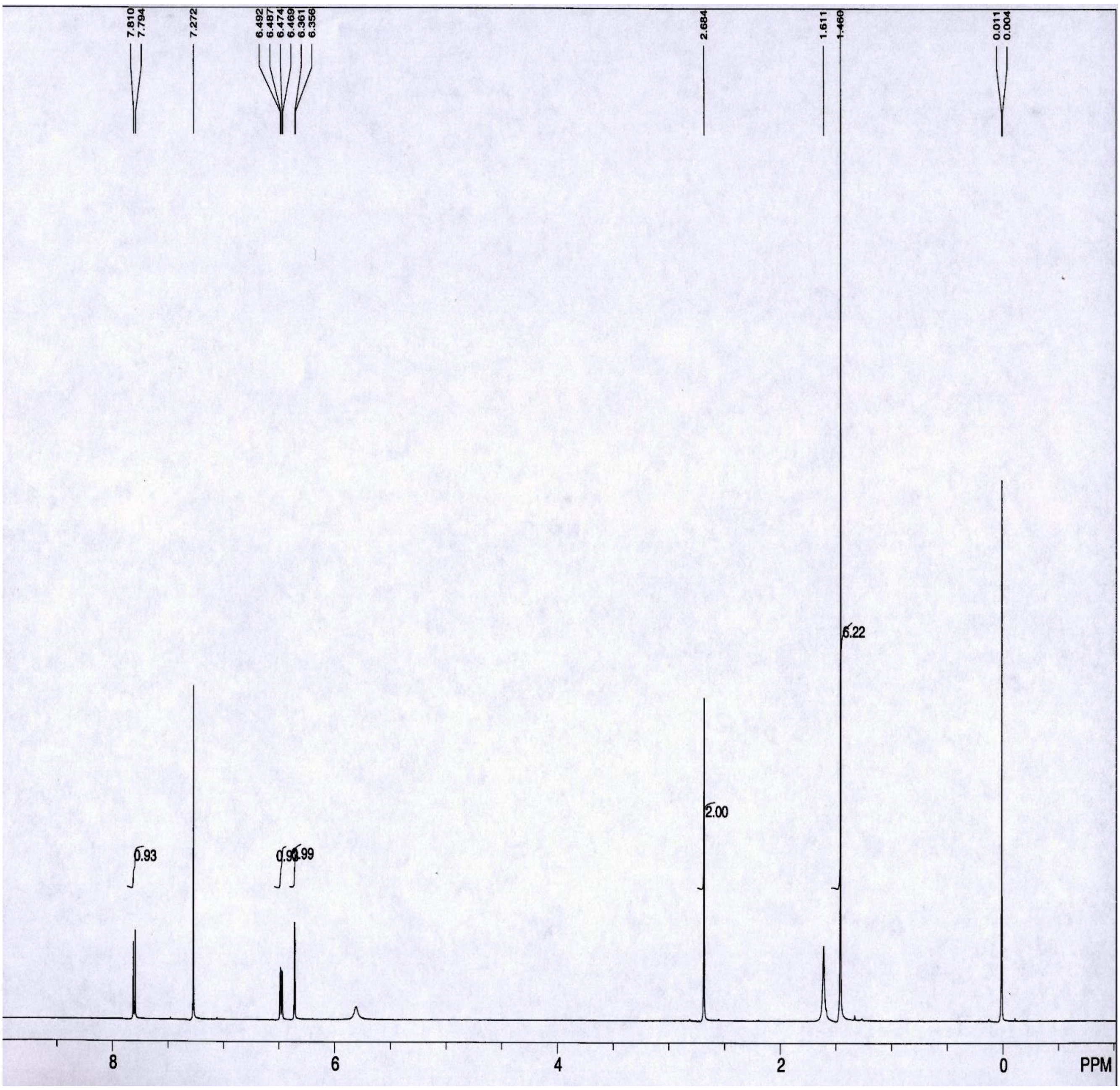

Supplementary Figure *S*56.- Mass spectrum of 7-methoxy 2,2-dimethyl chroman-4-one (10a).

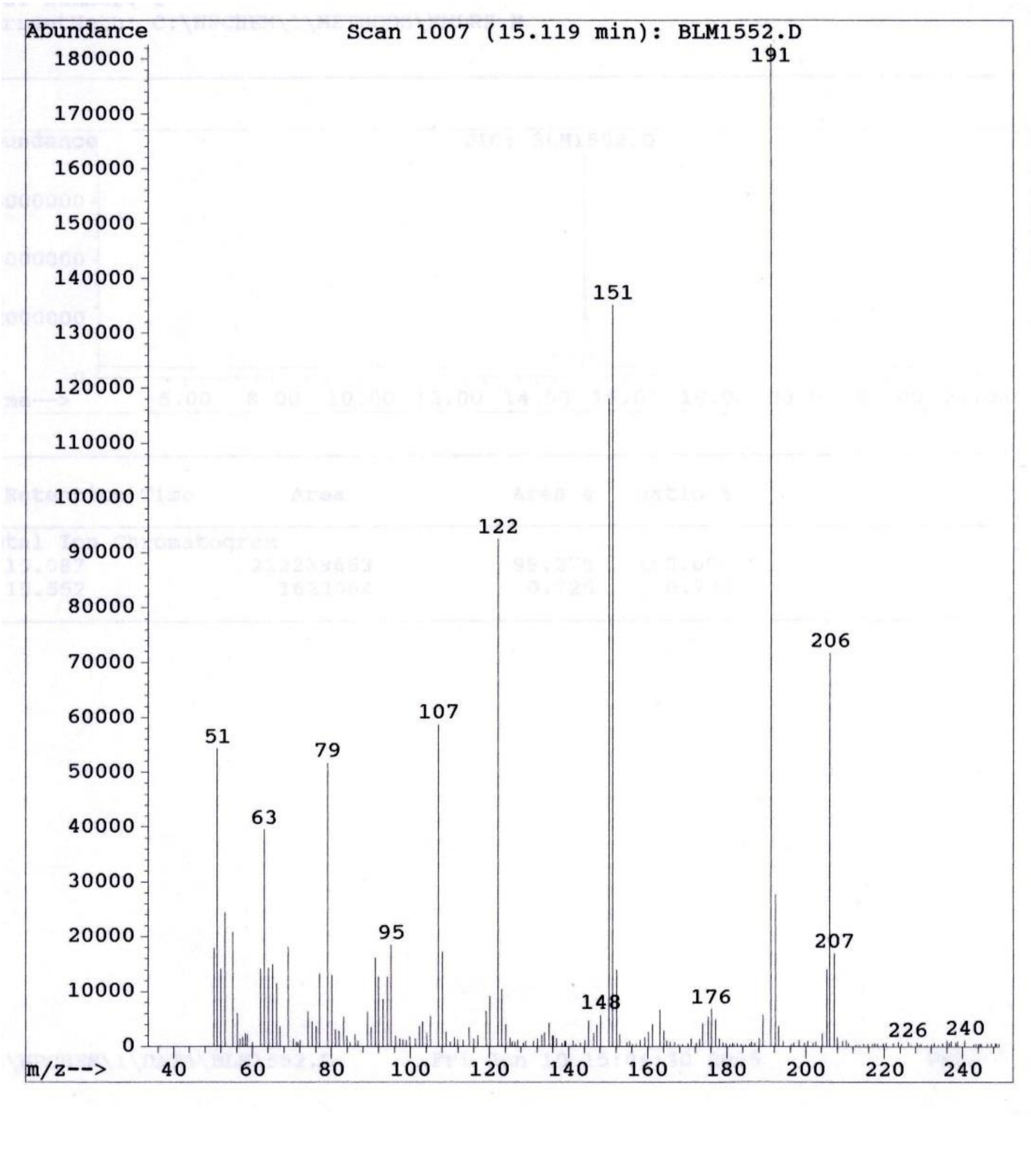

Supplementary Figure *S*57.- Proton chemical shift spectrum of 7-methoxy 2,2-dimethyl chroman-4-one (10a).

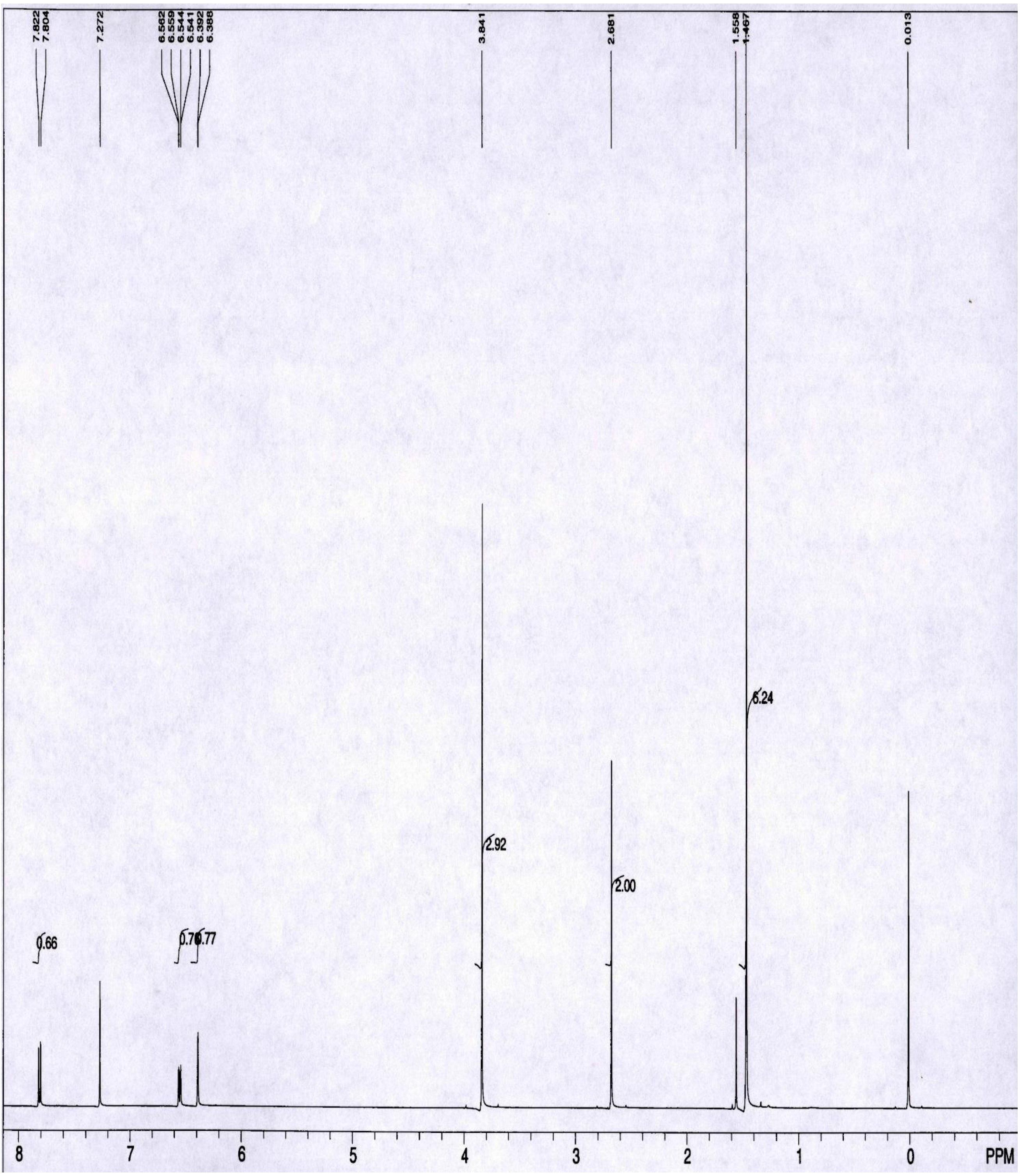

Supplementary Figure *S*58.- Mass spectrum of 7-ethoxy 2,2-dimethyl chroman-4-one (10b).

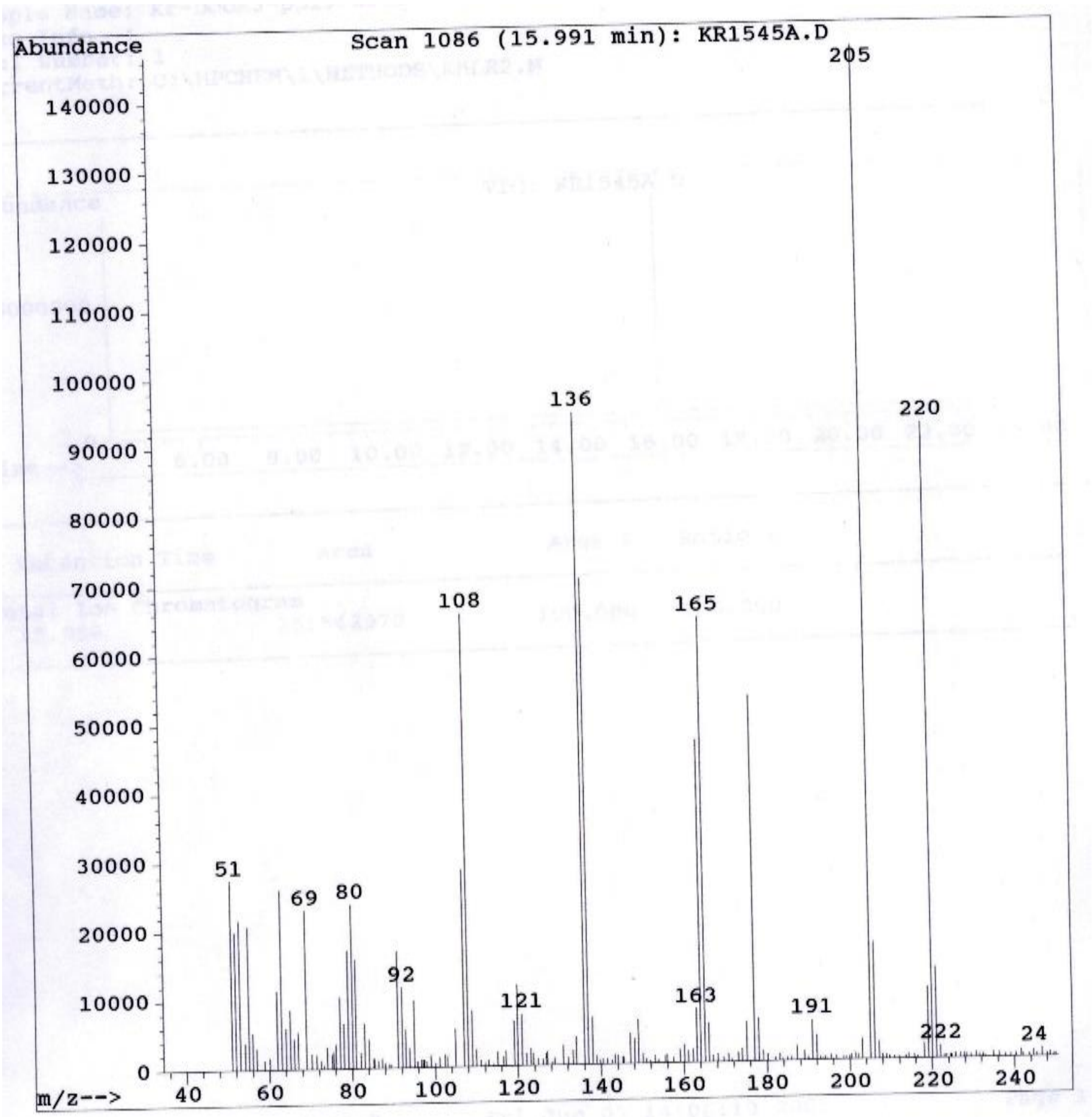

Supplementary Figure *S*59.- Proton chemical shift spectrum of 7-ethoxy 2,2-dimethyl chroman-4-one (10b).

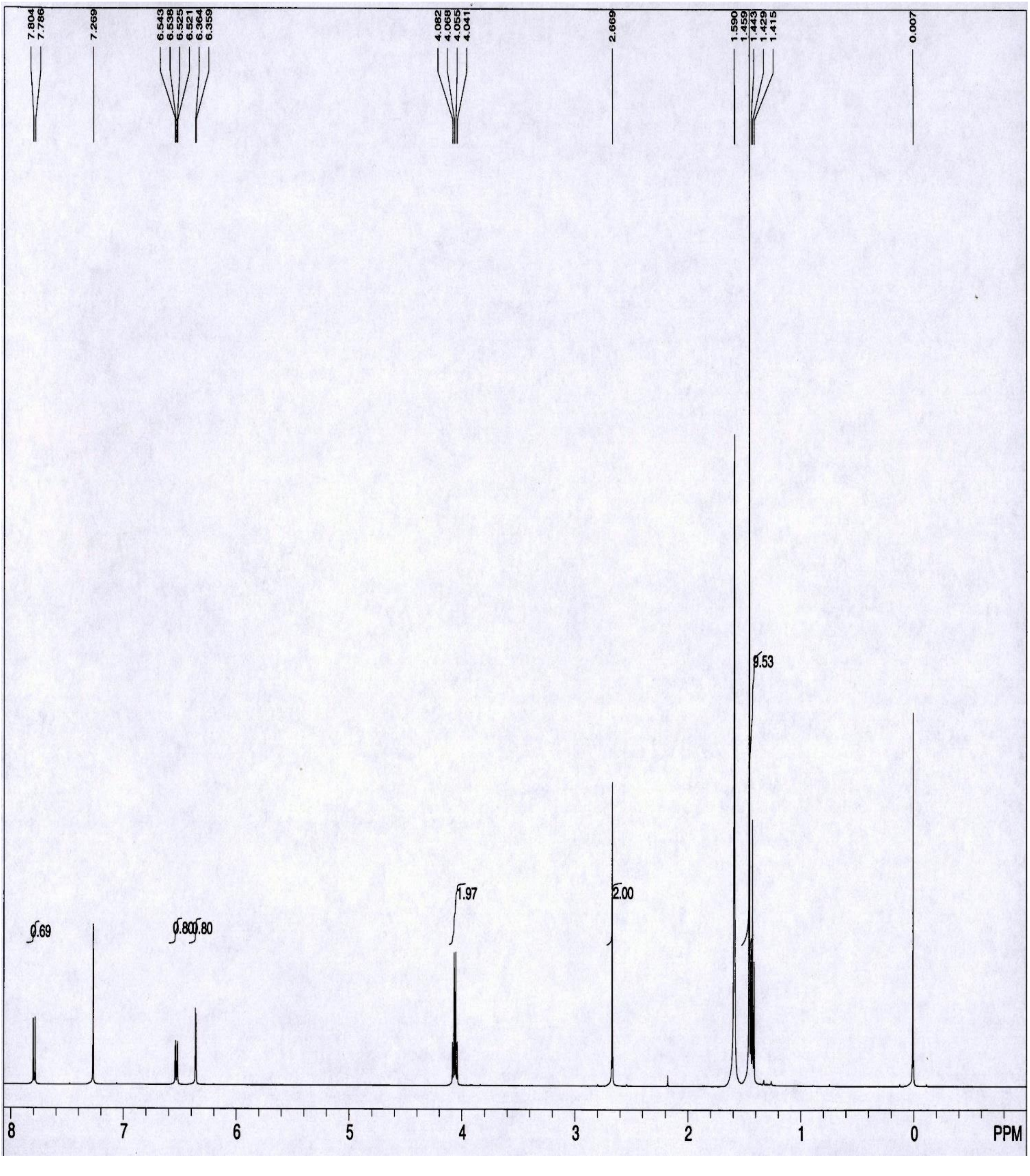

Supplementary Figure *S*60.- Mass spectrum of 7-methoxy 2,2-dimethyl *2H*-1-chromene (11a) (Precocene I).

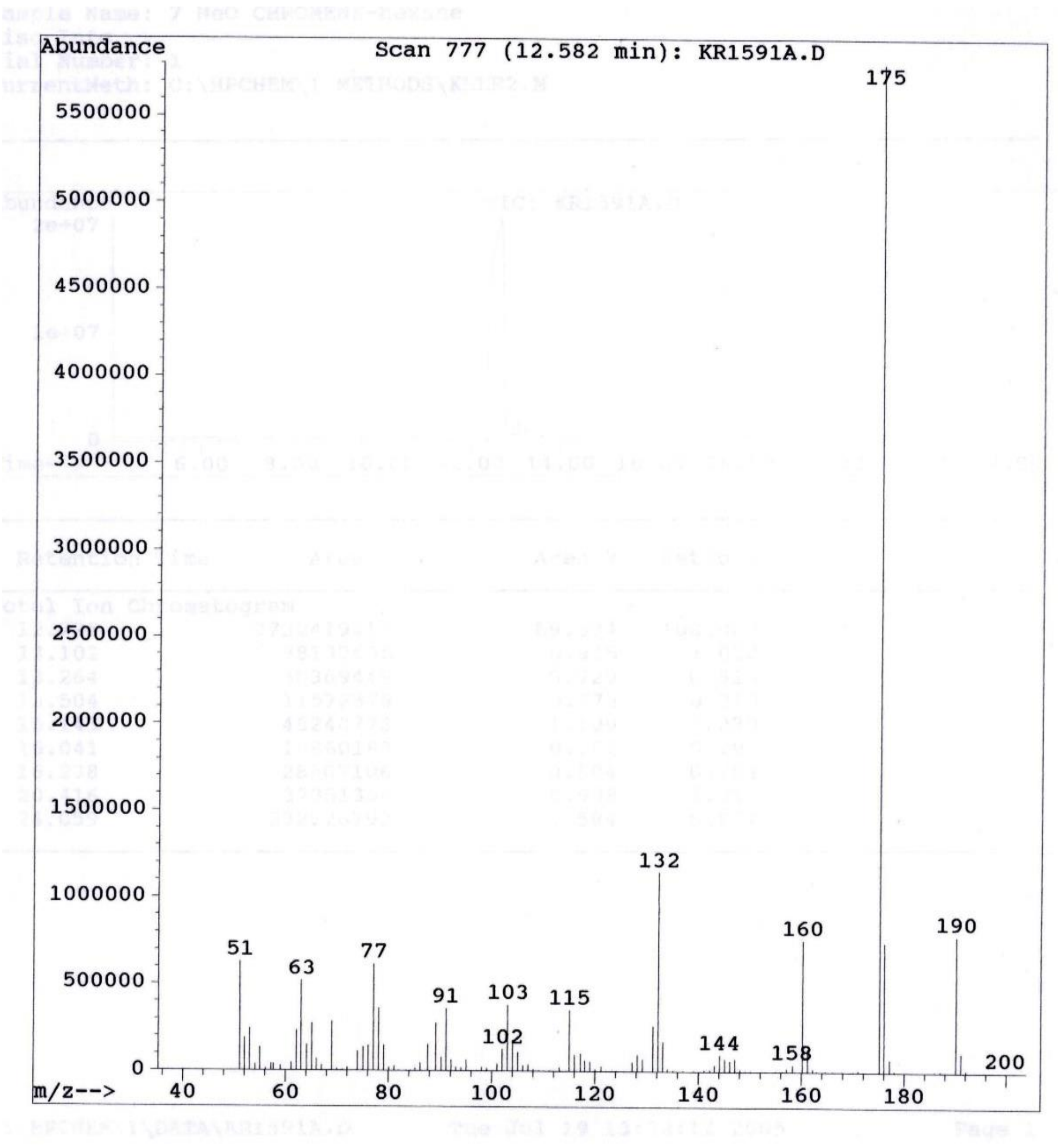

Supplementary Figure (61.- Proton chemical shift spectrum of 7-methoxy 2,2-dimethyl *2H*-1-chromene (11a) (Precocene I).

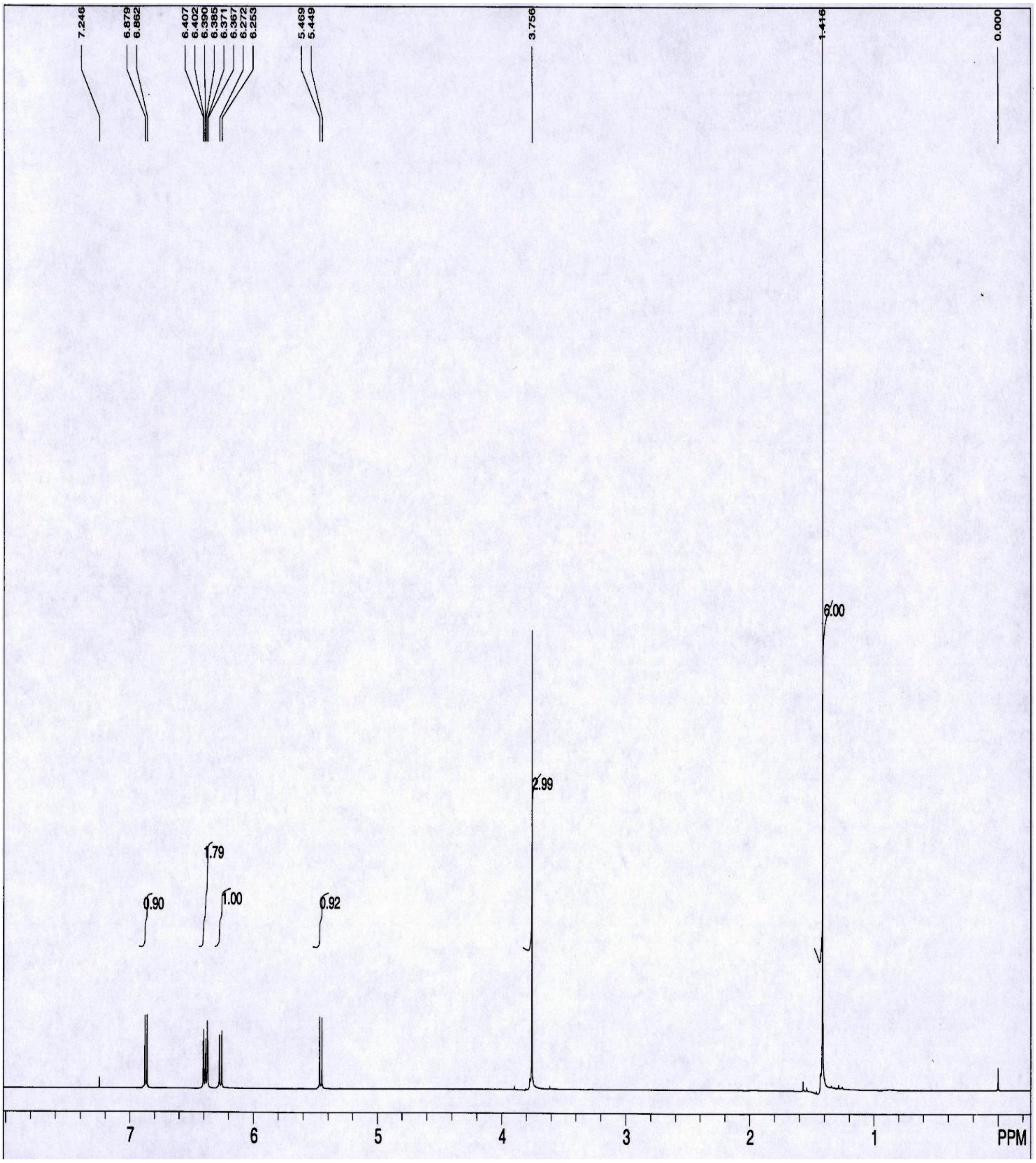

Supplementary Figure *S*62.- Carbon chemical shift spectrum of 7-methoxy 2,2-dimethyl *2H*-1-chromene (11a) (Precocene I).

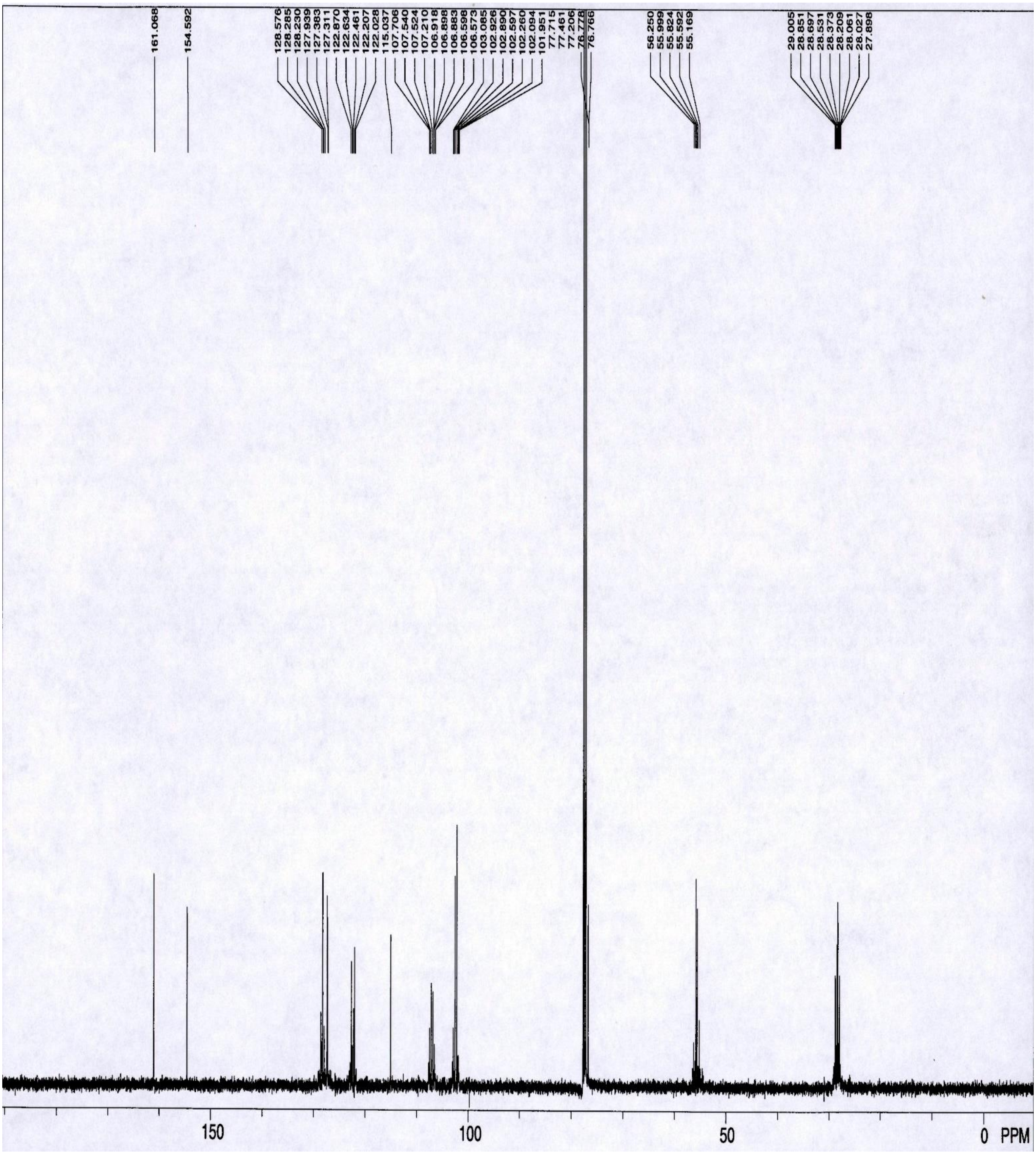

Supplementary Figure *S*63.- Mass spectrum of 7-ethoxy 2,2-dimethyl *2H*-1-chromene (11b).

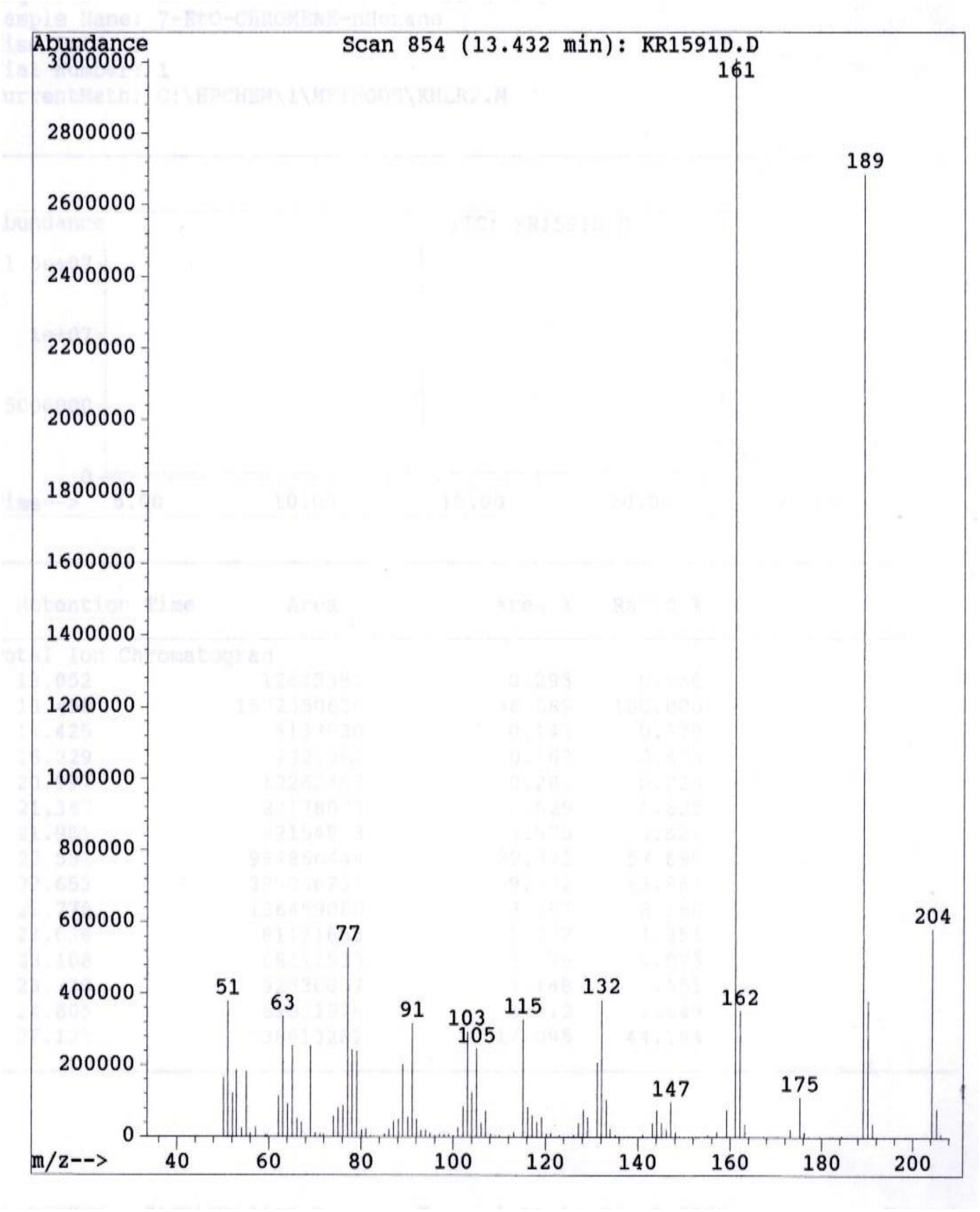

Supplementary Figure *S*64.- Proton chemical shift spectrum of 7-ethoxy 2,2-dimethyl *2H*-1-chromene (11b).

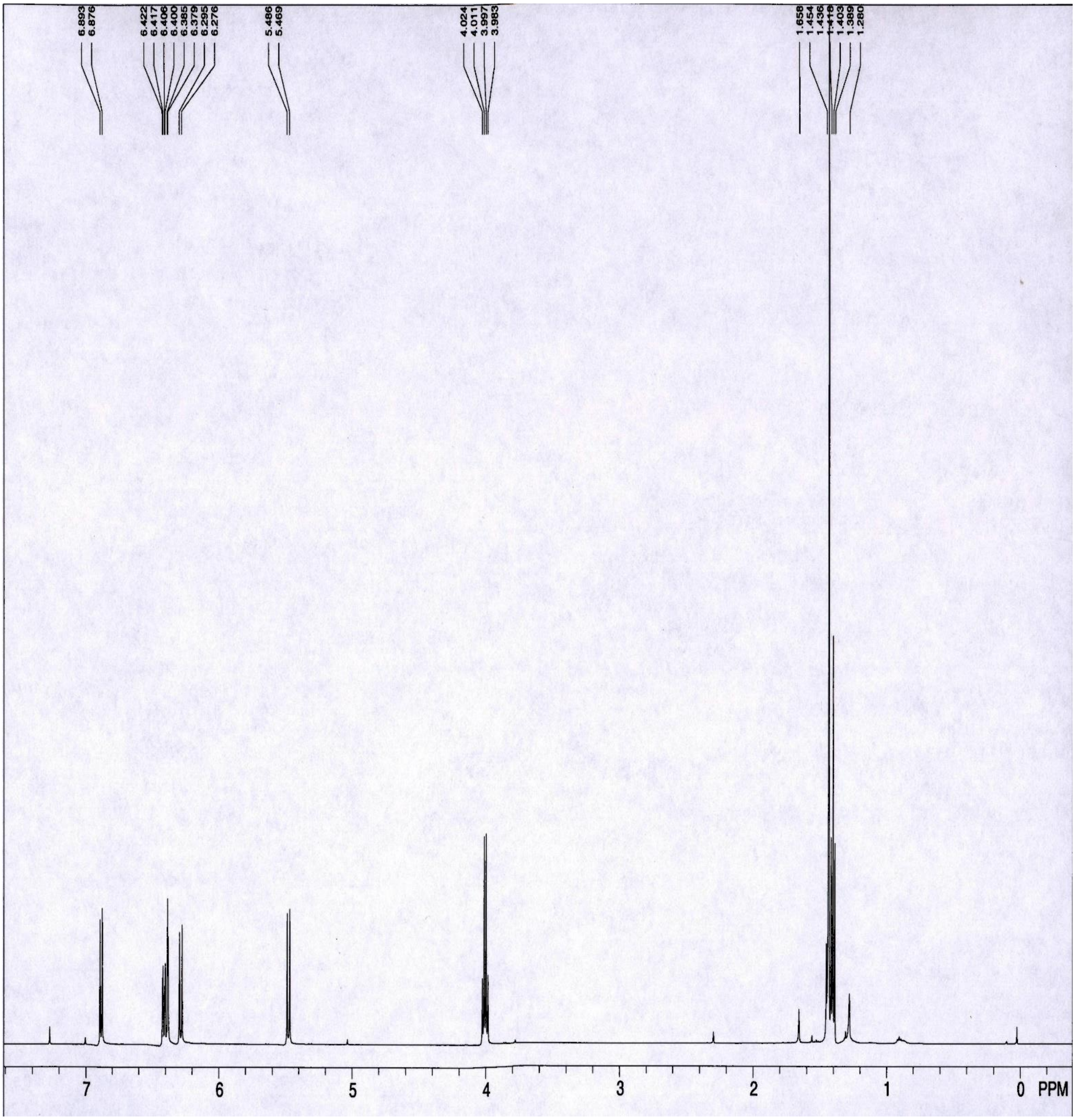

Supplementary Figure *S*65.- Carbon chemical shift spectrum of 7-ethoxy 2,2-dimethyl *2H*-1-chromene (11b).

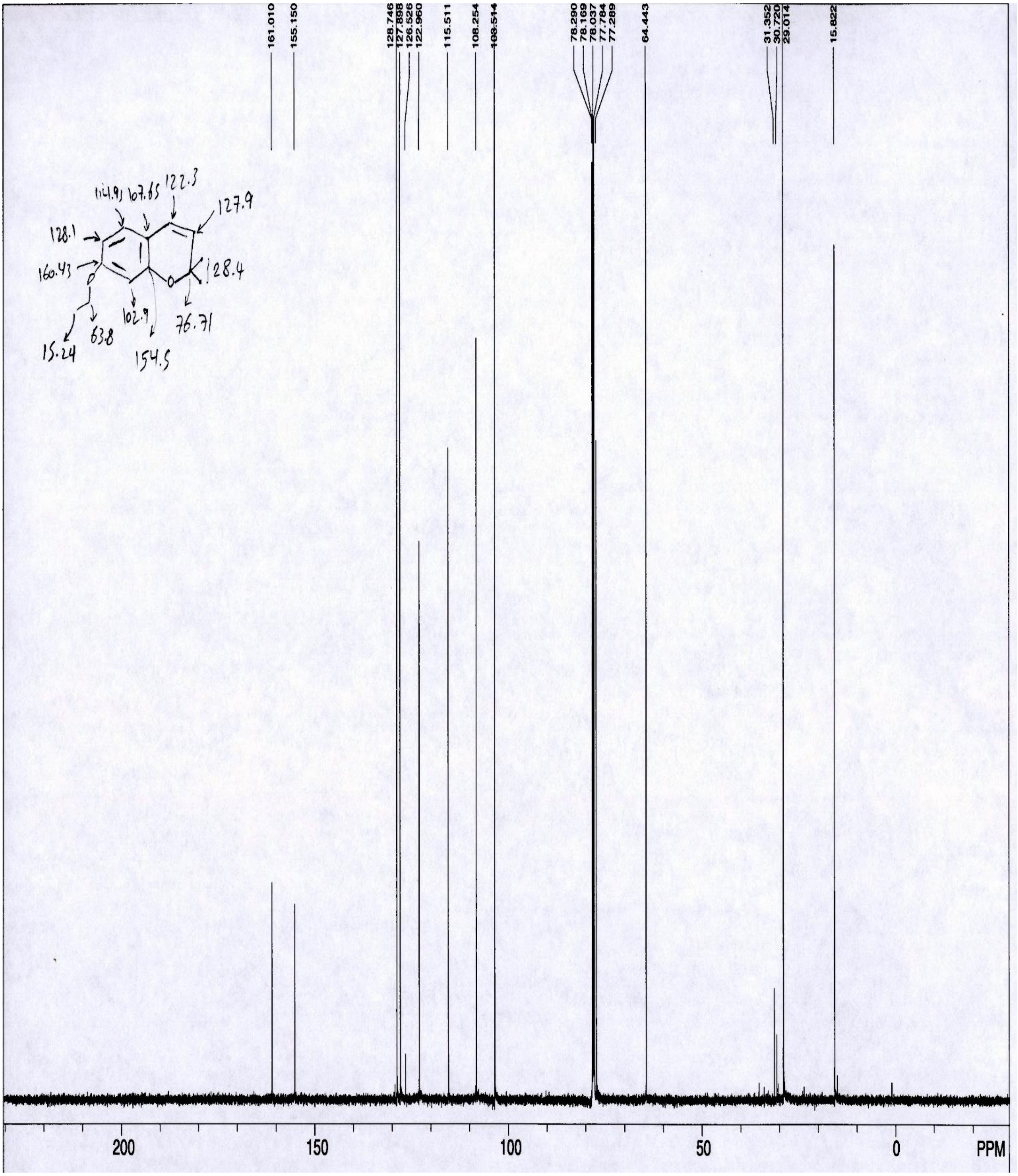

Supplement: Supplementary file 1 [file molecules-27-07177-s001.zip › molecules-1937046-supplementary.pdf]
